# Effectiveness of Dietary Management for Moderate Wasting among Children > 6 Months of Age—A Systematic Review and Meta-Analysis Exploring Different Types, Quantities, and Durations

**DOI:** 10.3390/nu15051076

**Published:** 2023-02-21

**Authors:** Bernardette Cichon, Jai K. Das, Rehana A. Salam, Zahra A. Padhani, Heather C. Stobaugh, Muzna Mughal, Patrizia Pajak, Alexandra Rutishauser-Perera, Zulfiqar A. Bhutta, Robert E. Black

**Affiliations:** 1 Action Against Hunger UK, Operations Department, London SE10 0ER, UK; 2 Institute for Global Health and Development, Aga Khan University, Karachi 74800, Pakistan; 3 Division of Women and Child Health, Aga Khan University, Karachi 74800, Pakistan; 4 The Daffodil Centre, The University of Sydney, Sydney 2006, Australia; 5 Action Against Hunger USA, Technical Services and Innovation Department, Washington, DC 20463, USA; 6 Friedman School of Nutrition Science and Policy, Tufts University, Boston, MA 02111, USA; 7 Centre for Global Child Health, Hospital for Sick Children, Toronto, ON M5G 0A4, Canada; 8 Bloomberg School of Public Health, Johns Hopkins University, Baltimore, MD 21205, USA

**Keywords:** moderate acute malnutrition, wasting, ready-to-use supplementary foods, ready-to-use therapeutic foods, fortified blended foods, corn–soy blend, supercereal

## Abstract

Currently, no World Health Organization guidelines exist for the management of approximately 31.8 million moderately wasted children globally. The objective of this review was to synthesise evidence on the optimal type, quantity, and duration of dietary treatment for moderate wasting. Ten electronic databases were searched until the 23rd of August 2021. Experimental studies comparing interventions for the dietary management of moderate wasting were included. Meta-analyses were conducted and results were presented as risk ratios or mean differences with 95% confidence intervals. Seventeen studies comparing specially formulated foods were included involving 23,005 participants. Findings suggest little or no difference in recovery between Fortified Blended Foods (FBFs) with improved micronutrient and/or milk content (enhanced FBFs) and lipid-based nutrient supplements (LNS), whereas children treated with non-enhanced FBFs (locally produced FBFs or standard corn–soy blend) may have lower recovery rates than those treated with LNS. There was no difference in recovery when ready-to-use therapeutic and ready-to-use supplementary food were compared. Other outcomes mostly aligned with results for recovery. In conclusion, LNSs improve recovery compared to non-enhanced FBFs, but are comparable to enhanced FBFs. Programmatic choice of supplement should consider factors such as cost, cost-effectiveness, and acceptability. Further research is required to determine optimal dosing and duration of supplementation.

## 1. Introduction

Childhood acute malnutrition, defined as a weight-for-height z-score (WHZ) < −2, a mid-upper arm circumference (MUAC) < 125 mm, or having kwashiorkor, is a major public health issue with recent prevalence estimates suggesting 6.7% or 45.4 million children under five worldwide had a WHZ < −2 in 2020 [1], a number that has likely increased following the COVID-19 pandemic [2]. The largest number of these children are those with moderate acute malnutrition (MAM), or moderate wasting defined as having a WHZ of <−2 to ≥−3 or MUAC of 115–125 mm (Traditionally, acute malnutrition is a term that encompasses both marasmus (or “wasting”) and kwashiorkor (oedematous malnutrition) forms of malnutrition. However, recently the term wasting is used synonymously with acute malnutrition, still encompassing both marasmus and kwashiorkor. For the purposes of this review, we use the wasting terminology synonymously with the acute malnutrition terminology). The true annual burden is unknown, as available estimates of 31.8 million do not take into account incidence or seasonal variation of wasting, and neither do they include children that are wasted according to the MUAC-based case definition. Addressing moderate wasting is critical in young children because of its short and long-term impacts on morbidity, development, productivity, and mortality. A total of 800,000–875,000 childhood deaths are attributed to wasting annually and 32-40% of these are due to moderate wasting [3].

Although treatment and therapeutic food composition guidelines exist for the treatment of severe wasting [4,5,6], there are currently no World Health Organization (WHO) guidelines for the treatment of moderate wasting. Approaches to managing moderate wasting differ by context, national protocols, and available resources but include breastfeeding promotion support, nutrition education, community-based social and behaviour change communication, and/or supplementary feeding with specially formulated or non-specially formulated foods [7].

In supplementary feeding programmes, children typically receive specially formulated foods in the form of fortified blended flours (FBFs) or lipid-based supplements (LNSs) dosed at approximately 75 kcal of food per kg of body weight per day or 500 kcal of food per day, which is given to children in addition to the child’s normal diet at the home. FBFs consist of corn, soy, or wheat flours with added micronutrients that need to be cooked with water before consumption. LNSs are ready-to-use foods (RUFs) consisting of lipid-based pastes (typically including ground peanuts) with added micronutrients that do not require refrigeration or preparation. Originally, such foods were developed for the treatment of severe wasting and, as such, are called ready-to-use therapeutic foods (RUTF). RUTFs were also shown to be effective in treating moderate wasting [8,9,10]. More recently, RUTFs were adapted for use in the management of moderate wasting and these products are referred to as ready-to-use supplementary foods (RUSFs).

In 2012, WHO published a technical note with a proposed nutrient composition for specially formulated foods in the treatment of moderate wasting and called for further research to generate an evidence base on the most appropriate food formulations [11]. Over the last decade, a variety of products have been created and modified in an attempt to better meet the estimated energy and nutrient needs of children with moderate wasting and/or to reduce costs [12,13,14,15]. For example, traditional FBFs such as CSBs used in moderate wasting treatment programs were enhanced by adding additional micronutrients and milk. Examples of enhanced FBFs include Supercereal, Supercereal+ or CSB++. Different RUSF formulations, with different sources of protein, have also been developed [13,14,15]. Still, the optimal quantity, quality, and source of protein in foods used for the treatment of moderate wasting is an ongoing topic of discussion [16].

A 2013 systematic review on the use of specially formulated foods for the management of moderate wasting concluded that both RUFs and FBFs are effective in the treatment of moderate wasting. Moreover, while RUFs led to higher recovery rates when compared to FBF, the two foods had similar results with regard to mortality, default and regression to severe wasting. Furthermore, the review found that enhanced FBFs, such as CSB++ in particular, may be equally effective to LNS [17].

Despite many developments in the management of moderate wasting, there is currently no consensus on a single best management approach, including whether children should receive specially formulated food as well as their composition and dosing. Furthermore, questions remain around which children in which contexts should receive specially formulated foods and whether moderate and severe wasting should be treated in one programme with the same supplement [18]. In recent years, several studies have investigated the effectiveness of dietary management of moderate wasting, including specially formulated foods with a range of compositions provided in varying quantities. The objective of this systematic review is to synthesise available evidence on dietary treatment of moderate wasting in infants and children aged > 6 months in terms of type, quantity, and duration to improve outcomes such as nutritional recovery to ultimately inform guidelines on wasting.

## 2. Materials and Methods

### 2.1. Inclusion and Exclusion Criteria

The Participant, Intervention, Comparators, Outcomes and Study Design (PICOS) criteria are shown in Table 1.

Studies assessing the effectiveness of various dietary management approaches for the treatment of moderate wasting were eligible for inclusion. More specifically, studies assessing the following sub-questions and comparisons were included:

- Sub-question 1: Effectiveness of fortified blended foods (FBFs) versus lipid-based nutrient supplements (LNS):
  - Comparison 1.a. Enhanced FBFs vs. LNSs;
  - Comparison 1.b. CSB vs. LNS;
  - Comparison 1.c. Locally produced FBFs versus LNS.

- Sub-question 2: Effectiveness of different compositions, doses or durations among different variations of the same type of specially formulated food (FBFs or LNS):
  ◦ Comparison 2.a. Comparisons of FBFs:
    - 2.a.1. Enhanced FBFs vs. locally produced FBFs;
    - 2.a.2. Supercereal without milk vs. Supercereal with milk.
  ◦ Comparison 2.b. Comparisons of LNS:
    - 2.b.1. RUSF vs. RUTF;
    - 2.b.2. RUSF without animal protein vs. RUSF with animal protein;
    - 2.b.3. RUSF vs. alternative RUSF;
    - 2.b.4. RUTF vs. alternative RUTF.

- Sub-question 3. Effectiveness of specially formulated foods versus non-specially formulated food.

**Figure 1:**
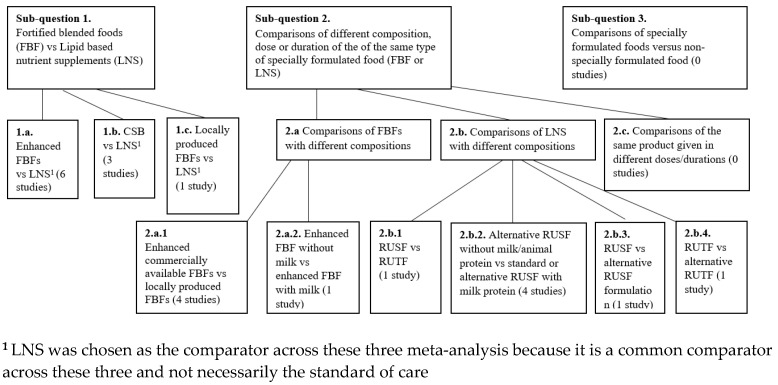
Flowchart showing the number of studies per comparison group.

An overview of each of the subquestions is shown in Figure 1.

**Figure 2:**
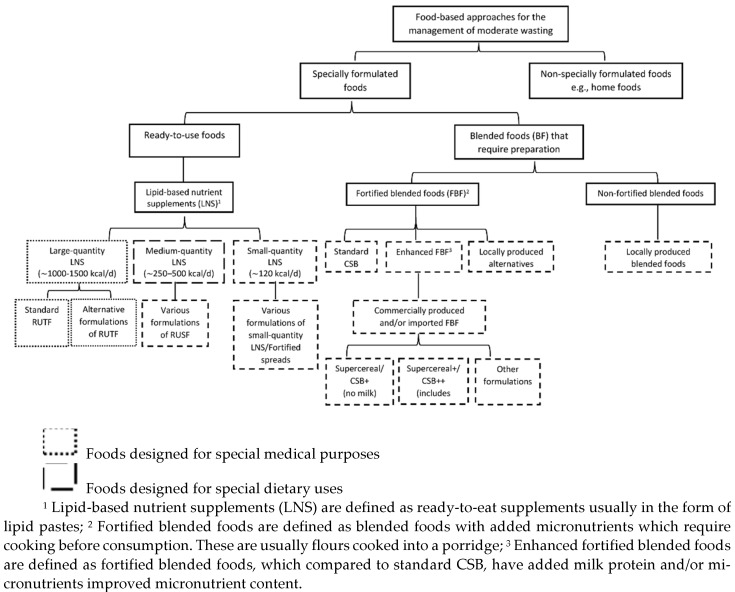
Flowchart of intervention categories.

Specially formulated foods were defined as foods specifically designed, manufactured, distributed, and used: (1) for special medical purposes, i.e., foods that are to be distributed under medical supervision such as RUTF or (2) for special dietary uses, as defined by the Codex Alimentarius for International Foods, i.e., those that can be distributed at large scale without medical follow-up, e.g., RUSFs, FBF, or other non-RUTF variations of LNS which are commonly used for the management of MAM. FBFs in turn are divided into CSB, an outdated FBF, enhanced FBFs which compared to CSB have additional micronutrients or milk and are commercially available, and locally produced FBFs (i.e., not commercially available, produced at the point of care) (see Figure 2). Non-specially formulated foods are foods that are typically consumed by society-at-large without any specific design or manufacturing for the purpose of addressing undernutrition. These “local foods” or “home foods” can include locally grown foods, imported foods, or foods provided by humanitarian or development organisations in response to food shortages, e.g., food baskets in a general food distribution.

### 2.2. Search Methods for Identification of Studies

The search strategy was formulated using the PICO format as described in Table 1 and the following databases were searched: MEDLINE, EMBASE, Web of Science Index Medicus, CINAHL, Lilacs, CENTRAL (Cochrane Library) and eLENA (WHO), Index Medicus for the WHO Eastern Mediterranean Region, and African Index Medicus. Key search terms included: “Infant”, “Child”, “Malnutrition”, “Protein-Energy Malnutrition”, “Wasting”, “Acute Malnutrition”, “Undernutrition”, “Weight-for-Height”, “Mid-Upper Arm Circumference”, “Food”, “Infant Food”, “Food, Fortified”, “Food, Fomulated” “Dietary Supplements”, “Ready-to-Use Therapeutic Food”, “Ready-to Use Supplementary Food”, “Ready-to-Use Food”, “Corn-Soy Blend”, “Wheat-Soy Blend”, “Lipid-Based Nutrient Supplement*”, “Nutributter”, “Supercereal”, “Community-Based Management”, “Integrated Community Case Management”, “Integrated Management of Acute Malnutrition” and “Supplementary Feeding Program*”. The full search strategy can be found in Supplementary Table S1. No language or date restrictions were applied to the search. The date of the final search was the 23rd of August 2021. The bibliography of all included studies and relevant systematic reviews was hand searched to identify missing papers.

### 2.3. Selection of Studies

Search results were exported into EndNote, de-duplicated, and uploaded into Covidence, a web-based systematic review software, for screening [19]. Two review authors independently assessed papers for relevance at both the title and abstracts and full-text screening stage. Any discrepancies were resolved by discussion or contacting a third author. Reasons for exclusion were recorded for studies excluded at the full-text screening stage.

### 2.4. Data Extraction

The following data were extracted from included studies and entered into an excel spreadsheet: author, journal, publication year, location, data collection dates, total duration of study, study setting, context (conflict, humanitarian or stable setting, geographical location, seasonality, food security/insecurity, urban/rural), study design, participant characteristics (number, age, gender, inclusion criteria, exclusion criteria, HIV status, number of participants per group, anthropometric characteristics), description of intervention and comparator (provider, type, dose and duration), outcomes of interest, funding source and sponsorship, study limitations and notable conflicts of interest. Two review authors independently extracted data from the papers. Any discrepancies were resolved by discussion or contacting a third author.

### 2.5. Quality Assessment of Included Studies

Quality assessments were carried out using the updated Cochrane risks of bias tool for randomised trials [20]. Two independent authors assessed the quality of all eligible studies and disagreements were resolved by consensus or by contacting a third author.

### 2.6. Data Analysis

After data extraction, one review author transferred data into Review Manager (RevMan) 5.4 software [21]. Data were analysed separately for the three different sub-questions and comparisons as described above. For dichotomous outcomes, results were presented as risk ratios (RRs), whereas for continuous outcomes they were presented as mean difference (MD) along with a 95% confidence interval (CI). Meta-analyses were conducted only where they were considered to be practically relevant, i.e., if the intervention and food composition, participants, and the underlying clinical question were sufficiently similar for pooling. Random effects analysis was performed for all comparisons, using inverse variance and Mantel–Haenszel methods to calculate the weights for continuous and categorical outcomes. This is a more conservative approach, as data were heterogeneous. Our search did not identify any non-randomised studies. Both individually randomised trials and cluster-randomised trials were included in the analyses. All but one study, Amegovu et al. [22], were adequately adjusted for clustering. The Amegovu et al. study was adjusted for clustering using an intraclass correlation coefficient of 0.01 as reported in the study by Nikiema et al. [23]. Where cluster-randomised controlled trials (RCTs) and individually randomised trials were similar in intervention and outcome assessment, the results from both were combined in one meta-analysis. Effect sizes and standard errors were meta-analysed using the generic inverse-variance method using RevMan software. Statistical heterogeneity was assessed using τ^2^, I^2^, and the significance of the χ^2^ test. Furthermore, heterogeneity was assessed by visually inspecting forest plots. The creation of funnel plots to explore possible small studies and publication biases was considered but not possible due to the small number of studies. Random-effects meta-regression was performed using the metareg macro command in Stata 17 to investigate the relationship between dose and duration on anthropometric outcomes. Continuous predictors included supplement dose (kcal per day) and a fixed duration of supplementation (weeks), respectively. These meta-regression analyses were weighted by the inverse of the study group-level variance for the respective outcomes, including anthropometric recovery and MUAC gain. The analyses were fitted with restricted maximum likelihood. I^2^ values represent the residual variation between groups. Sensitivity analyses were conducted on the primary outcomes by excluding studies which had an unclear risk of bias for both sequence generation and allocation concealment.

### 2.7. Sub-Group Analysis

The following subgroup analyses were considered: study context (conflict, humanitarian or stable setting, geographical location, seasonality, food security/insecurity, urban, rural), age group of children (6–23 months, 24–59 months, and >5 years up to 18 years), duration of the intervention (weeks), dosage and duration of the specially formulated food (kcal), facility-based vs. community-based approaches, HIV status, children with or without co-morbidities, breastfeeding status, concurrent stunting and household socio-economic status. Subgroup analysis for most of the comparisons and outcomes was not possible due to the small number of studies in each group. Subgroup analysis by age was not possible due to overlapping age ranges. For comparisons 1a (enhanced FBFs vs. LNS), 1b (CSB vs. LNS), and 2b.2 (RUSF without animal protein vs. RUSF with animal protein) subgroup analysis for dose and duration were carried out; for comparison 2.a1 (Enhanced FBFs vs. locally produced FBFs) subgroup analysis by dose was carried out. For the purpose of subgroup analysis by dose the following dosage groups were created: 1. Fixed-dose (low) which included studies providing a fixed dose of supplements of <500 kcal/day; 2. Fixed-dose (medium) which included studies providing 500 up to 1000 kcal/day; 3. Fixed-dose (high) included studies which provided supplements of 1000 kcal/day or more; 4. Weight-dependent dose (low) which referred to a weight-dependent dose of 40 kcal/kg/day; 5. Weight-dependent dose (high) which referred to a weight-dependent dose of 75 kcal/kg/day.

Evidence profiles were constructed for outcomes of interest summarising the quality of evidence as per the Grading of Recommendations, Assessment, Development, and Evaluation (GRADE) criteria where these were relevant [24]. This covers consideration of the within-study risk of bias, directness of evidence, heterogeneity, the precision of effect estimates and risk of publication bias. We rated the certainty of the evidence for each key outcome as “high”, “moderate”, “low”, or “very low”. We followed the Preferred Reporting Items for Systematic reviews and Meta-Analyses (PRISMA) guidelines for reporting. The PRISMA checklist can be found in the Supporting Information under Annex 1. The protocol for this review was registered with the International Prospective Register of Systematic Reviews (PROSPERO: CRD42021273432).

## 3. Results

### 3.1. Results of the Search

**Figure 3:**
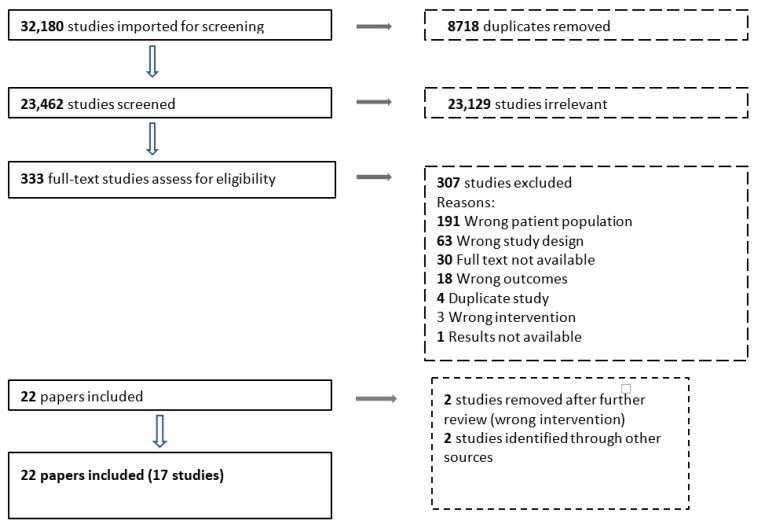
PRISMA flow diagram.

Our search identified a total of 32,180 records for screening. After the removal of 8718 duplicates, 23,462 records were screened based on titles and abstracts. A total of 333 full texts were reviewed and we included 20 papers from 15 studies [10,13,14,15,23,25,26,27,28,29,30,31,32,33,34,35,36,37,38,39]. An additional two studies were later identified through other sources [22,40]. The study by Amegovu et al. [22] was found in the reference list of one of the previously published systematic reviews [41]. The protocol for the Nane et al. study was picked up by the search [40] and so results were included even though they were only published two months after the search. The reasons for exclusions are shown in Figure 3. Out of the 30 studies where abstracts and/or full texts could not be identified, 25 were published between 1963 and 1996 and given their date of implementation; these studies were unlikely to include the correct patient population and outcomes required for inclusion in this analysis. The remaining five studies without full-text review were published between 2001 and 2018; based on the title and abstract, these were unlikely to be relevant yet this could not be confirmed by full text review.

### 3.2. Characteristics of Included Studies

A total of 22 papers from 17 studies, including 23,005 participants were included in this review [10,13,14,15,22,23,25,26,27,28,29,30,31,32,33,34,35,36,37,38,39,40] (Table 2). The majority of studies were conducted in Africa (*n* = 16) and one in Asia. Studies were published between 2009 and 2021 and all were RCTs. Seven were cluster randomised trials [23,25,26,31,32,33], whereas the others were individually randomised trials [10,13,14,15,29,34,36,37,38,40]. All trials enrolled children between six months of age and five years, but the age ranges differed between studies: 11 studies included participants aged 6 to 59 or 60 months [10,14,15,22,26,31,33,34,36,38,40], one study included participants 25–59 months [37], two included participants 6 to 23 or 24 months [13,23], one with 6–35 months [25], one study included participants aged 6–18 months [32], and one with 12–18 months [29]. The dose of supplementary foods ranged from 204 kcal/day to more than 1400 kcal/day and foods were given either in a fixed or weight-dependent dose. The duration of treatment also varied, with five studies treating children for a fixed period of 12 weeks [13,25,29,31,40], and one study for a fixed duration of 8 weeks [37]. The remaining five studies treated children until recovery with a maximum treatment duration of 8 weeks [36], 12 weeks [14,22,23], 16 weeks [10,33] or 17 weeks [26]. More details on the interventions for each study are shown in Table 2. Among the randomised studies, four were judged to have a low risk of bias [15,36,38,40], nine were judged to have some concerns [14,22,23,26,29,30,32,34,37] and four studies were judged to have a high risk of bias [10,25,31,42]. Risk of bias graphs providing further detail can be found in Supplementary Figure S1.

### 3.3. Effect of Intervention

A total of 17 studies (ten RCTs, seven cRCTs) including 23,005 participants were meta-analysed. All identified studies fit in subquestions 1 and 2, and no studies matching the criteria for subquestion 3 were identified. Of these 17 studies, 9 compared FBFs vs. LNSs including 13,926 participants, 7 studies compared two different LNS products including 12,347 participants; and 5 studies compared different FBF products, which included 4942 participants. The number of studies per comparison group is shown in Figure 2. Effectiveness results are presented by comparison group below.

#### 3.3.1. Sub-Question 1: Effectiveness of Fortified Blended Foods (FBFs) Versus Lipid-Based Nutrient Supplements (LNS)

***Comparison 1.a. Enhanced FBFs compared to LNS***

**Figure 4:**
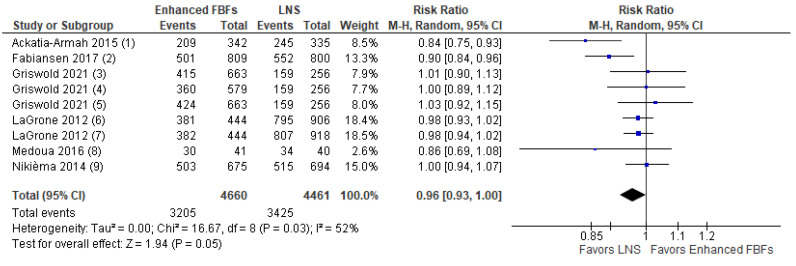
Forest plot of enhanced FBF compared to LNS—Outcome: Recovery rate (1) CSB++ Supplementary Plumpy/Nutriset; Recovery defined as WLZ > −2.0 and MUAC >12.5 cm for >= 2 follow-up visits; (2) Enhanced FBF vs. LNS, factorial trial including 12 different products; Recovery at 12 weeks (WHZ >= −2 and MUAC >= 125 mm); Supercereal Plus with amylase (SC + A) vs. RUSF; Recovery defined as achieving MUAC ≥12.5 cm by the seventh visit and no bipedal oedema; (3) Corn–soy–whey blend (CSWB) w/oil vs. RUSF; Recovery defined as achieving MUAC ≥12.5 cm by the seventh visit (12 weeks) and no bipedal oedema; (4) CSB+ w/oil vs. RUSF; Recovery defined as achieving MUAC ≥12.5 cm by the seventh visit (12 weeks) and no bipedal oedema; (5) CSB++ vs. soy RUSF; Recovery defined as reaching a WHZ >= −2; (6) CSB++ vs. soy/whey RUSF; Recovery defined as reaching a WHZ >= −2; (7) CSB+ with soy oil vs. RUSF; Recovery defined as reaching a WHZ >= −2; (8) CSB++ vs. locally produced RUSF; Recovery defined as reaching a WHZ >= −2.

A total of six studies were found that compared enhanced FBFs to LNS [14,23,25,30,31,37]. As defined above, enhanced FBFs are those that compared to the standard or outdated CSB contain additional micronutrients and/or milk. These included commercially available and/or imported products such as CSB+ and CSB++. Findings suggest that there may be little or no difference between enhanced FBF and LNS in terms of recovery (RR: 0.96, 95% CI: 0.93 to 1.00; 6 studies, *n* = 9121; low certainty evidence, Figure 4) and deterioration to severe wasting (RR: 1.03; 95% CI: 0.86 to 1.23; *n* = 7699; five studies; moderate certainty evidence). Enhanced FBFs compared to LNS probably lead to a slightly lower WHZ gain (MD: −0.09 z-scores; 95% CI: −0.14 to −0.04; moderate certainty evidence). Enhanced FBFs compared to LNS may lead to a lower MUAC gain (MD: −0.26 cm; 95% CI: −0.48 to −0.03; *n* = 3470, three studies, low certainty evidence), weight gain (MD: −0.36 g/kg/day; 95% CI: −0.56 to −0.15; *n* = 3470, three studies, low certainty evidence) and probably do not show a difference on height gain (MD: −0.02 cm; 95% CI: −0.2 to 0.15; *n* = 3389, two studies, moderate certainty evidence). Enhanced FBFs compared to LNS may show little or no difference in terms of sustained recovery (RR: 0.99; 95% CI: 0.87 to 1.11; *n* = 1967, one study, low certainty evidence) and non-response (RR: 1.1; 95% CI: 0.81 to 1.5; *n* = 6448, five studies, low certainty evidence). Finally, enhanced FBFs compared to LNS may only have a small or no effect on relapse (RR: 0.99; 95% CI: 0.81 to 1.22; *n* = 1967; one study, low certainty evidence). The evidence with regards to time to recovery is very uncertain (MD: 3.2 weeks; 95% CI: −0.06 to 6.45, *n* = 4265; four studies, very low certainty evidence)**.** Data for the effect of enhanced FBFs compared to LNS on HAZ and WAZ were not available. More details on the certainty of evidence judgment can be found in Supplementary Table S2 and forest plots for outcomes other than recovery rates under this comparison are presented in Supplementary Figures S2–S10.

Subgroup analysis by dose in the comparison of enhanced FBF compared to LNS did not change the direction of effect for recovery rate, deterioration to severe wasting, WHZ, MUAC, weight, and height gain as well as non-response but precision was reduced. Although the time to recovery was longer with enhanced FBFs in studies providing a medium fixed dose or weight-dependent doses compared to LNS, as in one study providing a low fixed dose enhanced FBFs reduced time to recovery compared to LNS (Supplementary Figures S10–S18). Similarly, subgroup analysis by duration (8 vs. 12 weeks) did not change the direction of effect for outcomes measured (Supplementary Figures S19–S25).

One study, Trehan et al. (2015), compared post-discharge outcomes in children treated for a fixed 12-week period compared to children treated until recovery and found that children treated for the fixed period were more likely to remain well nourished (71% vs. 63%, *p* = 0.0015) and less likely to die (2% vs. 4%, *p* = 0.082) throughout a 12-month follow-up period. However, regression modelling showed that MUAC and WHZ at the end of supplementary feeding were the most important factors in predicting which children remained well-nourished, not length of treatment (*p* < 0.001) [39].

Sensitivity analysis was conducted for studies with unclear risk of bias for both sequence generation and allocation concealment. Only one study was identified that fit these criteria [31] and removing it did not lead to a significant change in the recovery rate and deterioration to severe wasting.

***Comparison 1.b. CSB compared to LNS***

**Figure 5:**
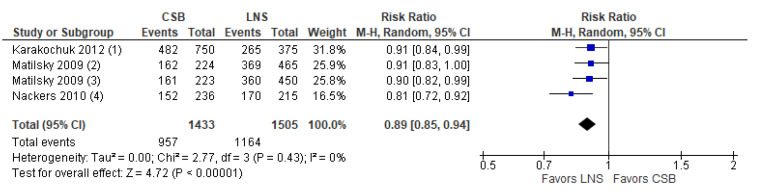
Forest plot of CSB compared to LNS—Outcome: Recovery rate (1) CSB vs. Supplementary Plumpy/Nutriset; Recovery was defined as WFH ≥ 85% on 2 consecutive visits within 16 weeks; (2) CSB vs. milk/peanut fortified spread; defined as having a WHZ > −2 within 8 weeks; (3) CSB vs. soy/peanut fortified spread; defined as having a WHZ > −2 within 8 weeks; (4) CSB vs. standard RUTF; Recovery defined as reaching a WHM% >/= 85% for 2 consecutive weeks within 16 weeks.

A total of three studies were identified that compared CSB to LNS. CSB is a type of FBF that does not contain milk and has lower concentrations of some micronutrients than enhanced FBFs. It predates the enhanced FBFs and is not as commonly used as it was in the past. Findings suggest that CSB compared to LNS may result in lower recovery rates (RR: 0.89; 95% CI: 0.85 to 0.94; *n* = 2938; three studies; low certainty evidence; Figure 5) and probably lower end of intervention WHZ (MD: −0.15; 95% CI: −0.25 to −0.05; one study; moderate certainty evidence)**.** Compared to LNS, CSB probably results in lower weight gain in the first two weeks of treatment (MD: 1.86 g/kg/day; 95% CI: −2.67 to −1.05; *n* = 312, one study, moderate certainty evidence), and may not have an effect on deterioration to severe wasting (RR: 1.15; 95% CI: 0.73 to 1.84; *n* = 2938; three studies; low certainty evidence), time to recovery (MD: 0.7 weeks; 95% CI: −1.6 to 3.00, *n* = 322; one study, low certainty evidence) or relapse (RR: 1.12; 95% CI 0.73 to 1.72; *n* = 322; one study, very low certainty evidence). Furthermore, there was little or no effect of CSB on HAZ (MD: −0.15; 95% CI: −0.35 to 0.06, *n* = 1362, one study, moderate certainty evidence) and MUAC gain (MD: −0.05 mm/day; 95% CI: −0.11 to 0.01; *n* = 312, 1 study, moderate certainty evidence) compared to LNS. The evidence for non-response was very uncertain (RR: 1.27; 95% CI: 0.68 to 1.36; *n* = 2938, three studies, very low certainty evidence. Data were not available for WAZ, height gain and sustained recovery. More details on the certainty of evidence judgment can be found in Supplementary Table S3 and forest plots for outcomes other than recovery rates are presented in Supplementary Figures S26–S33.

Subgroup analysis by dose and duration did not lead to any changes in conclusions. Results are presented in supporting figures Supplementary Figure S34–S39).

***Comparison 1.c. Locally produced FBFs compared to LNS***

**Figure 6:**
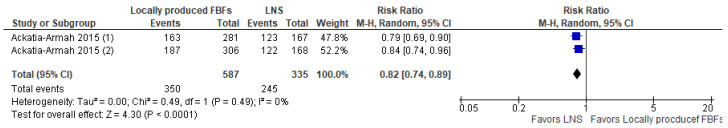
Forest plot of locally produced FBF compared to LNS—Outcome: Recovery rate (1) Locally milled flours (LMF) compared to RUSF; Recovery defined as WLZ > −2.0 and MUAC >12.5 cm for >= 2 follow-up visits; (2) Misola compared to RUSF; Recovery defined as WLZ > −2.0 and MUAC >12.5 cm for >= 2 follow-up visits.

Comparison 1.c. included a comparison between locally produced FBFs, defined as any blended cereals containing added micronutrients that were not commercially produced or imported, and LNS which in this case was RUSF. Only one study compared locally produced FBFs to LNS [25]. In this case, the locally produced products did not include milk and had lower quantities fats and of some micronutrients (such as Vitamins B, D, E, and K) compared to RUSF or enhanced FBFs. Findings suggest that the use of these locally produced FBFs compared to LNSs may result in lower recovery rates (RR: 0.82; 95% CI: 0.74; 0.89; *n*= 922; one study; low certainty evidence; Figure 6), lower WHZ gain (MD: −0.34 z-score; 95% CI: −0.48; −0.2; *n*= 922; one study; low certainty evidence) and lower gain in MUAC (MD: −0.17 cm; 95% CI: −0.04 to −0.31; *n*= 922; one study; low certainty evidence). Furthermore, the locally produced FBFs probably result in lower weight gain (MD: −0.29 kg; 95% CI: −0.39 to −0.19; *n* = 922; one study; moderate certainty evidence) as well as height gain (MD: −0.26 cm; 95% CI: −0.45 to −0.06; *n* = 922; one study; moderate certainty evidence) and may result in increased non-response (RR: 1.16, 95% CI: 1.29 to 2.1; *n* = 922; one study; low certainty evidence) and time to recovery (MD: 3.4 weeks; 95% CI: 2.22 to 4.58; *n* = 922; one study; low certainty evidence) when compared to LNS. The effects on sustained recovery, WAZ, HAZ, and relapse were not reported. More details on the certainty of evidence judgment can be found in Supplementary Table S4 and forest plots for outcomes other than recovery rates under this comparison are presented in Supplementary Figures S40–S45.

#### 3.3.2. Sub-Question 2: Effectiveness of Different Compositions, Doses or Durations among Different Variations of the Same Type of specially Formulated Food (FBFs or LNSs)

***Comparison 2.a. Comparisons of FBFs***
***Comparison 2.a.1. Enhanced FBFs compared to locally produced FBFs***

**Figure 7:**
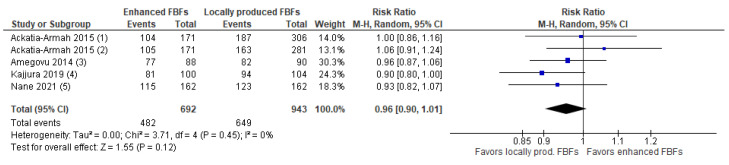
Forest plot of enhanced FBF compared to locally produced FBFs—Outcome: Recovery (1) CSB++ compared to Misola; Recovery defined as WLZ > −2.0 and MUAC > 12.5 cm for >= 2 follow-up visits; (2) CSB++ compared to locally milled flours (LMF); Recovery defined as WLZ > −2.0 and MUAC > 12.5 cm for >= 2 follow-up visits; (3) CSB+ compared to locally produced soy peanut blend (SBP); Recovery defined as WHZ > −2 and attaining 10% of their admission weight for two consecutive visits; (4) CSB+ compared to malted-sorghum-based porridge (MSBP); Recovery defined as WHZ > −2; (5) CSB+ vs. local ingredients based supplements (LIBS); Recovery was defined as MUAC > 12.5 cm and/or WHZ > −2 without bipedal oedema at the end of 12 weeks.

Four studies were found comparing enhanced FBFs to locally produced FBFs, where enhanced FBFs are defined as products that are imported and/or commercially available and, compared to the traditional CSBs, they contain milk and/or have an improved micronutrient composition. Locally produced FBFs are not commercially available and, in the studies included here, do not contain milk powder as a source of protein [22,25,32,40]. Findings suggest that enhanced FBFs compared to locally produced FBFs may have little or no effect on recovery (RR: 0.96; 95% CI: 0.9; 1.01; *n* = 1635; four studies; low certainty evidence; Figure 7). Enhanced FBFs compared to locally produced FBFs may show no difference in deterioration to severe wasting (RR: 2.0; 95% CI: 0.37; 10.77; *n* = 324; one study; low certainty evidence). In one study children receiving enhanced FBFs had a lower WAZ at the end line compared to those who received locally produced FBFs (MD: −0.27 z-scores; 95% CI: −0.48; −0.07; *n* = 204; one study; low certainty evidence). Enhanced FBFs probably result in a slightly higher weight gain (MD: 0.13 kgs; 95% CI: 0.05; 0.21; *n* = 1457; three studies; moderate certainty evidence), little to no difference in height gain (MD: 0.05 kgs; 95% CI: −0.19; 0.29; *n* = 1457; three studies; low certainty evidence), or non-response (RR: 0.91; 95% CI: 0.76 to 1.1; *n* = 1107; two studies; low certainty evidence). The evidence with regards to time to recovery (MD: 9.98; 95% CI: 21.93 to 1.96; *n* = 881; two studies; very low certainty evidence), HAZ (MD: −0.17 z-scores; 95% CI: −0.4; −0.07; *n* = 382; two studies; very low certainty evidence), WHZ (MD: −0.04 z-scores; 95% CI: −0.29; 0.2; *n* = 1311; three studies; very low certainty evidence) and MUAC gain (MD: 0.06 cm; 95% CI: −0.13; 0.25; *n* = 1253; two studies; very low certainty evidence) is very uncertain. The effects on sustained recovery and relapse were not reported. More details on the certainty of evidence judgment can be found in Supplementary Table S5 and forest plots for outcomes other than recovery rates under this comparison are presented in Supplementary Figures S46–S54. Subgroup analysis by dose and duration did not lead to any changes in results.

***Comparison 2.a.2. Enhanced FBFs without milk vs. enhanced FBFs with milk***

One study [31] compared enhanced FBFs without milk (CSB+ with oil) with enhanced FBFs with milk (in the form of CSWB or supercereal+ with Amylase) and found no difference in terms of their effect on recovery and deterioration to severe wasting (Supplementary Figure S55).

***Comparison 2.b. Comparisons of different variations of LNSs***
***Comparison 2.b.1. RUTF vs. RUSF***

Only one study was found comparing RUTF, an LNS designed for the treatment of SAM to RUSF, an LNS designed for the treatment of MAM [26]. RUTF when compared to RUSF showed little or no difference in recovery (RR: 1.02; 95% CI: 0.98 to 1.05; *n* = 1903; one study; moderate certainty evidence, Supplementary Figure S56). It probably has little or no effect on MUAC gain (MD: 0.1; 95% CI: 0.21 to 0.1; *n* = 643; one study; moderate certainty evidence). RUTF when compared to RUSF may lead to an increase in weight gain (MD: 0.2 g/kg/day; 95% CI: 0.08 to 0.32; *n* = 643; one study; low certainty evidence) but may show no difference in height gain (MD: 0.6; 95% CI: 1.54 to 0.34; *n* = 643; one study; low certainty evidence) and probably does not have an effect on relapse (RR: 0.79; 95% CI: 0.48 to 1.3; *n* = 536; one study; moderate certainty evidence). Under this comparison, data were not available for the outcomes of WHZ, WAZ, HAZ, deterioration to severe wasting, sustained recovery, and non-response. The evidence profile for this comparison can be found in Supplementary Table S6 and forest plots are presented in Supplementary Figures S57–S59.

***Comparison 2.b.2. RUSF without animal-based protein compared to RUSF with animal-based protein***

A total of four studies compared RUSF with animal-based protein compared to RUSF without animal-based protein [14,15,29,36]. In a meta-analysis, there were no significant differences between the two foods on recovery, deterioration to severe wasting, WHZ, WAZ, HAZ, MUAC, and height gain. However, when compared with no animal-based protein RUSF, RUSF with animal-based protein led to a small increase in weight gain (MD: −0.17; 95% CI: −0.32 to −0.03, two studies, *n* = 4054) and greater sustained recovery (RR: 0.88; 95% CI: 0.81–0.96, one study, *n* = 829). Furthermore, there was no difference in effect on non-response and time to recovery. Finally, animal-based vs. non-animal-based protein in RUSF led to lower levels of relapse (RR: 1.24; 95% CI: 1.04 to 1.49, one study, *n* = 351). Forest plots for the comparison are presented in Supplementary Figures S60–S71. Subgroup analysis by dose and duration did not lead to any changes in results.

***Comparison 2.b.3. RUSF vs. alternative (high protein) RUSF***

One study compared RUSF to a protein-optimised RUSF, where the latter had a calculated digestible indispensable amino acid score (DIAAS) of 95%, compared to 63% in the control RUSF [38]. Compared to the control RUSF, the protein-optimised RUSF did not lead to an increased recovery rate (88% vs. 87%, *p* = 0.6, RR 1.01; 95% CI: 0.98 to 1.05). Furthermore, there were no differences in deterioration to SAM, MUAC gain, weight gain, non-response, and time to recovery. Other outcomes, i.e., WHZ, WAZ, HAZ, height gain, and sustained recovery were not reported in this study. Forest plots for this comparison are shown in Supplementary Figures S72 and S73.

***Comparison 2.b.4. RUTF vs. alternative RUTF***

One study was identified that compared a standard RUTF to an alternative RUTF, where half the amount of peanut was replaced with local soybean and sorghum flour and 50% of protein from dairy came from a combination of whey protein concentrate and non-fat dried milk, rather than non-fat dried milk only [34]. Children receiving the standard RUTF were more likely to recover than children receiving the alternative RUTF (93.4% vs. 87.1%, *p* < 0.003, R: 0.93; 95% CI: 0.89 to 0.97; *n* = 869) and there was no difference in other reported outcomes of weight gain and MUAC gain. Forest plots for this comparison are shown in Supplementary Figures S74 and S75.

#### 3.3.3. Sub-Question 3. Effectiveness of Specially Formulated Foods Versus Non-Specially Formulated Food

No studies were found that fit into this sub-question.

#### 3.3.4. Dose and Duration of Specially Formulated Food

There were 19 observations included in the meta-regression of the dose of specially formulated food as a predictor of anthropometric recovery, which showed no relationship (β: 0.03%; 95% CI: −0.03, 0.09, *p* = 0.3; I2 = 0%) (Supplementary Figure S76). There were eight observations in the meta-regression of LNS dose as a predictor of MUAC gain which again showed no relationship (β: 0.00018 mm per day; 95% CI: −0.000051, 0.00042; *p* = 0.1; I^2^ = 0.00%) (Supplementary Figure S77). There were 16 observations included in the meta-regression of a fixed duration of treatment with LNS as a predictor of anthropometric recovery. There were two different fixed durations evaluated across studies, including eight weeks for one study and twelve weeks for the remainder of the studies in the meta-regression. These results indicated no relationship (β: −1.05%; 95% CI: −4.85, 2.74; *p* = 0.6; I^2^ = 29.42%) (Supplementary Figure S78).

## 4. Discussion

### 4.1. Summary of Results

This systematic review summarises evidence from 17 studies, all of which were randomised trials. Nine studies compared the effectiveness of LNS to FBF in MAM treatment. Generally speaking, when comparing LNS to FBF, differences in outcomes seem to lessen with enhancements in FBF composition. Although LNS compared to locally produced FBFs or CSB tended to lead to better recovery rates and other anthropometric outcomes, LNS compared to enhanced FBF showed no difference with regards to recovery, gains in height, WAZ, HAZ, deterioration to severe wasting, sustained recovery, non-response and relapse. However, LNS may result in greater WHZ gain, MUAC gain, and weight gain than enhanced FBFs. Sub-analyses based on different dosing and durations of supplements resulted in similar outcomes as the main analysis.

Seven studies compared two different LNS products. Results show that differences in protein type and quality in LNS products do not have a large impact on treatment outcomes for MAM children. One study compared RUTF to RUSF and found no effect on treatment outcomes. All studies comparing RUSF without animal-based protein to RUSF with animal-based protein, or LNS with higher versus lower quality protein generally found little to no differences in treatment outcomes. This can only be said for the current evidence base. It is possible that studies including new LNS formulations and compositions may produce different results.

Only five studies included in this review compared different compositions of FBF. Although overall there may not be a large difference between FBFs of different compositions in meta-analysis in outcomes such as recovery and deterioration to SAM, results differed for different outcomes and products which varied widely in ingredients and composition. It is worth pointing out also that with the exception of weight gain, the certainty of evidence under this comparison was either low or very low.

### 4.2. Overall Completeness and Applicability

The evidence base regarding different types and compositions of supplementary foods in MAM treatment is supported by 17 studies, 16 from Africa and 1 in Asia, which may limit the applicability of results in the Asian context.

Although this review was able to address the question of the type and composition of supplements, not much can be said about the optimal dose and duration. Subgroup analysis by dose did not lead to major changes in results. This is partly because the studies that compared different supplements were isocaloric, with the exception of two studies [10,33]. Therefore, future studies comparing the same supplement with different dosage regimens would be better placed to answer this question. Although a number of studies testing different dosages in the management of SAM exist, we did not identify any for the management of MAM. With regard to the question of duration, studies provided treatment for different lengths of time: either for 8, 12 or 17 weeks, with some of these treating children for a fixed period of time, whereas others provided treatment until recovery within a maximum timeframe. Subgroup analysis by duration did not lead to any changes in conclusions. One study, Trehan et al., compared post-discharge outcomes in children treated for a fixed period versus until recovery, and found that children treated for the fixed period were more likely to remain well-nourished [39]. However, the authors point out that, when comparing children with MAM who were treated for a fixed duration of time compared to an anthropometric goal, logistic regression modelling identified greater MUAC and WHZ as the most important variables that predicted which children remained well-nourished during a post-discharge follow-up period. These results are consistent with other post-discharge follow-up studies identifying enhanced anthropometric indicators at discharge as the strongest predictors of sustained recovery from MAM [15,28]. We carried out a metaregression to further investigate the relationship between dose and duration and anthropometric outcomes but the results were inconclusive. Meta-regressions were only possible with two outcomes for dosing and one outcome for duration, but no relationship was found. Datapoints were too few to carry out meta-regressions for other outcomes. There was wide variability in recovery rates within and across studies with the same fixed duration partly linked to different study contexts. Given only two fixed durations were evaluated, it is difficult to draw conclusions from this analysis.

Lastly, no studies were identified that compared specially formulated foods to home foods, leaving a gap in the evidence.

### 4.3. Agreements and Disagreements with Other Studies or Reviews

Overall, results from this review largely align with those of previous reviews. Older systematic reviews found LNS to be more effective than FBF in terms of increasing recovery rates, improving anthropometrics, and lowering the risk of deterioration into SAM [17,43,44]. This aligns with our results because most of the studies included in these older reviews consisted of comparisons between LNS and an “outdated” CSB formulation. Our results for the comparisons of LNS vs. an “outdated” CSB are consistent with theirs, but we also conducted an additional analysis that included more recent studies comparing LNS with enhanced FBF, which ultimately demonstrated little or no difference in recovery rates. A 2021 review by Gluning et al. [41] identified 13 studies with results also indicating that children treated with LNS as opposed to FBF have an increased probability of recovery and a lower risk of persistent MAM. However, the studies included in the analysis differed slightly, e.g., while they excluded studies with the “outdated” CSB, they were not able to include the most recent study by Griswold et al. (2021) which found no differences between enhanced FBFs vs. LNS [31]. Results also showed that the use of specially formulated foods was associated with a higher probability of recovery, a lower risk of progressing to SAM and a lower risk of defaulting compared with children who did not receive supplementation [41]. Another recent systematic review by Das et al. (2020) identified 14 studies that compared RUSF to other foods in the treatment of moderate wasting. Their results found that when comparing different compositions of LNS, including standard RUSF with whey RUSF, the recovery rate, time to recovery, and weight gain are similar, but MUAC gain may be improved by standard RUSF. When compared to CSB, this review found that standard RUSF may improve recovery, weight gain, and MUAC gain, though time to recovery is similar [45].

Although our review found little or no difference between LNS with and without animal protein it is worth mentioning that these results do not necessarily coincide with the body of evidence related to the type and quality of protein in products used for treating SAM children. Studies have shown that when treating SAM children, lower protein quality in RUTF may have a significant impact on outcomes [46]. Variability may be due to different vulnerabilities between SAM and MAM children, or possibly the difference in the proportion of the diet covered by the specially formulated foods. Small changes to the type and composition of foods provided to MAM children as supplementary to home foods may not have as much impact as changes to RUTF composition which is provided in higher quantity and proportion of the SAM child’s diet.

### 4.4. Implications for Practice

Results from this review imply that LNS may be preferable compared to the “outdated” CSB and less enhanced FBF. However, given the small differences in treatment outcomes between enhanced FBF and LNS as well as the different compositions of the same LNS or FBF supplements, any programmatic decisions on which of these to choose may therefore want to consider acceptability, availability, as well as cost and cost-effectiveness.

One of the studies included in this review had an accompanying acceptability study which found that carers and children preferred LNS compared to FBF, and products with higher milk content compared to products with soy [30,47]. However, acceptability likely differs across contexts. Although several studies commented on the cost of supplements, information on the cost-effectiveness of MAM programmes is limited. Where cost has been assessed, the costs of FBF were typically cheaper than LNS [14,25,36,37], but adding milk as an ingredient increases costs [14,15,36]. It is important to note however, that supplementary food costs (FBFs or LNS) costs are only part of the overall programme costs and differences in supplement costs may be offset by other factors affecting overall programme costs per child, such as time to recovery, transport costs, storage costs and household opportunity costs. Griswold et al. (2021) found that supplements accounted for 7%–14% of total programme cost [31]. In this study, although the food product cost in the CSB+ w/oil and CSWB w/oil groups was lower at US$6.46 and US$7.34, respectively, compared to US$12.94 in the SC + A and US$12.18 in the RUSF group, the total cost per enrolled child was similar across the four groups with 90.1 in the CSB+/oil, 90.6 in the CSWB w/oil group, 93.6 in the SC + A group, and 93.1 in the RUSF group. Furthermore, costs per child who recovered and per child who sustained recovery were similar across groups [31]. A cost-effectiveness study by Isanaka et al. (2019), linked to the Ackatiah-Armah et al. (2015) trial included here, found MAM treatment to be cost-effective in general and that despite having the highest per-unit food costs, treatment with RUSF was more cost-effective compared to other supplements (CSB++, Misola and locally milled flour) [48]. The proportion of supplement costs in this study were higher than in the Griswold et al. (2021) study and ranged from 28% to 45%. Differences between these two studies are likely influenced by methodological decisions with regards to which costs are included in the total.

The question of which supplement to provide to MAM children is relevant also in the context of currently debated innovations and simplifications to the public health models designed to manage acute malnutrition. Such innovations include a range of modifications aimed to improve coverage and reduce the cost of treatment, including delivery of services by CHWs, screening by family members at the household level, use of MUAC and oedema only for admission and discharge criteria, combining treatment of SAM and MAM (possibly using RUTF only), and modifying standard dosing regimens. Based on the evidence in this review, there is no data to suggest that RUTFs would result in poor outcomes when used for MAM treatment. Body composition and cost are often cited as a concern here. However, studies investigating the impact of different types of supplements on body composition, either comparing FBF to LNS, [13,49] or RUSF versus RUTF [35] did not identify any adverse effects on body composition. In terms of cost-effectiveness, Bailey et al. (2020) found that, compared to standard treatment, a simplified and combined protocol for the treatment of moderate and severe wasting providing the same product, RUTF, to both children with MAM and SAM was US$123 per child cheaper than the standard protocol, despite not being able to include any potential cost-saving linked to having only one supply chain. However, supply chain issues and varying product costs would also need to be considered. Moreover, an analysis of potential changing needs over time would be useful, as overall costs could be offset in the long term by treating children earlier, thus preventing SAM and reducing the amount of RUTF needed for SAM treatment.

### 4.5. Implications for Research

Our systematic review was limited to anthropometry-based outcomes and time to recovery. When making decisions about which type of supplement to use, other indicators of nutritional status such as body composition, micronutrient status and functional outcomes may also be useful to consider. Future studies should consider non-anthropometric outcomes that may glean more insights into the most appropriate type, composition, dose, and duration of supplementation. One study included in this review found that LNS compared to enhanced FBF led to more gain in lean mass irrespective of soy quality and milk content, reduced prevalence of anaemia and iron-deficiency anaemia by 16.9 and 10.5 percentage points, respectively, though overall prevalence of anaemia remained high at the end of supplementation [13,50]. Data from the same study also indicates that low levels of n-3 poly-unsaturated fatty acids and serum cobalamin in children with MAM are common and that increase in serum cobalamin during supplementation was inadequate [51,52]. Similarly, the study by Ackatiah-Armah et al. (2015) found that 90% of children were anaemic at enrolment and that haemoglobin status increased throughout the study period with the greatest improvements seen in the RUSF group, in comparison to the CSB++ and locally produced FBFs. Nevertheless, the final prevalence of anaemia remained very high: >81% in all groups [25].

The content of milk required in a supplement is still a key question in MAM programmes [53,54]. It is, however, also an expensive ingredient [55], and many studies have therefore investigated compositions with different levels of milk protein or substitutes for milk protein. Overall, studies included in this review which compared supplements of different amounts of milk did not find significant differences in recovery rates. However, milk may have other benefits as well. For example, one study found that while there were no differences in terms of recovery from MAM between 0%, 20%, and 50% milk protein; higher amounts of milk protein appeared to be beneficial for language and fine motor development [13,56]. Furthermore, as discussed above, more evidence is needed on the cost-effectiveness of MAM supplementation programmes, including considerations of supply chain costs and time to recovery, as well as the best dose and duration of supplementation.

Lastly, in order to further improve outcomes of MAM treatment programmes, it may also be worth investigating other patient, context, and programmatic factors which may have a greater impact on recovery than small changes in compositions of supplementary foods. As an example, in the study by LaGrone et al., (2012) recovery rates between the CSB++, soy RUSF and soy/whey RUSF differed by less than 2%. The authors carried out binary logistic regressions to identify factors associated with recovery and found that a number of factors such as season of enrolment, a child being able to stand without assistance, a child taking antibiotics at enrolment, child illness and HIV status were significantly associated with recovery while the type of supplement was not [14]. A 2013 non-randomised study by Purwestri et al. (2013) compared the provision of the same LNS supplement between two different frequency of distribution protocols, including on a daily versus weekly basis. In this study, outcomes were enhanced in the daily protocol with higher WHZ gain (*p* = 0.027) and recovery rate (*p* = 0.004). Authors cited compliance and admission in the daily programmes as significant factors for improving the likelihood of recovery [57]. Furthermore, one randomised controlled trial in Burundi piloting the introduction of malnutrition prevention and care indicators within its performance-based financing (PBF) scheme found that this had a positive impact on recovery rates from MAM, with 78% in the control group and 97% in the intervention group [58]. These differences in recovery rates are larger than those found in any of the trials included in this review comparing different compositions.

Lastly, there is a remarkable paucity of evidence in evidence from Asia: only one of the seventeen studies included in this review was conducted in Asia. Given that 70% of wasted children globally live in Asia [1], more evidence from Asian contexts is needed.

## 5. Conclusions

A wide range of product variability in the studies included poses challenges for combining and comparing results; nonetheless, the consistency and direction of effect across the varying studies does lead to general evidence-based conclusions. The use of LNS and enhanced FBF tend to produce similar results; however, LNS seems to perform better when compared to less-enhanced locally produced or imported FBF. When comparing various compositions of LNS there may not be large differences in terms of clinically relevant results. Therefore, when aiming to determine the programme and policy applications of these results, it is important to consider other factors such as product cost, cost-effectiveness, acceptability, program protocols, logistics, and supply chains. Future research is required to address the question of optimal dose and duration of supplementation.

## Figures and Tables

**Table 1:** PICOs criteria for inclusion of studies.

Parameter	Criteria
Population	Children aged > 6 months with moderate wasting, defined as a weight-for-height z-score of ≥−3 and <−2 and/or a mid-upper arm circumference of ≥ 11.5 cm and <12.5 cm or a weight-for-height between >70% and <80% of the median and no oedema, treated either as inpatients or outpatients.
Intervention	Dietary treatment in addition to standard clinical care.
Comparator	No specific dietary treatment in addition to standard clinical care and/or comparing different dietary treatment approaches to each other.
Outcomes	Anthropometric recovery, anthropometric outcomes (weight-for-length, weight-for-age, mid-upper arm circumference, weight and height gain), sustained recovery, deterioration to severe wasting, time to recovery, non-response, relapse.
Study design	Included: Randomized or non-randomized controlled trials. Excluded: Cross-sectional or observational studies, case reports, animal studies, case studies, opinions, editorials, commentaries, letters, conference abstracts, studies with external comparison groups and reviews.

**Table 2:** Characteristics of the included studies ^1^.

Study Number	Author, Year	Study Design and Setting	Participants, Admission and Recovery Criteria	Intervention/Control and Dose and Duration of Supplementation	Outcomes
1.	Ackatia-Armah et al., 2015 [25]	cRCT Twelve community health centres in a rural setting in Diola Health District, Bamako, Mali	1264 children aged 6–35 months ***Admission criteria:*** -WHZ <−2 and ≥−3 (WHO Growth Standards) or MUAC <12.5 cm and ≥11.5 cm -WHZ <80% and ≥70% of the NCHS median or MUAC <12.0 and ≥11.0 cm (National criteria in Mali at the time) ***Recovery*** from MAM was defined as WHZ >−2.0 and MUAC >12.5 cm during at least 2 consecutive follow-up visits	***Group 1:*** 500 kcal/day of ready-to-use supplementary food (RUSF) (n = 344) ***Group 2:*** 500 kcal/day of corn–soy blend (CSB++) n = 349) ***Group 3:*** 500 kcal/day of Misola (n = 307) ***Group 4:*** 500 kcal/day of locally milled flours + micronutrient powder (n = 284) ***Duration:*** 12 weeks	Adherence, MUAC, weight, length, WHZ, LAZ, haemoglobin, serum ferritin, retinol-binding protein, transferrin receptor, body iron stores, and plasma zinc
2.	Amegovu et al., 2014 [22]	cRCT Two health centres Kakamongole and Namalu in Nakapiripirit district, Karamoja, Uganda	440 children aged 6–59 months ***Admission criteria:*** -WHZ <−2 and ≥−3 (WHO Growth Standards) ***Recovery criteria:*** Children were defined as having recovered when they reached a WHZ> –2 and had attained 10% of their admission weight for two consecutive visits	***Group 1:*** 1200 kcal/day of corn–soy blend plus (CSB+) mixed with vegetable oil and sugar ***Group 2:*** 1228 kcal per day of sorghum peanut blend mixed with ghee and honey ***Duration:*** Up to 3 months	Recovery rate, time to recovery
3.	Bailey et al., 2020 [26] (Plus a linked nested cohort study (Lelijveld et al., 2021 [35]) and a secondary data analysis (Bailey et al., 2021 [27]))	cRCT Kenya and South Sudan (The nested cohort study reported only on the Kenya subsample)	4,110 Children aged 6–59 months, 2858 of these were MAM, the rest SAM. ***Admission criteria:*** MUAC < 12.5 cm and/or oedema (+/++, i.e., mild or moderate). MAM was defined as MUAC between 115 mm and 125 mm ***Recovery criteria:*** MUAC measurement of ≥12.5 cm for 2 consecutive visits	***Group 1:*** Standard protocol Children with MAM received 500 kcal/day of RUSF ***Group 2:*** Combined protocol children with MAM received 500 kcal RUTF/day (1 sachet/day). ***Duration:*** 17 weeks	Primary outcome: Nutritional recovery. Secondary outcomes: cost-effectiveness, coverage, defaulting, death, length of stay, average daily weight and MUAC gains, WHZ, WAZ, LAZ, and bioelectrical impedance analysis
4.	Chen et al., 2021 [29]	RCT Mirpur, Bangladesh	123 children aged 12–18 months ***Admission criteria:*** WHZ <−2 and ≥−3 without bipedal oedema ***Recovery*** from MAM was defined as a WHZ ≥−2	***Group 1:*** 204 kcal/day of microbiota-directed complementary food prototype (MDCF-2) ***Group 2:*** 247 kcal/day of ready-to-use supplementary food (RUSF) ***Duration:*** 3 months intervention, 1 month follow-up period	Weekly rate of change in the WHZ, WAZ, MUAC, and LAZ medical complications, plasma proteomic profile, and gut microbiota configuration
5.	Fabiansen et al., 2017 [13] (Plus a nested observational cohort study (Fabiansen 2016 [30]))	Randomised 2 × 2 × 3 factorial trial Province du Passoré in the Northern Region of Burkina Faso	1609 children aged 6–23 months ***Admission criteria:*** WHZ <−2 and ≥−3 (WHO Growth Standards) or MUAC <12.5 cm and ≥11.5 cm ***Recovery*** from MAM was defined as WHZ >−2.0 and MUAC >12.5 cm during at least 2 consecutive follow-up visits The nested observational cohort study included a subgroup of children who were MAM by MUAC only but had a WHZ > −2	Children received one of 500 kcal/day of one 12 food products. Products were either CSB or LNS, with either soy isolate or dehulled soy and either 0%, 20% or 50% of protein from milk. ***Duration:*** 12 weeks	The primary outcome was a fat-free mass index. Other outcomes included: weight, length, knee-heel length gain, MUAC, triceps skinfold, nutritional recovery, and weight MUAC, micronutrient status, acceptability, development, and physical activity
6.	Griswold et al., 2021 [31]	cRCT Sierra Leone	2691 children with MAM aged 6–59 months ***Admission criteria:*** MUAC <12.5 cm and ≥11.5 cm ***Recovery*** from MAM was defined as achieving a MUAC >12.5 cm by 12 weeks	***Group 1:*** 550 kcal/d of CSB+ w/oil ***Group 2:*** 550 kcal/d of CSWB w/oil ***Group 3:*** 550 kcal/d RUSF ***Group 4:*** 550 kcal/d SC+ w/A ***Duration:*** ∼12 weeks	Recovery, deterioration to SAM, death, default, and cost-effectiveness
7.	Kajjura et al., 2019 [32]	cRCT 24 rural parishes Arua district, North Western Uganda	220 mother–child pairs aged 6–18 months ***Admission criteria:*** WHZ <−2 and ≥−3 (WHO Growth Standards) ***Recovery*** from MAM was defined as WHZ >−2.0	***Intervention group:*** 675 kcal/day of an active malt, extruded maize and soy sorghum-based porridge ***Control group:*** 600 kcal/day of CSB Plus ***Duration:*** 3 months	Weight, length, WAZ Score, LAZ score, WLZ, and haemoglobin level
8.	Karakochuk et al., 2012 [33]	cRCT Ten health centres and health posts in the northern region of the Sidama zone, Ethiopia	1125 children aged 6–60 months ***Admission criteria:*** WFH ≥70 to <80% according to NCHS growth standards. ***Recovery*** from MAM was defined as WFH ≥85% on 2 consecutive visits	***Group 1:*** 500 kcal/day of supplementary Plumpy (n = 375) ***Group 2:*** CSB 1413 kcal/day of CSB and vegetable oil (300 g CSB and 32 g oil) was given biweekly (n = 750) ***Duration:*** 16 weeks	Recovery, default, transport, non-response, and mortality
9.	Kohlmann et al., 2019 [34]	Randomised, double-blind controlled study 29 clinics, Brong Ahafo region of Ghana	1270 children 6–59 months ***Admission criteria:*** Children experiencing acute malnutrition and passing the appetite test. MAM was defined as not having SAM and having WHZ <−2 and ≥−3 and/or MUAC of < 12.5 cm >11.5 cm ***Recovery*** criteria were defined as MUAC >12.4 cm and WHZ > −2	***Intervention group:*** 75 kcal/kg/day^2^ alternative RUTF (A-RUTF), where half the amount of peanut was replaced with local soybean and sorghum flour and 50% of protein from dairy came from a combination of whey protein concentrate and non-fat dried milk. ***Control:*** 75 kcal/kg/day standard RUTF ***Duration:*** 12 weeks	Primary outcome: Recovery Secondary outcomes: rates of weight and MUAC gain, number of visits, cost of RUTF per child recovered, and adverse events
10.	La Grone et al., 2012 [14] (Plus two linked observational follow-up studies (Chang et al., 2013 [28]; Trehan et al., 2015 [39]))	RCT Rural setting in South TFC, Malawi	2890 children aged 6–59 month ***Admission criteria:*** WHZ <−2 and ≥−3 without bipedal oedema ***Recovery*** from MAM was defined as a WHZ ≥−2 Linked observational studies followed up 2349 recovered children for 12 months. Chang et al. followed up on 1967 children who were treated until recovery (treat-to-goal), while Trehan compared these children with a smaller sample of 382 children who were treated for a fixed 12 weeks	***Group 1:*** 75 kcal/kg/day of CSB++ (n = 948) ^2^ ***Group 2:*** 75 kcal/kg/day soy RUSF (n = 964) ***Group 3:*** 75 kcal/kg/day soy/whey RUSF (n = 978) ***Duration:*** Until recovery up to 12 weeks	Recovered, developed SAM, remained MAM, died, defaulted time to recovery, rate of adverse events, and rates of gain in weight, length, and MUAC, relapse, and sustained recovery
11.	Matilsky et al., 2009 [36]	RCT Rural setting in the southern region of Malawi	1362 children aged 6–60 months ***Admission criteria:*** WHZ <−2 and ≥−3 without bipedal oedema ***Recovery*** from MAM was defined as a WHZ ≥−2	***Group 1:*** 75 kcal/kg/day of a milk/peanut fortified spread (Nutriset) (n = 465) ^2^ ***Group 2:*** 75 kcal/kg/day of a soy/peanut fortified spread (Nutriset) (n = 450) ***Group 3:*** 75 kcal/kg/day of a corn–soy blend (n = 447) ***Duration:*** Up to 8 weeks	Recovery, rates of gain in weight, stature, MUAC, and adverse outcomes
12.	Medoua et al., 2015 [37]	RCT Mvog-Beti and Evodoula health districts in the Centre region of Cameroon	81 children aged 25–59 months ***Admission criteria:*** WHZ <−2 and ≥−3 without bipedal oedema ***Recovery*** from MAM was defined as a WHZ ≥−2	***Group 1:*** 40 kcal/kg/day CSB+ with soy oil (n = 41) ^3^ ***Group 2:*** 40 kcal/kg/day RUSF (n = 40) ***Duration***: 8 weeks	Recovery rate, time to recovery, and rates of gain in weight and MUAC
13.	Nackers et al., 2010 [10]	RCT Two supplementary feeding centres in the remote villages of Mallawa and Bangaza, Magaria department, Zinder region, South of Niger	807 children aged 6–59 months ***Admission criteria:*** WHM from 70% to <80% NCHS reference, without oedema and with a MUAC ≥110 mm and less than 135 mm. ***Recovery*** was defined as WHM% ≥ 85% for 2 consecutive weeks	***Group 1:*** 1231 kcal/day of CSB with vegetable oil and sugar (n = 406) ***Group 2:*** 1000 kcal/day of RUTF Plumpy’nut) (n = 401) ***Duration:*** 16 weeks	Weight gain, recovery rate, mortality, non-responder and defaulter rates, length of stay, MUAC and height gain, haemoglobin, and relapse
14.	Nane et al., 2021 [40]	RCT Damot Pulassa district, Wolaita, Southern Ethiopia	324 children aged 6–59 months ***Admission criteria:*** WHZ <−2 and ≥−3 (WHO Growth Standards) or MUAC <12.5 cm and ≥11.5 cm ***Recovery*** percentage of children who attained a MUAC ≥12.5 cm and/or WHZ ≥−2 without bipedal edema at the end of 12 weeks.	***Group 1:*** 699 kcal/day of Local ingredients base supplement with 8 mL of refined deodorized and cholesterol-free sunflower oil/day ***Group 2:*** 751 kcal/day of CSB+ with 16 mL of refined deodorized and cholesterol- free sunflower oil/day ***Duration:*** 12 weeks	Recovery rate, weight gain, time to recovery, MUAC gain, and length gain
15.	Nikiema et al., 2014 [23]	cRCT Rural setting in Hounde, Burkina Faso	1974 children aged 6–24 months of age ***Admission criteria:*** WHZ < −2 and ≥−3 without bipedal oedema ***Recovery*** from MAM was defined as a WHZ ≥ −2	***Group 1:*** Child-centred nutrition counselling only (n = 605). ***Group 2:*** 273 kcal/day of corn–soy blend ++ (CSB++) (n = 675) ***Group 3:*** 258 kcal/day of a locally produced peanut and soy-based ready-to-use supplementary food (RUSF) (n = 694) ***Duration:*** 12 weeks	Recovered, died or dropped out, attendance, time to recovery, weight, length, and daily MUAC gains
16.	Roediger et al., 2020 [38]	RCT Stable setting, southern region of Malawi. 27 Feeding sites	1737 children aged 6–59 months ***Admission criteria:*** WHZ < −2 and ≥−3 (WHO Growth Standards) or MUAC < 12.5 cm and ≥11.5 cm ***Recovery*** from MAM was defined as WHZ > −2.0 and MUAC > 12.5 cm during at least 2 consecutive follow-up visits	***Intervention group:*** 75 kcal/kg/day of a high protein RUSF (HiPro-RUSF)^2^ ***Control:*** 75 kcal/kg/day of a standard control RUSF. The two RUSFs were isonitrogenous, but the HiPro-RUSF contained non-fat dried skim milk and whey permeate, whereas C-RUSF contained whey permeate and whey protein concentrate ***Duration:*** Until recovery up to 12 weeks	Recovery, deterioration to SAM, lost to follow-up, average weight and MUAC gain, and time to recovery
17.	Stobaugh et al., 2016 [15]	RCT Stable setting in Rural Malawi (South)	2230 children aged 6–59 months ***Admission:*** MAM, as defined by MUAC of 11.5–12.4 cm without bipedal oedema ***Recovery:*** MUAC of 12.5 cm without bipedal oedema within 12 weeks of therapy	***Group 1:*** 75 kcal/kg/day of soy RUSF^2^ ***Group 2:*** 75 kcal/kg/d of a novel whey RUSF (combination of 4.9% WPC80 and 18.7% whey permeate) ***Duration:*** 12 weeks	Recovery, deterioration to SAM, default, died, MUAC, WHZ, weight gain, and height gain

^1^ Abbreviations: FBF: fortified blended foods; LNS: lipid-based nutrient supplements; cRCT: cluster randomised trial; WHZ: weight-for-height z-score; WHO: World Health Organization; NCHS: National Center for Health Statistics; MUAC: mid-upper arm circumference; LAZ: length for age z-score; CSB: corn–soy blend; SFC: supplementary feeding centre; WHM: weight-for-height median; CSB + w/oil, CSB plus with fortified vegetable oil; CSWB w/oil, corn–soy–whey blend with fortified vegetable oil; RUSF: ready to use supplementary food; SC+ w/A, Supercereal Plus with amylase. ^2^ For an average weight of 7 kg, a dose of 75 kcal/kg/day equates to 525 kcal/day, ^3^ For an average weight of 7 kg, a dose 40 kcal/kg/day equates to 280 kcal/day.

## Supplementary Materials

The following supporting information can be downloaded at: https://www.mdpi.com/article/10.3390/nu15051076/s1, Annex 1. PRISMA checklist, [59]. Annex 2. Supplementary figures S1–S45 (Subquestion 1): Figure S1. Quality of included studies; Figure S2. Forest plot of enhanced FBF compared to LNS—Outcome: Deterioration to SAM; Figure S3. Forest plot of enhanced FBF compared to LNS—Outcome: WHZ; Figure S4. Forest plot of enhanced FBF compared to LNS—Outcome: MUAC gain (in cm); Figure S5. Forest plot of enhanced FBF compared to LNS—Outcome: Weight gain (g/kg/day); Figure S6. Forest plot of enhanced FBF compared to LNS—Outcome: Height gain (cm); Figure S7. Forest plot of enhanced FBF compared to LNS—Outcome: Sustained recovery; Figure S8. Forest plot of enhanced FBF compared to LNS—Outcome: Non-response; Figure S9. Forest plot of enhanced FBF compared to LNS—Outcome: Time to recovery (in days); Figure S10. Forest plot of enhanced FBF compared to LNS—Outcome: Relapse; Figure S11. Forest plot of enhanced FBF vs. LNS—Outcome: Recovery (subgroup analysis by dose); Figure S12. Forest plot of enhanced FBF vs. LNS—Outcome: Deterioration to severe wasting (subgroup analysis by dose); Figure S13. Forest plot of enhanced FBF vs. LNS—Outcome: WHZ (subgroup analysis by dose); Figure S14. Forest plot of enhanced FBF vs. LNS—Outcome: MUAC gain (subgroup analysis by dose); Figure S15. Forest plot of enhanced FBF vs. LNS—Outcome: Weight gain (subgroup analysis by dose); Figure S16. Forest plot of enhanced FBF vs. LNS—Outcome: Height gain (subgroup analysis by dose); Figure S17. Forest plot of enhanced FBF vs. LNS—Outcome: Non-response (subgroup analysis by dose); Figure S18. Forest plot of enhanced FBF vs. LNS—Outcome: Time to recovery (subgroup analysis by dose); Figure S19. Forest plot of enhanced FBF vs. LNS—Outcome: Recovery (subgroup analysis by treatment length); Figure S20. Forest plot of enhanced FBF vs. LNS—Outcome: Deterioration to severe wasting (subgroup analysis by treatment length); Figure S21. Forest plot of enhanced FBF vs. LNS—Outcome: WHZ (subgroup analysis by treatment length); Figure S22. Forest plot of enhanced FBF vs. LNS—Outcome: MUAC gain (subgroup analysis by treatment length); Figure S23. Forest plot of enhanced FBF vs. LNS—Outcome: Weight gain (subgroup analysis by treatment length); Figure S24. Forest plot of enhanced FBF vs. LNS—Outcome: Non-response (subgroup analysis by treatment length); Figure S25. Forest plot of enhanced FBF vs. LNS—Outcome: Time to recovery (subgroup analysis by treatment length); Figure S26. Forest plot of CSB compared to LNS—Outcome: Deterioration to severe wasting; Figure S27. Forest plot of CSB compared to LNS—Outcome: WHZ; Figure S28. Forest plot of CSB compared to LNS—Outcome: HAZ; Figure S29. Forest plot of CSB compared to LNS—Outcome: MUAC gain (mm/day); Figure S30. Forest plot of CSB compared to LNS—Outcome: Weight gain (g/kg/day); Figure S31. Forest plot of CSB compared to LNS—Outcome: Non-response; Figure S32. Forest plot of CSB compared to LNS—Outcome: Time to recovery; Figure S33. Forest plot of CSB compared to LNS—Outcome: Relapse; Figure S34. Forest plot of CSB vs. LNS—Outcome: Recovery rate (subgroup analysis by dose); Figure S35. Forest plot of CSB vs. LNS—Outcome: Deterioration to severe wasting (subgroup analysis by dose); Figure S36. Forest plot of CSB vs. LNS—Outcome: Non-response (subgroup analysis by dose); Figure S37. Forest plot of CSB vs. LNS—Outcome: Recovery rate (subgroup analysis by duration); Figure S38. Forest plot of CSB vs. LNS—Outcome: Deterioration to severe wasting (subgroup analysis by duration); Figure S39. Forest plot of CSB vs. LNS—Outcome: Non-response (subgroup analysis by duration); Figure S40. Forest plot of locally produced FBF compared to LNS—Outcome: Weight for height z-score gain during the 12-week intervention period; Figure S41. Forest plot of locally produced FBF compared to LNS—Outcome: Change in MUAC over the 12-week intervention period; Figure S42. Forest plot of locally produced FBF compared to LNS—Outcome: Weight gain (kg) over the 12-week intervention period; Figure S43. Forest plot of locally produced FBF compared to LNS—Outcome: Height gain (cm) over the 12-week intervention period; Figure S44. Forest plot of locally produced FBF compared to LNS—Outcome: Non-response; Figure S45. Forest plot of locally produced FBF compared to LNS—Outcome: Time to recovery (weeks). Annex 3. Supplementary figures S46–S78 (Subquestion 2): Figure S46. Forest plot of Enhanced FBF compared to locally produced FBFs—Outcome: Deterioration to severe wasting during the intervention period; Figure S47. Forest plot of Enhanced FBF compared to locally produced FBFs—Outcome: WHZ; Figure S48. Forest plot of Enhanced FBF compared to locally produced FBFs—Outcome: WAZ; Figure S49. Forest plot of Enhanced FBF compared to locally produced FBFs—Outcome: HAZ; Figure S50. Forest plot of Enhanced FBF compared to locally produced FBFs—Outcome: MUAC gain (in cm); Figure S51. Forest plot of Enhanced FBF compared to locally produced FBFs—Outcome: Weight gain; Figure S52. Forest plot of Enhanced FBF compared to locally produced FBFs—Outcome: Height gain; Figure S53. Forest plot of Enhanced FBF compared to locally produced FBFs—Outcome: Non-response; Figure S54. Forest plot of Enhanced FBF compared to locally produced FBFs—Outcome: Time to recovery; Figure S55. Forest plot of Enhanced FBFs without milk compared to enhanced FBFs with milk—Outcome: Recovery; Figure S56. Comparison of RUTF vs. RUSF—Outcome: Recovery; Figure S57. Comparison of RUTF vs. RUSF—Outcome: MUAC gain; Figure S58. Comparison of RUTF vs. RUSF—Outcome: Weight gain; Figure S59. Comparison of RUTF vs. RUSF—Outcome: Relapse; Figure S60. Forest plot of RUSF without animal protein vs. RUSF with animal protein—Outcome: Recovery rate; Figure S61. Forest plot of RUSF without animal protein vs. RUSF with animal protein—Outcome: Deterioration to severe wasting; Figure S62. Forest plot of RUSF without animal protein vs. RUSF with animal protein—Outcome: WHZ score; Figure S63. Forest plot of RUSF without animal protein vs. RUSF with animal protein—Outcome: WAZ; Figure S64. Forest plot of RUSF without animal protein vs. RUSF with animal protein—Outcome: HAZ; Figure S65. Forest plot of RUSF without animal protein vs. RUSF with animal protein—Outcome: MUAC gain; Figure S66. Forest plot of RUSF without animal protein vs. RUSF with animal protein—Outcome: Weight gain; Figure S67. Forest plot of RUSF without animal protein vs. RUSF with animal protein—Outcome: Height gain; Figure S68. Forest plot of RUSF without animal protein vs. RUSF with animal protein—Outcome: Sustained recovery; Figure S69. Forest plot of RUSF without animal protein vs. RUSF with animal protein—Outcome: Non-response; Figure S70. Forest plot of RUSF without animal protein vs. RUSF with animal protein—Outcome: Time to recovery; Figure S71. Forest plot of RUSF without animal protein vs. RUSF with animal protein—Outcome: Relapse; Figure S72: Forest plot of protein-optimised RUSF vs. RUSF—Dichotomous outcome: Anthropometric recovery rate, deterioration to SAM and non-response; Figure S73: Forest plot of protein-optimised RUSF vs. RUSF—Dichotomous outcome: MUAC gain, Weight gain, Time to recovery; Figure S74: Forest plot of RUTF vs. alternative RUTF—Outcome: Anthropometric recovery rate; Figure S75: Forest plot of RUTF vs. alternative RUTF—Anthropometric outcome: Weight and MUAC gain; Figure S76. Meta-regression of the relationship between RUTF dose and anthropometric recovery; Figure S77. Meta-regression of the relationship between RUTF dose and MUAC gain; Figure S78. Meta-regression of the relationship between fixed RUTF duration and anthropometric recovery. Annex 4. Supplementary Tables: Table S1. Search strategy; Table S2. Evidence Profile for Comparison 1c. Enhanced commercially produced and/or imported FBFs vs. LNS; Table S3. Evidence Profile for comparison 1.b. CSB vs. LNS; Table S4. Evidence Profile for Comparison 1c (locally produced FBF compared to LNS); Table S5. Evidence Profile for comparison 2.a.1. (Enhanced commercially produced and/or imported FBFs vs. locally produced FBFs); Table S6. Evidence Profile for comparison 2b.1 (RUTF vs. RUSF)

## Abbreviations

CI: Confidence interval; cRCTs: Cluster randomised controlled trials; CSB: Corn–soy blends; CSWB: Corn–soy–whey blend; FBF: Fortified blended flours; GRADE: Grading of Recommendations, Assessment, Development, and Evaluation; HAZ: Height-for-age-z-score; LAZ: Length-for-age z-scores; LNS: Lipid-based nutrient supplements; MAM: Moderate acute malnutrition; MD: Mean difference; MUAC: Mid-upper arm circumference; RCT: Randomised controlled trials; RR: Risk ratio; RUSF: Ready-to-use supplementary food; RUTF: Ready-to-use therapeutic food; SAM: Severe acute malnutrition; SC + A: Supercereal plus with amylase; UNICEF: United Nations Children’s Fund; WAZ: Weight-for-age z-score; WHO: World Health Organization; WHZ: Weight-for-height z-score; WLZ: Weight-for-length z-scores.
