# Supplementary material for: Effectiveness of Dietary Management for Moderate Wasting among Children > 6 Months of Age—A Systematic Review and Meta-Analysis Exploring Different Types, Quantities, and Durations
Source: Nutrients. 2023 Feb 21;15(5):1076. doi: 10.3390/nu15051076 (PMC10005276; doi:10.3390/nu15051076)
Supplement: Supplementary file 1 [file nutrients-15-01076-s001.zip › Annex 2. Supplementary Figures S1-S45_Subquestion 1.pdf]

Supplementary Figure S1. Quality of inlcuded studies

Supplementary Figure S1a. Quality of included cluster randomised controlled studies

Study ID	D1a: Randomis-ation process	D1b: Timing of identification or recruitment of participants	D2: Deviations from the intended intervention	D3: Missing outcome data	D4: Measure ment of the outcome	D5: Selection of the reported result	Overall
Ackatia-Armah 2015	!	+	!	+	+	-	-
Amegovu 2014	!	!	+	+	!	!	!
Bailey 2020	+	+	+	+	!	+	!
Griswold 2021	-	-	+	+	+	+	-
Kajjura 2019	!	+	+	+	+	!	!
Karakochuk 2012	+	-	+	+	+	!	-
Nikiema 2014	+	+	+	+	-	+	!

Supplementary Figure S1b. Quality of included individually randomised controlled studies

Study ID	D1: Randomis-ation process	D2: Deviations from the intended interventions	D3: Missing outcome data	D4: Measure ment of the outcome	D5: Selection of the reported result	Overall
Chen 2021	+	+	+	!	+	!
Fabiansen 2017	+	!	+	+	+	!
Kohlman 2019	+	+	+	+	-	!
LaGrone 2012	+	!	+	+	+	!
Matilsky 2009	+	+	+	+	+	+
Medoua 2016	+	+	+	+	!	!
Nackers 2010	+	+	-	+	!	-
Nane 2021	+	+	+	+	+	+
Roediger 2020	+	+	+	+	+	+
Stobaugh 2016	+	+	+	+	+	+

+ Low risk
! Some concerns
- High risk

Supplementary Figure S2. Forest plot of enhanced FBF compared to LNS - Outcome: Deterioration to SAM

Study or Subgroup	Enhanced FBFs Events	Enhanced FBFs Total	LNS Events	LNS Total	Weight	Risk Ratio M-H, Random, 95% CI
Fabiansen 2017 (1)	70	800	59	809	14.6%	1.20 [0.86, 1.67]
Griswold 2021 (2)	116	663	55	256	16.7%	0.81 [0.61, 1.08]
Griswold 2021 (3)	120	663	55	256	16.8%	0.84 [0.63, 1.12]
Griswold 2021 (4)	99	579	55	256	16.3%	0.80 [0.59, 1.07]
LaGrone 2012 (5)	29	444	47	906	10.5%	1.26 [0.80, 1.97]
LaGrone 2012 (6)	30	444	39	918	10.1%	1.59 [1.00, 2.53]
Medoua 2016 (7)	2	41	1	40	0.6%	1.95 [0.18, 20.68]
Nikièma 2014 (8)	67	675	57	694	14.4%	1.21 [0.86, 1.69]
**Total (95% CI)**		**4309**		**4135**	**100.0%**	**1.03 [0.86, 1.23]**
Total events	533		368			

Heterogeneity: Tau² = 0.03; Chi² = 13.30, df = 7 (P = 0.07); I² = 47%
Test for overall effect: Z = 0.30 (P = 0.76)

Risk Ratio M-H, Random, 95% CI

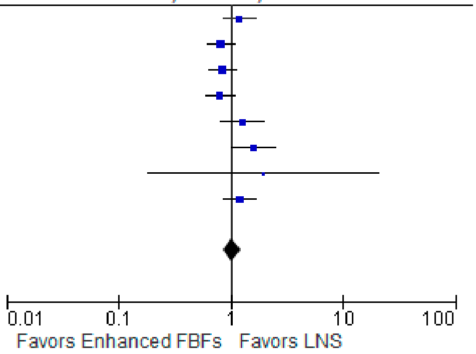

Footnotes
(1) Enhanced FBFs vs LNS; developed SAM at any point during the 12 week intervention
(2) Super Cereal Plus with amylase (SC+A) vs RUSF; developed SAM in the 12 week intervention period.
(3) CSB+w/oil vs RUSF; developed SAM in the 12 week intervention period.
(4) Corn Soy Whey Blend w/oil vs RUSF; developed SAM in the 12 week intervention period.
(5) CSB++ vs Soy RUSF; Developed severe wasting (WHZ <-3) during the intervention period.
(6) SB++ vs Soy/Whey RUSF; Severe wasting (WHZ <-3) during the intervention period.
(7) CSB+ with soy oil vs RUSF; Developed SAM (Severe wasting (WHZ< − 3)).
(8) CSB++ vs Locally produced RUSF; developed SAM during the intervention period.

Supplementary Figure S3. Forest plot of enhanced FBF compared to LNS - Outcome: WHZ

Study or Subgroup	Enhanced FBFs Mean	Enhanced FBFs SD	Enhanced FBFs Total	LNS Mean	LNS SD	LNS Total	Weight	Mean Difference IV, Random, 95% CI
Ackatia-Armah 2015 (1)	0.76	1.12	342	0.94	1.05	335	9.1%	-0.18 [-0.34, -0.02]
LaGrone 2012 (2)	-1.68	0.67	444	-1.59	0.6	918	45.2%	-0.09 [-0.16, -0.02]
LaGrone 2012 (3)	-1.68	0.67	444	-1.61	0.63	906	43.7%	-0.07 [-0.14, 0.00]
Medoua 2016 (4)	-1.58	0.89	41	-1.34	0.7	40	2.0%	-0.24 [-0.59, 0.11]
**Total (95% CI)**			**1271**			**2199**	**100.0%**	**-0.09 [-0.14, -0.04]**

Heterogeneity: Tau² = 0.00; Chi² = 2.14, df = 3 (P = 0.54); I² = 0%
Test for overall effect: Z = 3.67 (P = 0.0002)

Mean Difference IV, Random, 95% CI

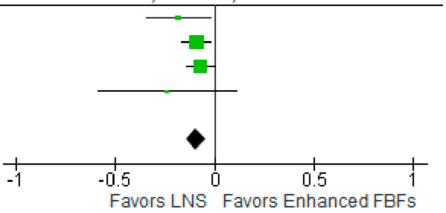

Footnotes
(1) CSB++ vs Supplemntary Plumpy/Nutriset; Change in WHZ during 12 weeks of treatment.
(2) CSB++ vs Soy/Whey RUSF; WHZ at the end of the intervention.
(3) CSB++ vs Soy RUSF; WHZ at the end of the intervention.
(4) CSB+ with soy oil vs RUSF; WHZ at the end of the intervention.

Supplementary Figure S4. Forest plot of enhanced FBF compared to LNS - Outcome: MUAC gain (in cm)

Study or Subgroup	Enhanced FBFs Mean	Enhanced FBFs SD	Enhanced FBFs Total	LNS Mean	LNS SD	LNS Total	Weight	Mean Difference IV, Random, 95% CI
Ackatia-Armah 2015 (1)	0.9	0.94	342	1.1	0.91	335	41.0%	-0.20 [-0.34, -0.06]
LaGrone 2012 (2)	1.09	3.36	444	1.09	3.6	906	19.6%	0.00 [-0.39, 0.39]
LaGrone 2012 (3)	1.09	3.36	444	1.76	3.69	918	19.5%	-0.67 [-1.06, -0.28]
Medoua 2016 (4)	0.56	1.06	41	0.78	0.67	40	19.9%	-0.22 [-0.61, 0.17]
**Total (95% CI)**			**1271**			**2199**	**100.0%**	**-0.26 [-0.48, -0.03]**

Heterogeneity: Tau² = 0.03; Chi² = 6.32, df = 3 (P = 0.10); I² = 53%
Test for overall effect: Z = 2.24 (P = 0.02)

Mean Difference IV, Random, 95% CI

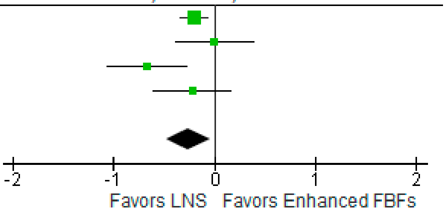

Footnotes
(1) CSB++ vs Supplementary Plumpy; MUAC gain in cm over the intervention period.
(2) CSB++ vs Soy RUSF; MUAC gain in cm over the intervention period.
(3) CSB++ vs Soy/Whey RUSF; MUAC gain in cm over the intervention period.
(4) CSB+ with soy oil vs RUSF; MUAC gain in cm over the intervention period.

Supplementary Figure S5. Forest plot of enhanced FBF compared to LNS - Outcome: Weight gain (g/kg/day)

	Enhanced FBFs			LNS				Mean Difference
Study or Subgroup	Mean	SD	Total	Mean	SD	Total	Weight	IV, Random, 95% CI
Ackatia-Armah 2015 (1)	1.77	1.12	342	1.97	1.24	335	38.9%	-0.20 [-0.38, -0.02]
LaGrone 2012 (2)	3.1	2.4	444	3.4	2.6	906	27.1%	-0.30 [-0.58, -0.02]
LaGrone 2012 (3)	3.1	2.4	444	3.6	2.8	918	26.4%	-0.50 [-0.79, -0.21]
Medoua 2016 (4)	1.83	1.64	41	2.71	1.54	40	7.5%	-0.88 [-1.57, -0.19]
**Total (95% CI)**			**1271**			**2199**	**100.0%**	**-0.36 [-0.56, -0.15]**

Heterogeneity: Tau² = 0.02; Chi² = 5.75, df = 3 (P = 0.12); I² = 48%
Test for overall effect: Z = 3.42 (P = 0.0006)

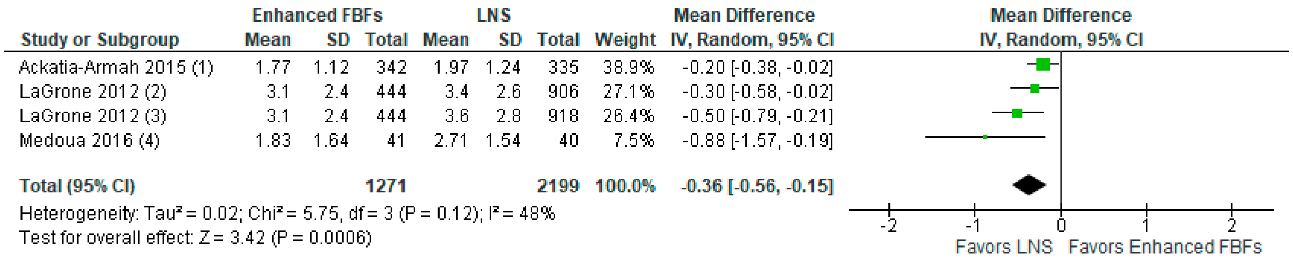

Footnotes
(1) CSB++ vs Supplementary Plumpy; Weight gain in g/kg/day.
(2) CSB++ vs Soy RUSF; Weight gain 4 weeks post enrollment in g/kg/day.
(3) CSB++ vs Soy/Whey RUSF; Weight gain 4 weeks on enrollment in (g/kg/day).
(4) CSB+ with soy oil vs RUSF; Weight gain in g/kg/d over the intervention period.

Supplementary Figure S6. Forest plot of enhanced FBF compared to LNS - Outcome: Height gain (cm)

	Enhanced FBFs			LNS				Mean Difference
Study or Subgroup	Mean	SD	Total	Mean	SD	Total	Weight	IV, Random, 95% CI
Ackatia-Armah 2015 (1)	2.9	1.41	342	2.9	1.37	335	68.5%	0.00 [-0.21, 0.21]
LaGrone 2012 (2)	1.1	3.86	444	1.09	3.69	906	16.1%	0.01 [-0.42, 0.44]
LaGrone 2012 (3)	1.1	3.86	444	1.26	3.95	918	15.5%	-0.16 [-0.60, 0.28]
**Total (95% CI)**			**1230**			**2159**	**100.0%**	**-0.02 [-0.20, 0.15]**

Heterogeneity: Tau² = 0.00; Chi² = 0.44, df = 2 (P = 0.80); I² = 0%
Test for overall effect: Z = 0.26 (P = 0.79)

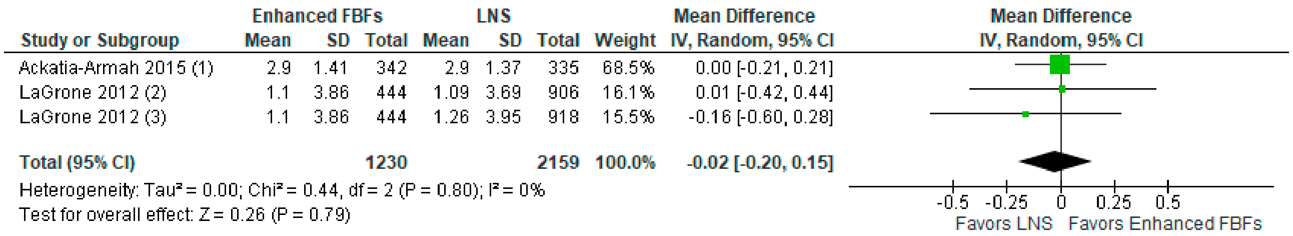

Footnotes
(1) CSB++ vs Supplemntary Plumpy/Nutriset; height gain in cm over the intervention period.
(2) CSB++ vs Soy RUSF; height gain in cm over the intervention period.
(3) CSB++ vs Soy/Whey RUSF; height gain in cm over the intervention period.

Supplementary Figure S7. Forest plot of enhanced FBF compared to LNS - Outcome: Sustained recovery

	Enhanced FBFs		LNS			Risk Ratio
Study or Subgroup	Events	Total	Events	Total	Weight	M-H, Random, 95% CI
LaGrone 2012 (1)	203	327	446	667	51.1%	0.93 [0.84, 1.03]
LaGrone 2012 (2)	202	326	382	647	48.9%	1.05 [0.94, 1.17]
**Total (95% CI)**		**653**		**1314**	**100.0%**	**0.99 [0.87, 1.11]**
Total events	405		828			

Heterogeneity: Tau² = 0.00; Chi² = 2.70, df = 1 (P = 0.10); I² = 63%
Test for overall effect: Z = 0.23 (P = 0.81)

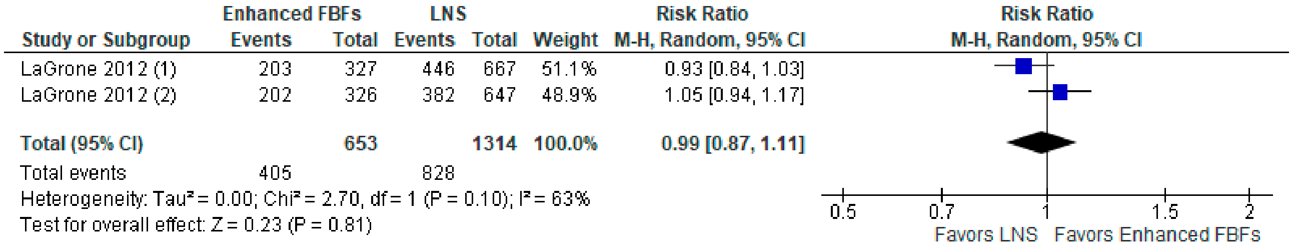

Footnotes
(1) CSB++ vs Soy/Whey RUSF; Sustained recovery at 12 months post discharge.
(2) CSB++ vs Soy RUSF; Sustained recovery at 12 months post discharge.

Supplementary Figure S8. Forest plot of enhanced FBF compared to LNS - Outcome: Non-response

Study or Subgroup	Enhanced FBFs Events	Enhanced FBFs Total	LNS Events	LNS Total	Weight	Risk Ratio M-H, Random, 95% CI
Ackatia-Armah 2015 (1)	115	342	78	335	27.1%	1.44 [1.13, 1.84]
Fabiansen 2017 (2)	192	800	243	809	29.9%	0.80 [0.68, 0.94]
LaGrone 2012 (3)	4	444	5	906	4.8%	1.63 [0.44, 6.05]
LaGrone 2012 (4)	4	444	8	918	5.6%	1.03 [0.31, 3.41]
Medoua 2016 (5)	8	41	6	40	7.8%	1.30 [0.50, 3.41]
Nikièma 2014 (6)	75	675	72	694	24.8%	1.07 [0.79, 1.45]
**Total (95% CI)**		**2746**		**3702**	**100.0%**	**1.10 [0.81, 1.50]**
Total events	398		412			

Heterogeneity: $Tau^2 = 0.08$; $Chi^2 = 16.98$, df = 5 (P = 0.005); $I^2 = 71\%$
Test for overall effect: Z = 0.60 (P = 0.55)

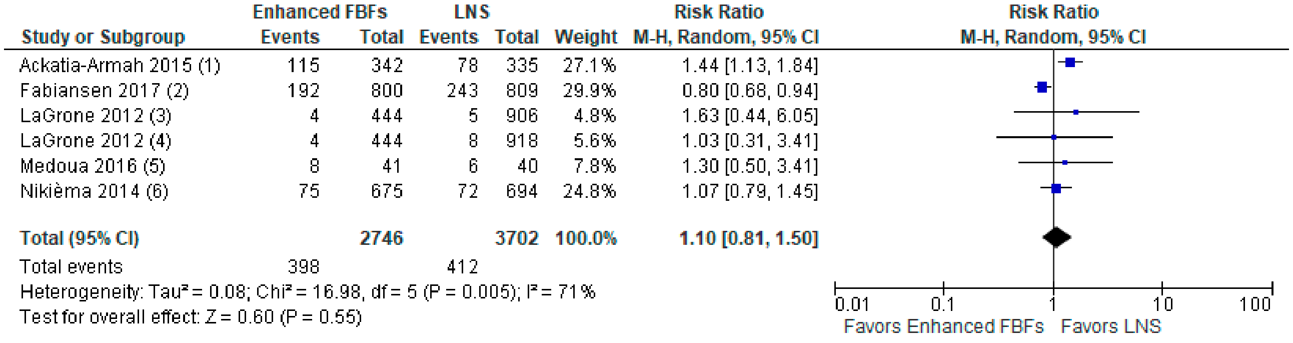

Footnotes
(1) CSB++ vs Supplementary Plumpy; Non-response defined as not recovered by week 12.
(2) Enhanced FBFs vs LNS; Non-response defined as not recovered by week 12.
(3) CSB++ vs Soy RUSF; Non-response defined as still MAM by week 12.
(4) CSB++ vs Soy/Whey RUSF; Non-response defined as still MAM by week 12.
(5) CSB+ with soy oil vs RUSF; continued MAM despite 8 weeks of therapy.
(6) CSB++ vs Locally produced RUSF; Non-response defined as remained MAM at the end of 3 months intervention.

Supplementary Figure S9. Forest plot of enhanced FBF compared to LNS - Outcome: Time to recovery (in days)

Study or Subgroup	Enhanced FBFs Mean	Enhanced FBFs SD	Enhanced FBFs Total	LNS Mean	LNS SD	LNS Total	Weight	Mean Difference IV, Random, 95% CI
Ackatia-Armah 2015 (1)	49	17.22	209	41.3	11.2	245	20.0%	7.70 [4.98, 10.42]
LaGrone 2012 (2)	24.9	17.5	444	22.5	14.2	906	21.6%	2.40 [0.53, 4.27]
LaGrone 2012 (3)	24.9	17.5	444	22.6	15	918	21.6%	2.30 [0.40, 4.20]
Medoua 2016 (4)	51.16	1.45	41	44.17	1.87	40	23.0%	6.99 [6.26, 7.72]
Nikièma 2014 (5)	29.4	39.9	503	36.4	50.4	515	13.8%	-7.00 [-12.58, -1.42]
Total (95% CI)			1641			2624	100.0%	3.20 [-0.06, 6.45]

Heterogeneity: $Tau^2 = 11.84$; $Chi^2 = 58.06$, df = 4 (P < 0.00001); $I^2 = 93\%$
Test for overall effect: Z = 1.92 (P = 0.05)

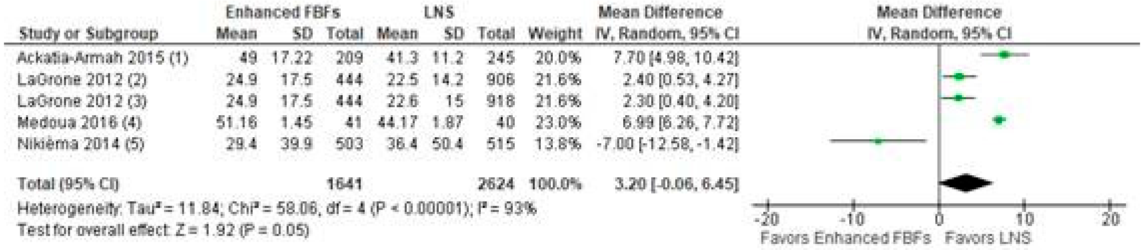

Footnotes
(1) CSB++ vs Supplementary Plumpy; Time to recovery in days.
(2) CSB++ vs Soy RUSF; Time to recovery in days.
(3) CSB++ vs Soy/Whey RUSF; Time to recovery in days.
(4) CSB+ with soy oil vs RUSF; Time to recovery in days
(5) CSB++ vs Locally produced RUSF; Time to recovery in days.

Supplementary Figure S10. Forest plot of enhanced FBF compared to LNS - Outcome: Relapse

Study or Subgroup	Enhanced FBFs Events	Enhanced FBFs Total	LNS Events	LNS Total	Weight	Risk Ratio M-H, Random, 95% CI
LaGrone 2012 (1)	87	327	192	647	51.4%	0.90 [0.72, 1.11]
LaGrone 2012 (2)	86	326	159	667	48.6%	1.11 [0.88, 1.39]
**Total (95% CI)**		**653**		**1314**	**100.0%**	**0.99 [0.81, 1.22]**
Total events	173		351			

Heterogeneity: $Tau^2 = 0.01$; $Chi^2 = 1.74$, df = 1 (P = 0.19); $I^2 = 43\%$
Test for overall effect: Z = 0.07 (P = 0.95)

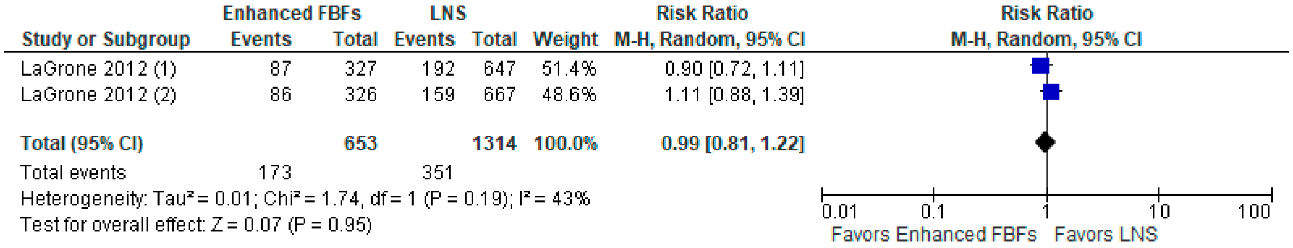

Footnotes
(1) CSB++ vs Soy RUSF; relapsed to MAM: Relapse defined as recovery children who deteriorated to MAM or SAM.
(2) CSB++ vs Soy/Whey RUSF; relapsed to MAM: Relapse defined as recovery children who deteriorated to MAM or SAM.

Supplementary Figure S11. Forest plot of enhanced FBF vs LNS - Outcome: Recovery (subgroup analysis by dose)

Study or Subgroup	Enhanced FBFs Events	Enhanced FBFs Total	LNS Events	LNS Total	Weight	Risk Ratio M-H, Random, 95% CI
**11.1.1 Medium fixed dose**						
Ackatia-Armah 2015 (1)	209	342	245	335	8.5%	0.84 [0.75, 0.93]
Fabiansen 2017 (2)	501	809	552	800	13.3%	0.90 [0.84, 0.96]
Griswold 2021 (3)	415	663	159	256	7.9%	1.01 [0.90, 1.13]
Griswold 2021 (4)	360	579	159	256	7.7%	1.00 [0.89, 1.12]
Griswold 2021 (5)	424	663	159	256	8.0%	1.03 [0.92, 1.15]
**Subtotal (95% CI)**		**3056**		**1903**	**45.5%**	**0.95 [0.88, 1.02]**
Total events	1909		1274			
Heterogeneity: Tau² = 0.00; Chi² = 11.51, df = 4 (P = 0.02); I² = 65%						
Test for overall effect: Z = 1.39 (P = 0.17)						
**11.1.3 Low fixed dose**						
Nikièma 2014 (6)	503	675	515	694	15.0%	1.00 [0.94, 1.07]
**Subtotal (95% CI)**		**675**		**694**	**15.0%**	**1.00 [0.94, 1.07]**
Total events	503		515			
Heterogeneity: Not applicable						
Test for overall effect: Z = 0.13 (P = 0.90)						
**11.1.4 High weight dependent dose**						
LaGrone 2012 (7)	381	444	795	906	18.4%	0.98 [0.93, 1.02]
LaGrone 2012 (8)	382	444	807	918	18.5%	0.98 [0.94, 1.02]
**Subtotal (95% CI)**		**888**		**1824**	**36.9%**	**0.98 [0.95, 1.01]**
Total events	763		1602			
Heterogeneity: Tau² = 0.00; Chi² = 0.00, df = 1 (P = 0.98); I² = 0%						
Test for overall effect: Z = 1.36 (P = 0.17)						
**11.1.5 Low weight dependent dose**						
Medoua 2016 (9)	30	41	34	40	2.6%	0.86 [0.69, 1.08]
**Subtotal (95% CI)**		**41**		**40**	**2.6%**	**0.86 [0.69, 1.08]**
Total events	30		34			
Heterogeneity: Not applicable						
Test for overall effect: Z = 1.30 (P = 0.19)						
**Total (95% CI)**		**4660**		**4461**	**100.0%**	**0.96 [0.93, 1.00]**
Total events	3205		3425			
Heterogeneity: Tau² = 0.00; Chi² = 16.67, df = 8 (P = 0.03); I² = 52%						
Test for overall effect: Z = 1.94 (P = 0.05)						
Test for subgroup differences: Chi² = 2.58, df = 3 (P = 0.46), I² = 0%						

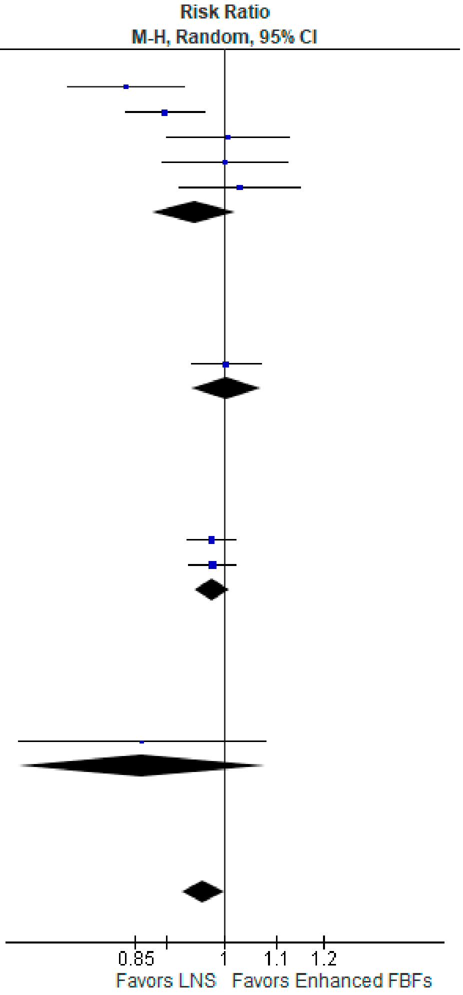

Footnotes
(1) Supplemntary Plumpy/Nutriset; sustained recovery from MAM, defined as WLZ >-2.0 and MUAC >12.5 cm for >=2 follow-up visits
(2) LNS, factorial trial including 12 different products; recovery at 12 weeks (WHZ >= –2 and MUAC >= 125 mm)
(3) SC+ A, Super Cereal Plus with amylase; recovery rate defined as achieving MUAC ≥12.5 cm by the seventh visit and no bipedal edema
(4) CSWB w/oil; recovery rate defined as achieving MUAC ≥12.5 cm by the seventh visit and no bipedal edema
(5) CSB+w/oil; recovery rate defined as achieving MUAC ≥12.5 cm by the seventh visit and no bipedal edema
(6) Locally produced RUSF; recovery rate defined as WHZ ≥ -2 SD
(7) Soy/Peanut RUSF; recovered when they reached a WHZ >= -2
(8) Whey RUSF; recovered when they reached a WHZ >= -2
(9) RUSF produced by using pre-cooked soya and corn flours, peanut paste, sugar, soya oil and a premix containing concentrated minerals and...

Supplementary Figure S12. Forest plot of enhanced FBF vs LNS - Outcome: Deterioration to severe wasting (subgroup analysis by dose)

Study or Subgroup	Enhanced FBFs Events	Enhanced FBFs Total	LNS Events	LNS Total	Weight	Risk Ratio M-H, Random, 95% CI
**11.2.1 Medium fixed dose**						
Fabiansen 2017 (1)	70	800	59	809	14.6%	1.20 [0.86, 1.67]
Griswold 2021 (2)	116	663	55	256	16.7%	0.81 [0.61, 1.08]
Griswold 2021 (3)	120	663	55	256	16.8%	0.84 [0.63, 1.12]
Griswold 2021 (4)	99	579	55	256	16.3%	0.80 [0.59, 1.07]
**Subtotal (95% CI)**		**2705**		**1577**	**64.4%**	**0.89 [0.74, 1.06]**
Total events	405		224			
Heterogeneity: Tau² = 0.01; Chi² = 4.18, df = 3 (P = 0.24); I² = 28%						
Test for overall effect: Z = 1.33 (P = 0.18)						
**11.2.2 Low fixed dose**						
Nikièma 2014 (5)	67	675	57	694	14.4%	1.21 [0.86, 1.69]
**Subtotal (95% CI)**		**675**		**694**	**14.4%**	**1.21 [0.86, 1.69]**
Total events	67		57			
Heterogeneity: Not applicable						
Test for overall effect: Z = 1.10 (P = 0.27)						
**11.2.3 High weight dependent dose**						
LaGrone 2012 (6)	29	444	47	906	10.5%	1.26 [0.80, 1.97]
LaGrone 2012 (7)	30	444	39	918	10.1%	1.59 [1.00, 2.53]
**Subtotal (95% CI)**		**888**		**1824**	**20.5%**	**1.41 [1.02, 1.95]**
Total events	59		86			
Heterogeneity: Tau² = 0.00; Chi² = 0.51, df = 1 (P = 0.48); I² = 0%						
Test for overall effect: Z = 2.09 (P = 0.04)						
**11.2.4 Low weight dependent dose**						
Medoua 2016 (8)	2	41	1	40	0.6%	1.95 [0.18, 20.68]
**Subtotal (95% CI)**		**41**		**40**	**0.6%**	**1.95 [0.18, 20.68]**
Total events	2		1			
Heterogeneity: Not applicable						
Test for overall effect: Z = 0.56 (P = 0.58)						
**Total (95% CI)**		**4309**		**4135**	**100.0%**	**1.03 [0.86, 1.23]**
Total events	533		368			
Heterogeneity: Tau² = 0.03; Chi² = 13.30, df = 7 (P = 0.07); I² = 47%						
Test for overall effect: Z = 0.30 (P = 0.76)						
Test for subgroup differences: Chi² = 7.54, df = 3 (P = 0.06), I² = 60.2%						

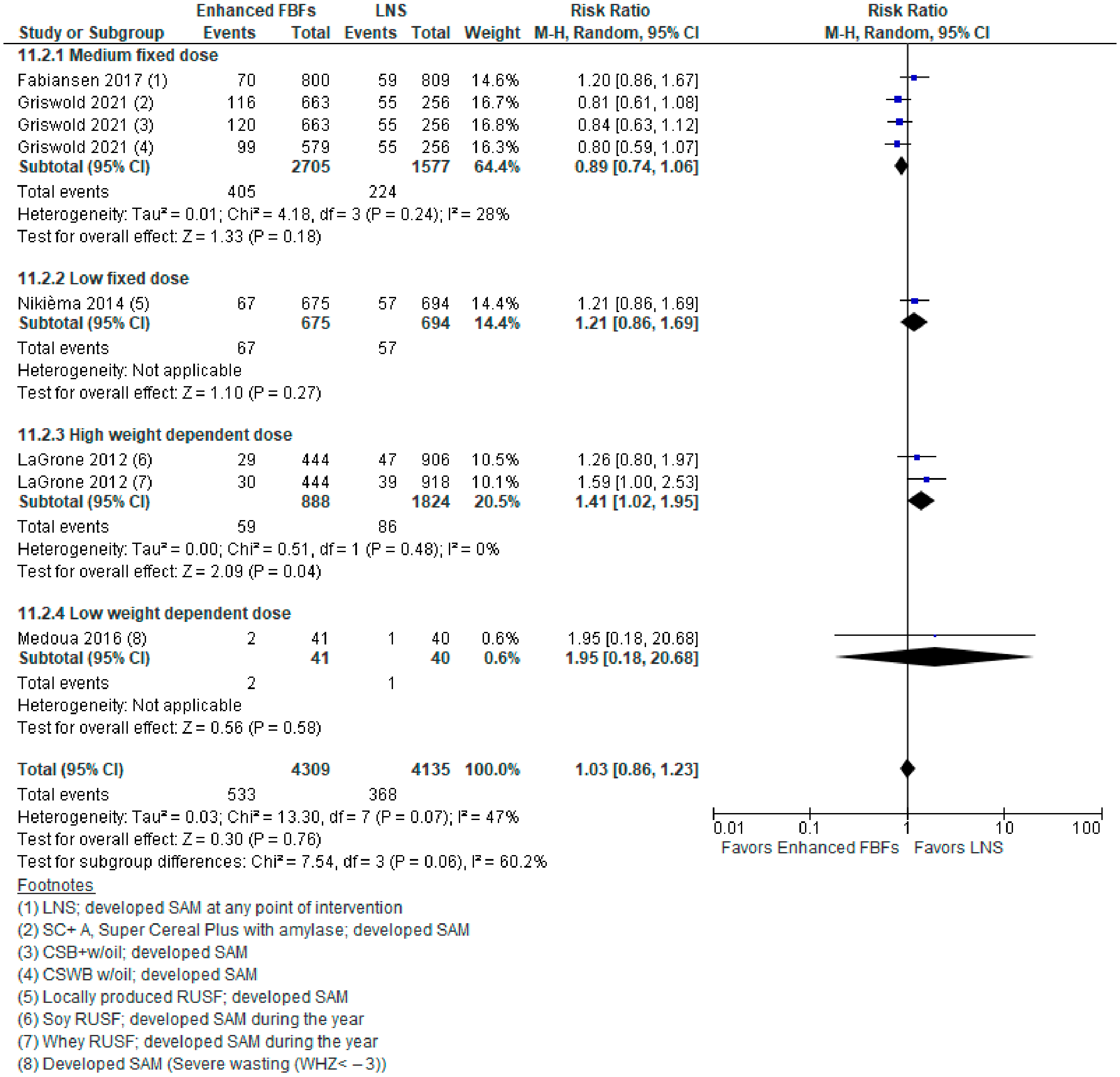

Footnotes
(1) LNS; developed SAM at any point of intervention
(2) SC+ A, Super Cereal Plus with amylase; developed SAM
(3) CSB+w/oil; developed SAM
(4) CSWB w/oil; developed SAM
(5) Locally produced RUSF; developed SAM
(6) Soy RUSF; developed SAM during the year
(7) Whey RUSF; developed SAM during the year
(8) Developed SAM (Severe wasting (WHZ< −3))

Supplementary Figure S13. Forest plot of enhanced FBF vs LNS - Outcome: WHZ (subgroup analysis by dose)

Study or Subgroup	Enhanced FBFs Mean	SD	Total	LNS Mean	SD	Total	Weight	Mean Difference IV, Random, 95% CI
**11.3.1 Medium fixed dose**								
Ackatia-Armah 2015 (1)	0.76	1.12	342	0.94	1.05	335	9.1%	-0.18 [-0.34, -0.02]
**Subtotal (95% CI)**			**342**			**335**	**9.1%**	**-0.18 [-0.34, -0.02]**
Heterogeneity: Not applicable								
Test for overall effect: Z = 2.16 (P = 0.03)								
**11.3.2 High weight dependent dose**								
LaGrone 2012 (2)	-1.68	0.67	444	-1.61	0.63	906	43.7%	-0.07 [-0.14, 0.00]
LaGrone 2012 (3)	-1.68	0.67	444	-1.59	0.6	918	45.2%	-0.09 [-0.16, -0.02]
**Subtotal (95% CI)**			**888**			**1824**	**88.9%**	**-0.08 [-0.13, -0.03]**
Heterogeneity: Tau^2 = 0.00; Chi^2 = 0.14, df = 1 (P = 0.71); I^2 = 0%								
Test for overall effect: Z = 3.00 (P = 0.003)								
**11.3.3 Low weight dependent dose**								
Medoua 2016 (4)	-1.58	0.89	41	-1.34	0.7	40	2.0%	-0.24 [-0.59, 0.11]
**Subtotal (95% CI)**			**41**			**40**	**2.0%**	**-0.24 [-0.59, 0.11]**
Heterogeneity: Not applicable								
Test for overall effect: Z = 1.35 (P = 0.18)								
**Total (95% CI)**			**1271**			**2199**	**100.0%**	**-0.09 [-0.14, -0.04]**
Heterogeneity: Tau^2 = 0.00; Chi^2 = 2.14, df = 3 (P = 0.54); I^2 = 0%								
Test for overall effect: Z = 3.67 (P = 0.0002)								
Test for subgroup differences: Chi^2 = 2.00, df = 2 (P = 0.37), I^2 = 0.1%								

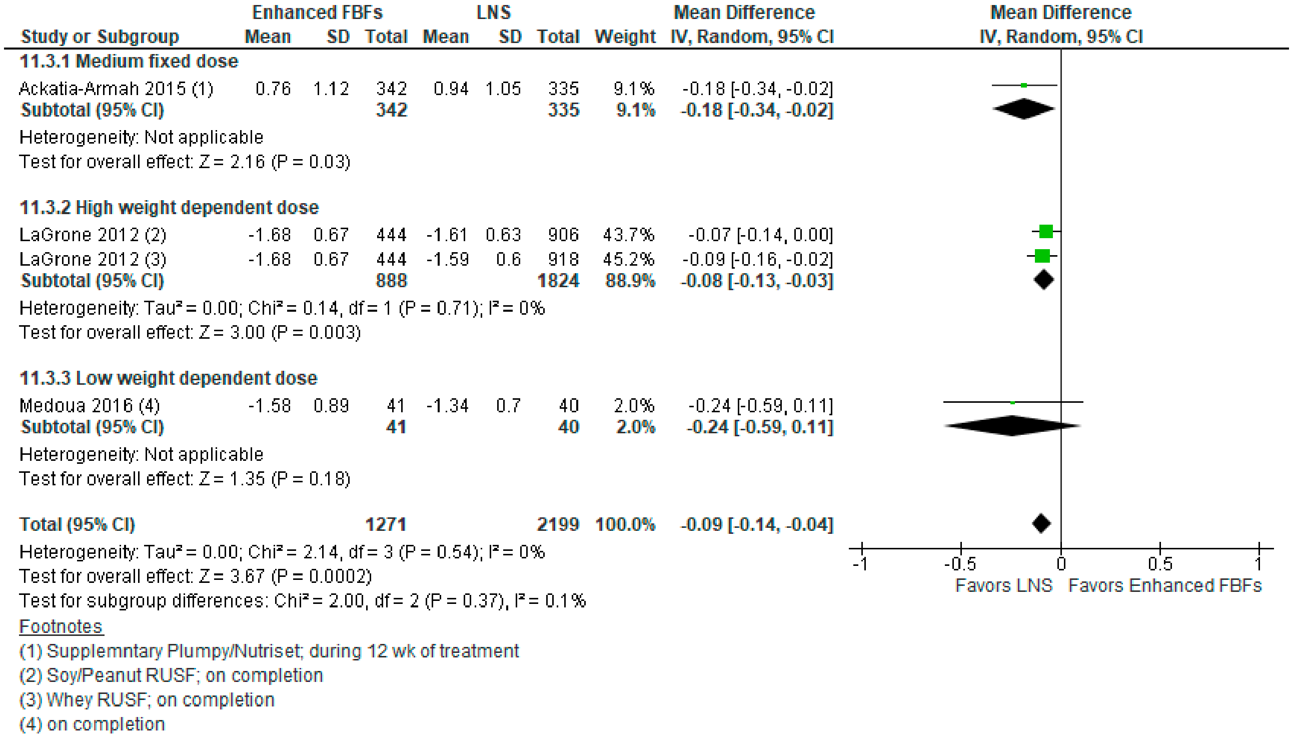

Footnotes
(1) Supplemntary Plumpy/Nutriset; during 12 wk of treatment
(2) Soy/Peanut RUSF; on completion
(3) Whey RUSF; on completion
(4) on completion

Supplementary Figure S14. Forest plot of enhanced FBF vs LNS - Outcome: MUAC gain (subgroup analysis by dose)

Study or Subgroup	Enhanced FBFs Mean	SD	Total	LNS Mean	SD	Total	Weight	Mean Difference IV, Random, 95% CI
**11.4.1 Medium fixed dose**								
Ackatia-Armah 2015 (1)	0.9	0.94	342	1.1	0.91	335	41.0%	-0.20 [-0.34, -0.06]
**Subtotal (95% CI)**			**342**			**335**	**41.0%**	**-0.20 [-0.34, -0.06]**
Heterogeneity: Not applicable								
Test for overall effect: Z = 2.81 (P = 0.005)								
**11.4.2 High weight dependent dose**								
LaGrone 2012 (2)	1.09	3.36	444	1.76	3.69	918	19.5%	-0.67 [-1.06, -0.28]
LaGrone 2012 (3)	1.09	3.36	444	1.09	3.6	906	19.6%	0.00 [-0.39, 0.39]
**Subtotal (95% CI)**			**888**			**1824**	**39.1%**	**-0.33 [-0.99, 0.32]**
Heterogeneity: Tau^2 = 0.18; Chi^2 = 5.61, df = 1 (P = 0.02); I^2 = 82%								
Test for overall effect: Z = 1.00 (P = 0.32)								
**11.4.3 Low weight dependent dose**								
Medoua 2016 (4)	0.56	1.06	41	0.78	0.67	40	19.9%	-0.22 [-0.61, 0.17]
**Subtotal (95% CI)**			**41**			**40**	**19.9%**	**-0.22 [-0.61, 0.17]**
Heterogeneity: Not applicable								
Test for overall effect: Z = 1.12 (P = 0.26)								
**Total (95% CI)**			**1271**			**2199**	**100.0%**	**-0.26 [-0.48, -0.03]**
Heterogeneity: Tau^2 = 0.03; Chi^2 = 6.32, df = 3 (P = 0.10); I^2 = 53%								
Test for overall effect: Z = 2.24 (P = 0.02)								
Test for subgroup differences: Chi^2 = 0.16, df = 2 (P = 0.92), I^2 = 0%								

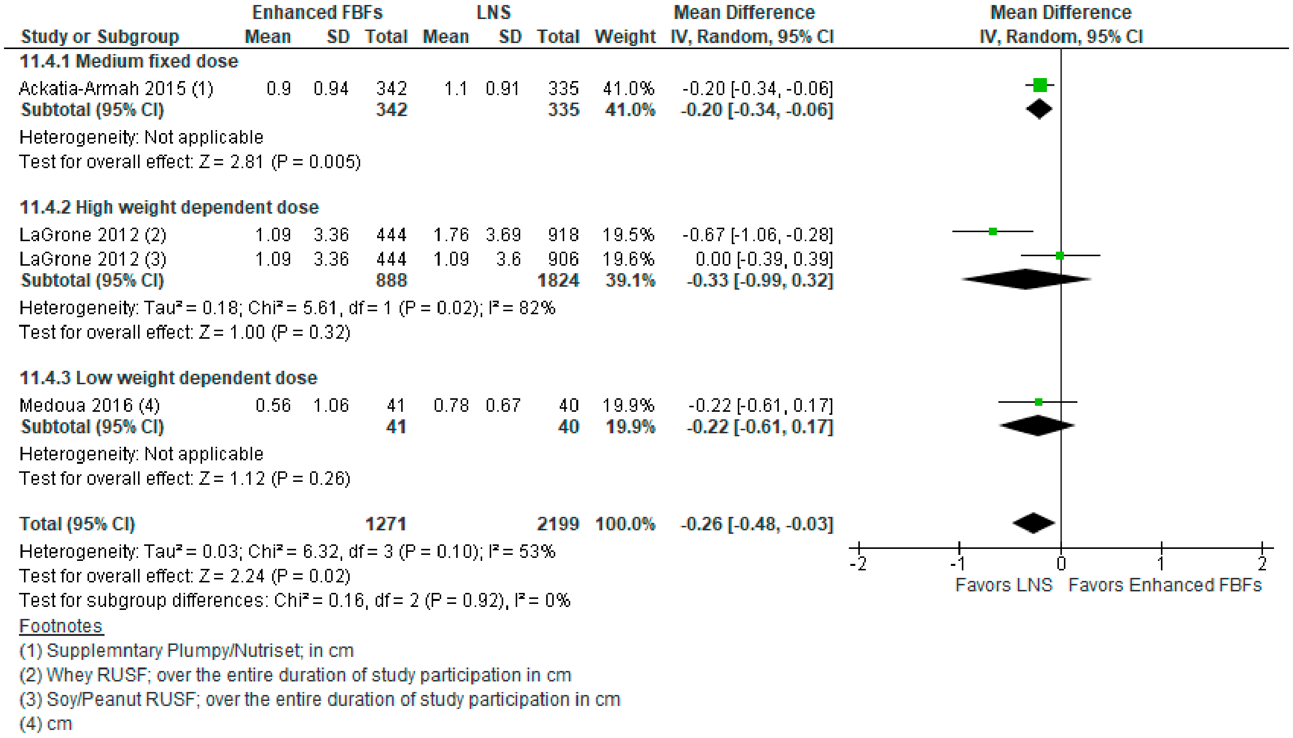

Footnotes
(1) Supplemntary Plumpy/Nutriset; in cm
(2) Whey RUSF; over the entire duration of study participation in cm
(3) Soy/Peanut RUSF; over the entire duration of study participation in cm
(4) cm

Supplementary Figure S15. Forest plot of enhanced FBF vs LNS - Outcome: Weight gain (subgroup analysis by dose)

Study or Subgroup	Enhanced FBFs Mean	Enhanced FBFs SD	Enhanced FBFs Total	LNS Mean	LNS SD	LNS Total	Weight	Mean Difference IV, Random, 95% CI
**11.5.1 Medium fixed dose**								
Ackatia-Armah 2015 (1)	1.77	1.12	342	1.97	1.24	335	38.9%	-0.20 [-0.38, -0.02]
**Subtotal (95% CI)**			**342**			**335**	**38.9%**	**-0.20 [-0.38, -0.02]**
Heterogeneity: Not applicable								
Test for overall effect: Z = 2.20 (P = 0.03)								
**11.5.2 High weight dependent dose**								
LaGrone 2012 (2)	3.1	2.4	444	3.4	2.6	906	27.1%	-0.30 [-0.58, -0.02]
LaGrone 2012 (3)	3.1	2.4	444	3.6	2.8	918	26.4%	-0.50 [-0.79, -0.21]
**Subtotal (95% CI)**			**888**			**1824**	**53.6%**	**-0.40 [-0.60, -0.20]**
Heterogeneity: Tau² = 0.00; Chi² = 0.95, df = 1 (P = 0.33); I² = 0%								
Test for overall effect: Z = 3.88 (P = 0.0001)								
**11.5.3 Low weight dependent dose**								
Medoua 2016 (4)	1.83	1.64	41	2.71	1.54	40	7.5%	-0.88 [-1.57, -0.19]
**Subtotal (95% CI)**			**41**			**40**	**7.5%**	**-0.88 [-1.57, -0.19]**
Heterogeneity: Not applicable								
Test for overall effect: Z = 2.49 (P = 0.01)								
**Total (95% CI)**			**1271**			**2199**	**100.0%**	**-0.36 [-0.56, -0.15]**
Heterogeneity: Tau² = 0.02; Chi² = 5.75, df = 3 (P = 0.12); I² = 48%								
Test for overall effect: Z = 3.42 (P = 0.0006)								
Test for subgroup differences: Chi² = 4.80, df = 2 (P = 0.09), I² = 58.3%								

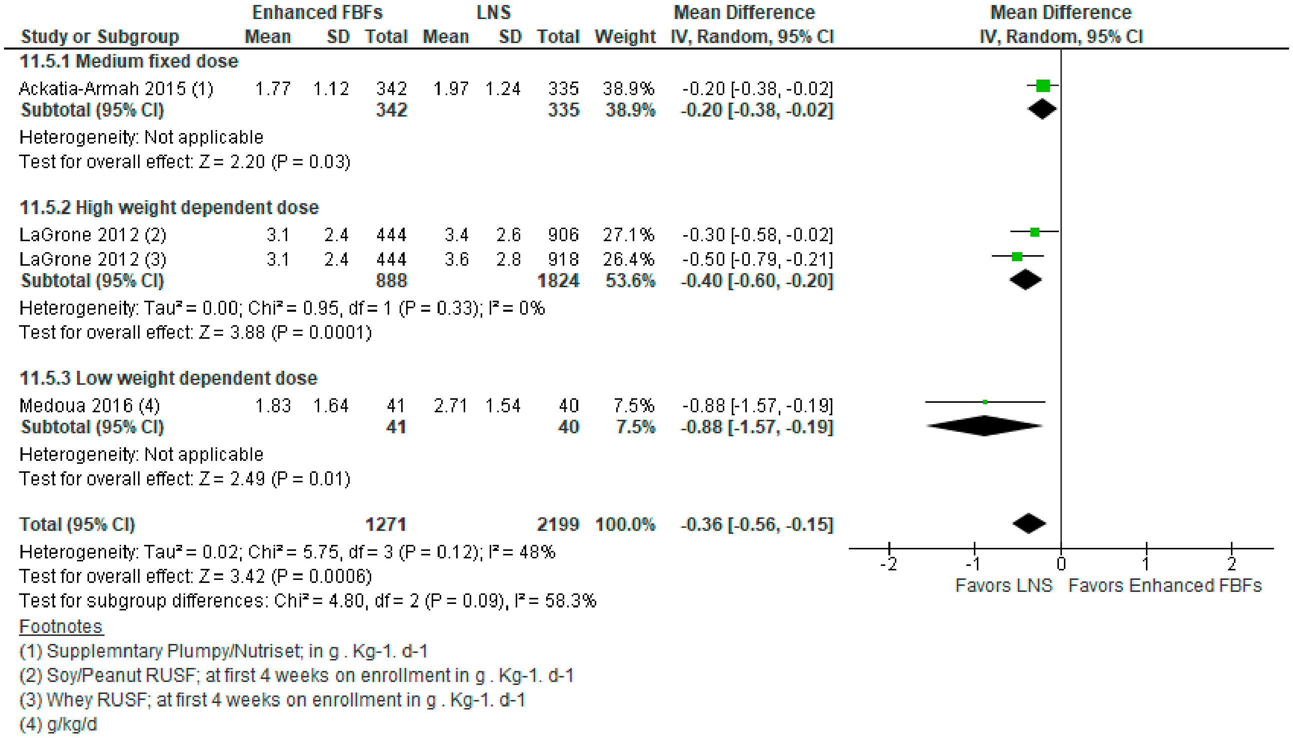

Footnotes
(1) Supplemntary Plumpy/Nutriset; in g . Kg-1. d-1
(2) Soy/Peanut RUSF; at first 4 weeks on enrollment in g . Kg-1. d-1
(3) Whey RUSF; at first 4 weeks on enrollment in g . Kg-1. d-1
(4) g/kg/d

Supplementary Figure S16. Forest plot of enhanced FBF vs LNS - Outcome: Height gain (subgroup analysis by dose)

Study or Subgroup	Enhanced FBFs Mean	Enhanced FBFs SD	Enhanced FBFs Total	LNS Mean	LNS SD	LNS Total	Weight	Mean Difference IV, Random, 95% CI
**11.6.1 Medium fixed dose**								
Ackatia-Armah 2015 (1)	2.9	1.41	342	2.9	1.37	335	68.5%	0.00 [-0.21, 0.21]
**Subtotal (95% CI)**			**342**			**335**	**68.5%**	**0.00 [-0.21, 0.21]**
Heterogeneity: Not applicable								
Test for overall effect: Z = 0.00 (P = 1.00)								
**11.6.2 High weight dependent dose**								
LaGrone 2012 (2)	1.1	3.86	444	1.09	3.69	906	16.1%	0.01 [-0.42, 0.44]
LaGrone 2012 (3)	1.1	3.86	444	1.26	3.95	918	15.5%	-0.16 [-0.60, 0.28]
**Subtotal (95% CI)**			**888**			**1824**	**31.5%**	**-0.07 [-0.38, 0.24]**
Heterogeneity: Tau² = 0.00; Chi² = 0.29, df = 1 (P = 0.59); I² = 0%								
Test for overall effect: Z = 0.47 (P = 0.64)								
**Total (95% CI)**			**1230**			**2159**	**100.0%**	**-0.02 [-0.20, 0.15]**
Heterogeneity: Tau² = 0.00; Chi² = 0.44, df = 2 (P = 0.80); I² = 0%								
Test for overall effect: Z = 0.26 (P = 0.79)								
Test for subgroup differences: Chi² = 0.15, df = 1 (P = 0.70), I² = 0%								

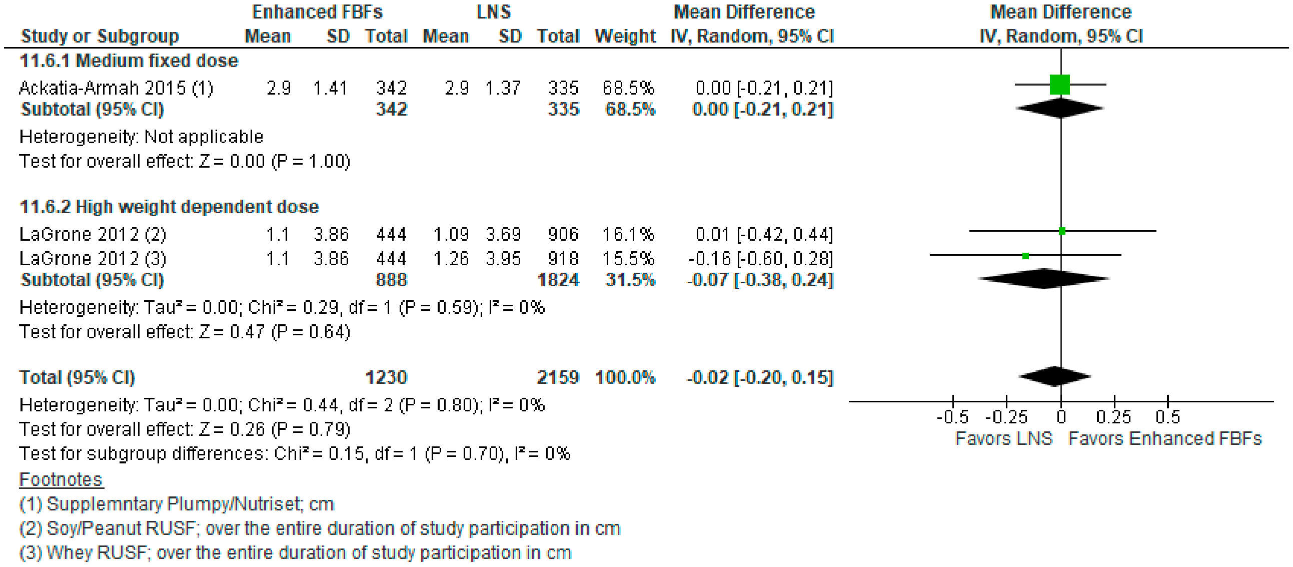

Footnotes
(1) Supplemntary Plumpy/Nutriset; cm
(2) Soy/Peanut RUSF; over the entire duration of study participation in cm
(3) Whey RUSF; over the entire duration of study participation in cm

Supplementary Figure S17. Forest plot of enhanced FBF vs LNS - Outcome: Non-response (subgroup analysis by dose)

Study or Subgroup	Enhanced FBFs Events	Enhanced FBFs Total	LNS Events	LNS Total	Weight	Risk Ratio M-H, Random, 95% CI
**11.7.1 Medium fixed dose**						
Ackatia-Armah 2015 (1)	115	342	78	335	27.1%	1.44 [1.13, 1.84]
Fabiansen 2017 (2)	192	800	243	809	29.9%	0.80 [0.68, 0.94]
**Subtotal (95% CI)**		**1142**		**1144**	**57.0%**	**1.07 [0.60, 1.90]**
Total events	307		321			
Heterogeneity: Tau² = 0.16; Chi² = 15.61, df = 1 (P < 0.0001); I² = 94%						
Test for overall effect: Z = 0.22 (P = 0.83)						
**11.7.2 Low fixed dose**						
Nikièma 2014 (3)	75	675	72	694	24.8%	1.07 [0.79, 1.45]
**Subtotal (95% CI)**		**675**		**694**	**24.8%**	**1.07 [0.79, 1.45]**
Total events	75		72			
Heterogeneity: Not applicable						
Test for overall effect: Z = 0.44 (P = 0.66)						
**11.7.3 High weight dependent dose**						
LaGrone 2012 (4)	4	444	5	906	4.8%	1.63 [0.44, 6.05]
LaGrone 2012 (5)	4	444	8	918	5.6%	1.03 [0.31, 3.41]
**Subtotal (95% CI)**		**888**		**1824**	**10.4%**	**1.27 [0.53, 3.08]**
Total events	8		13			
Heterogeneity: Tau² = 0.00; Chi² = 0.26, df = 1 (P = 0.61); I² = 0%						
Test for overall effect: Z = 0.53 (P = 0.59)						
**11.7.4 Low weight dependent dose**						
Medoua 2016 (6)	8	41	6	40	7.8%	1.30 [0.50, 3.41]
**Subtotal (95% CI)**		**41**		**40**	**7.8%**	**1.30 [0.50, 3.41]**
Total events	8		6			
Heterogeneity: Not applicable						
Test for overall effect: Z = 0.53 (P = 0.59)						
**Total (95% CI)**		**2746**		**3702**	**100.0%**	**1.10 [0.81, 1.50]**
Total events	398		412			
Heterogeneity: Tau² = 0.08; Chi² = 16.98, df = 5 (P = 0.005); I² = 71%						
Test for overall effect: Z = 0.60 (P = 0.55)						
Test for subgroup differences: Chi² = 0.26, df = 3 (P = 0.97), I² = 0%						

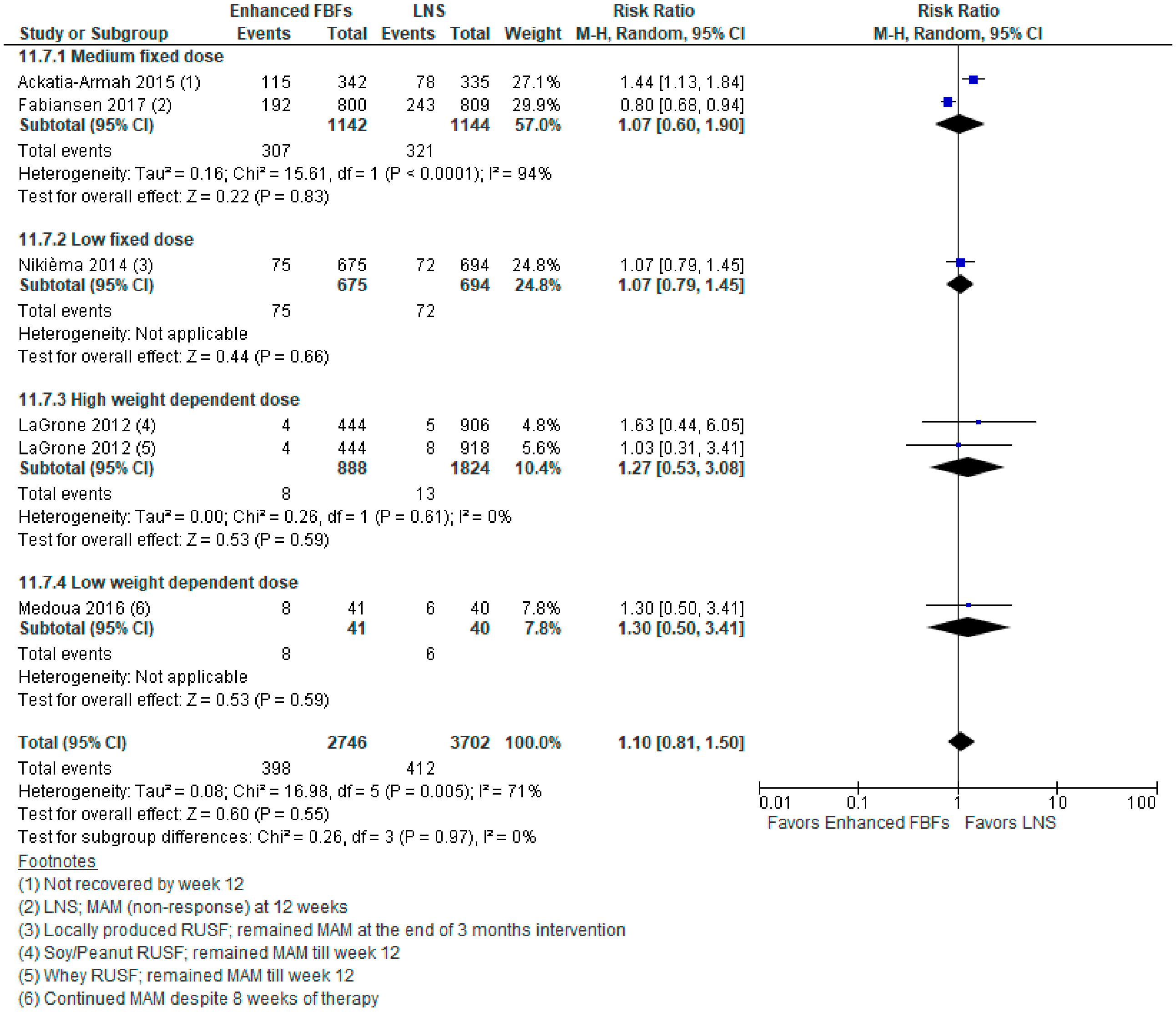

Footnotes
(1) Not recovered by week 12
(2) LNS; MAM (non-response) at 12 weeks
(3) Locally produced RUSF; remained MAM at the end of 3 months intervention
(4) Soy/Peanut RUSF; remained MAM till week 12
(5) Whey RUSF; remained MAM till week 12
(6) Continued MAM despite 8 weeks of therapy

Supplementary Figure S18. Forest plot of enhanced FBF vs LNS - Outcome: Time to recovery (subgroup analysis by dose)

Study or Subgroup	Enhanced FBFs Mean	SD	Total	LNS Mean	SD	Total	Weight	Mean Difference IV, Random, 95% CI
**11.8.1 Medium fixed dose**								
Ackatia-Armah 2015 (1)	49	17.22	209	41.3	11.2	245	20.0%	7.70 [4.98, 10.42]
**Subtotal (95% CI)**			**209**			**245**	**20.0%**	**7.70 [4.98, 10.42]**
Heterogeneity: Not applicable								
Test for overall effect: Z = 5.54 (P < 0.00001)								
**11.8.2 Low fixed dose**								
Nikièma 2014 (2)	29.4	39.9	503	36.4	50.4	515	13.8%	-7.00 [-12.58, -1.42]
**Subtotal (95% CI)**			**503**			**515**	**13.8%**	**-7.00 [-12.58, -1.42]**
Heterogeneity: Not applicable								
Test for overall effect: Z = 2.46 (P = 0.01)								
**11.8.3 High weight dependent dose**								
LaGrone 2012 (3)	24.9	17.5	444	22.5	14.2	906	21.6%	2.40 [0.53, 4.27]
LaGrone 2012 (4)	24.9	17.5	444	22.6	15	918	21.6%	2.30 [0.40, 4.20]
**Subtotal (95% CI)**			**888**			**1824**	**43.2%**	**2.35 [1.02, 3.68]**
Heterogeneity: Tau² = 0.00; Chi² = 0.01, df = 1 (P = 0.94); I² = 0%								
Test for overall effect: Z = 3.46 (P = 0.0005)								
**11.8.4 Low weight dependent dose**								
Medoua 2016 (5)	51.16	1.45	41	44.17	1.87	40	23.0%	6.99 [6.26, 7.72]
**Subtotal (95% CI)**			**41**			**40**	**23.0%**	**6.99 [6.26, 7.72]**
Heterogeneity: Not applicable								
Test for overall effect: Z = 18.77 (P < 0.00001)								
**Total (95% CI)**			**1641**			**2624**	**100.0%**	**3.20 [-0.06, 6.45]**
Heterogeneity: Tau² = 11.84; Chi² = 58.06, df = 4 (P < 0.00001); I² = 93%								
Test for overall effect: Z = 1.92 (P = 0.05)								
Test for subgroup differences: Chi² = 58.06, df = 3 (P < 0.00001), I² = 94.8%								

Mean Difference
IV, Random, 95% CI

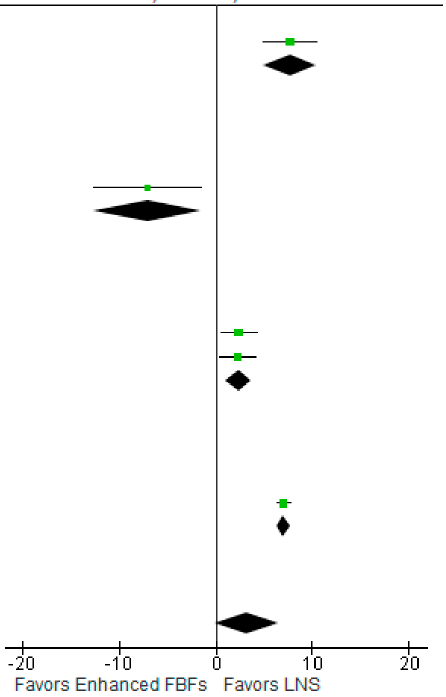

Footnotes
(1) CSB++; in days
(2) Locally produced RUSF; in days
(3) Soy/Peanut RUSF; days
(4) Whey RUSF; days
(5) days

Supplementary Figure S19. Forest plot of enhanced FBF vs LNS - Outcome: Recovery (subgroup analysis by treatment length)

	Enhanced FBFs		LNS			Risk Ratio
**Study or Subgroup**	**Events**	**Total**	**Events**	**Total**	**Weight**	**M-H, Random, 95% CI**
**12.1.1 By 8 weeks**						
Medoua 2016 (1)	30	41	34	40	2.6%	0.86 [0.69, 1.08]
**Subtotal (95% CI)**		**41**		**40**	**2.6%**	**0.86 [0.69, 1.08]**
Total events	30		34			
Heterogeneity: Not applicable						
Test for overall effect: Z = 1.30 (P = 0.19)						
**12.1.2 By 12 weeks**						
Ackatia-Armah 2015 (2)	209	342	245	335	8.5%	0.84 [0.75, 0.93]
Fabiansen 2017 (3)	501	809	552	800	13.3%	0.90 [0.84, 0.96]
Griswold 2021 (4)	360	579	159	256	7.7%	1.00 [0.89, 1.12]
Griswold 2021 (5)	415	663	159	256	7.9%	1.01 [0.90, 1.13]
Griswold 2021 (6)	424	663	159	256	8.0%	1.03 [0.92, 1.15]
LaGrone 2012 (7)	382	444	807	918	18.5%	0.98 [0.94, 1.02]
LaGrone 2012 (8)	381	444	795	906	18.4%	0.98 [0.93, 1.02]
Nikièma 2014 (9)	503	675	515	694	15.0%	1.00 [0.94, 1.07]
**Subtotal (95% CI)**		**4619**		**4421**	**97.4%**	**0.97 [0.93, 1.00]**
Total events	3175		3391			
Heterogeneity: Tau² = 0.00; Chi² = 15.62, df = 7 (P = 0.03); I² = 55%						
Test for overall effect: Z = 1.76 (P = 0.08)						
**Total (95% CI)**		**4660**		**4461**	**100.0%**	**0.96 [0.93, 1.00]**
Total events	3205		3425			
Heterogeneity: Tau² = 0.00; Chi² = 16.67, df = 8 (P = 0.03); I² = 52%						
Test for overall effect: Z = 1.94 (P = 0.05)						
Test for subgroup differences: Chi² = 0.96, df = 1 (P = 0.33), I² = 0%						

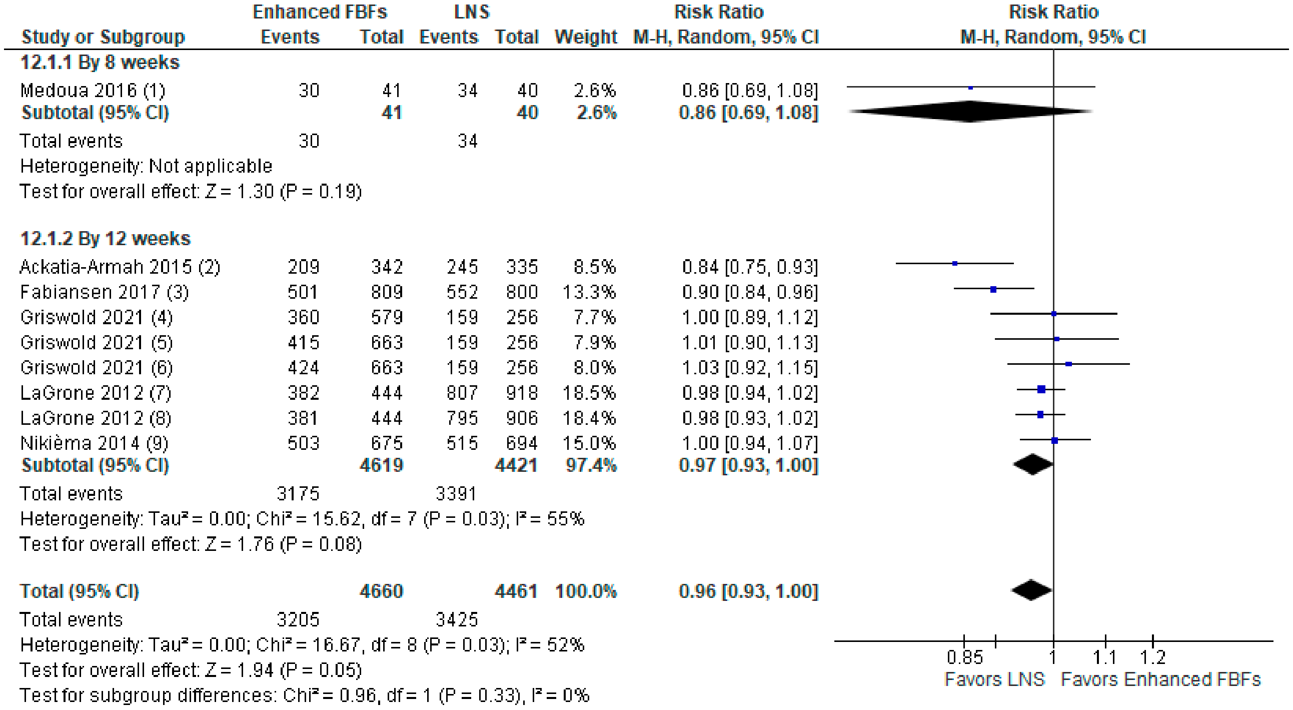

Footnotes
(1) RUSF produced by using pre-cooked soya and corn flours, peanut paste, sugar, soya oil and a premix containing concentrated minerals and...
(2) Supplemntary Plumpy/Nutriset; sustained recovery from MAM, defined as WLZ >-2.0 and MUAC >12.5 cm for >=2 follow-up visits
(3) LNS, factorial trial including 12 different products; recovery at 12 weeks (WHZ >= –2 and MUAC >= 125 mm)
(4) CSWB w/oil; recovery rate defined as achieving MUAC ≥12.5 cm by the seventh visit and no bipedal edema
(5) SC+ A, Super Cereal Plus with amylase; recovery rate defined as achieving MUAC ≥12.5 cm by the seventh visit and no bipedal edema
(6) CSB+w/oil; recovery rate defined as achieving MUAC ≥12.5 cm by the seventh visit and no bipedal edema
(7) Whey RUSF; recovered when they reached a WHZ >= -2
(8) Soy/Peanut RUSF; recovered when they reached a WHZ >= -2
(9) Locally produced RUSF; recovery rate defined as WHZ ≥ -2 SD

Supplementary Figure S20. Forest plot of enhanced FBF vs LNS - Outcome: Deterioration to severe wasting (subgroup analysis by treatment length)

Study or Subgroup	Enhanced FBFs Events	Enhanced FBFs Total	LNS Events	LNS Total	Weight	Risk Ratio M-H, Random, 95% CI
**12.2.1 By 8 weeks**						
Medoua 2016 (1)	2	41	1	40	0.6%	1.95 [0.18, 20.68]
**Subtotal (95% CI)**		**41**		**40**	**0.6%**	**1.95 [0.18, 20.68]**
Total events	2		1			
Heterogeneity: Not applicable						
Test for overall effect: Z = 0.56 (P = 0.58)						
**12.2.2 By 12 weeks**						
Fabiansen 2017 (2)	70	800	59	809	14.6%	1.20 [0.86, 1.67]
Griswold 2021 (3)	116	663	55	256	16.7%	0.81 [0.61, 1.08]
Griswold 2021 (4)	99	579	55	256	16.3%	0.80 [0.59, 1.07]
Griswold 2021 (5)	120	663	55	256	16.8%	0.84 [0.63, 1.12]
LaGrone 2012 (6)	29	444	47	906	10.5%	1.26 [0.80, 1.97]
LaGrone 2012 (7)	30	444	39	918	10.1%	1.59 [1.00, 2.53]
Nikièma 2014 (8)	67	675	57	694	14.4%	1.21 [0.86, 1.69]
**Subtotal (95% CI)**		**4268**		**4095**	**99.4%**	**1.03 [0.85, 1.24]**
Total events	531		367			
Heterogeneity: $Tau^2 = 0.03$; $Chi^2 = 12.98$, df = 6 (P = 0.04); $I^2 = 54\%$						
Test for overall effect: Z = 0.28 (P = 0.78)						
**Total (95% CI)**		**4309**		**4135**	**100.0%**	**1.03 [0.86, 1.23]**
Total events	533		368			
Heterogeneity: $Tau^2 = 0.03$; $Chi^2 = 13.30$, df = 7 (P = 0.07); $I^2 = 47\%$						
Test for overall effect: Z = 0.30 (P = 0.76)						
Test for subgroup differences: $Chi^2 = 0.28$, df = 1 (P = 0.60), $I^2 = 0\%$						

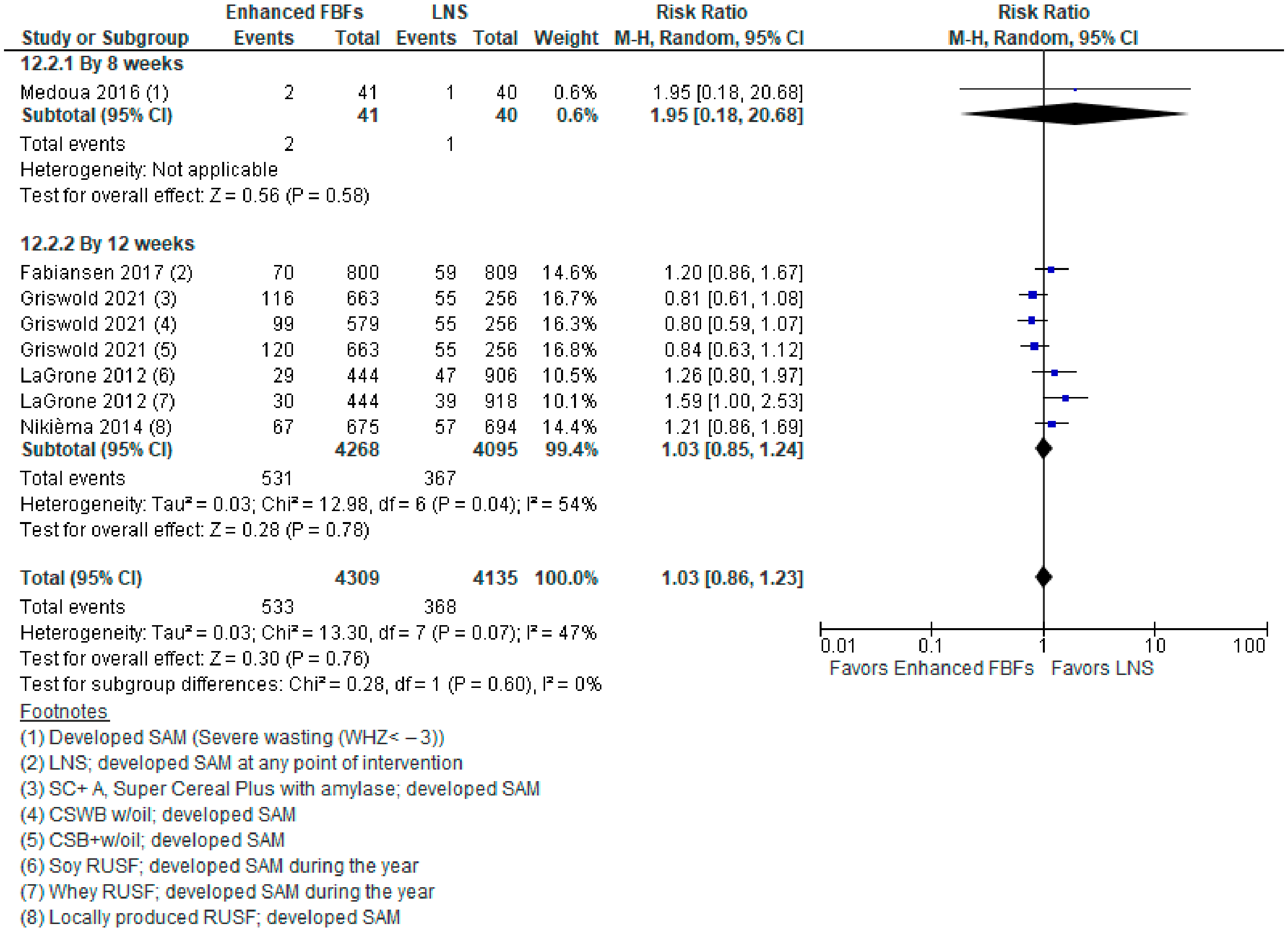

Footnotes
(1) Developed SAM (Severe wasting (WHZ< −3))
(2) LNS; developed SAM at any point of intervention
(3) SC+ A, Super Cereal Plus with amylase; developed SAM
(4) CSWB w/oil; developed SAM
(5) CSB+w/oil; developed SAM
(6) Soy RUSF; developed SAM during the year
(7) Whey RUSF; developed SAM during the year
(8) Locally produced RUSF; developed SAM

Supplementary Figure S21. Forest plot of enhanced FBF vs LNS - Outcome: WHZ (subgroup analysis by treatment length)

Study or Subgroup	Enhanced FBFs Mean	Enhanced FBFs SD	Enhanced FBFs Total	LNS Mean	LNS SD	LNS Total	Weight	Mean Difference IV, Random, 95% CI
**12.3.1 By 8 weeks**								
Medoua 2016 (1)	-1.58	0.89	41	-1.34	0.7	40	2.0%	-0.24 [-0.59, 0.11]
**Subtotal (95% CI)**			**41**			**40**	**2.0%**	**-0.24 [-0.59, 0.11]**
Heterogeneity: Not applicable								
Test for overall effect: Z = 1.35 (P = 0.18)								
**12.3.2 By 12 weeks**								
Ackatia-Armah 2015 (2)	0.76	1.12	342	0.94	1.05	335	9.1%	-0.18 [-0.34, -0.02]
LaGrone 2012 (3)	-1.68	0.67	444	-1.61	0.63	906	43.7%	-0.07 [-0.14, 0.00]
LaGrone 2012 (4)	-1.68	0.67	444	-1.59	0.6	918	45.2%	-0.09 [-0.16, -0.02]
**Subtotal (95% CI)**			**1230**			**2159**	**98.0%**	**-0.09 [-0.14, -0.04]**
Heterogeneity: $Tau^2 = 0.00$; $Chi^2 = 1.44$, df = 2 (P = 0.49); $I^2 = 0\%$								
Test for overall effect: Z = 3.52 (P = 0.0004)								
**Total (95% CI)**			**1271**			**2199**	**100.0%**	**-0.09 [-0.14, -0.04]**
Heterogeneity: $Tau^2 = 0.00$; $Chi^2 = 2.14$, df = 3 (P = 0.54); $I^2 = 0\%$								
Test for overall effect: Z = 3.67 (P = 0.0002)								
Test for subgroup differences: $Chi^2 = 0.70$, df = 1 (P = 0.40), $I^2 = 0\%$								

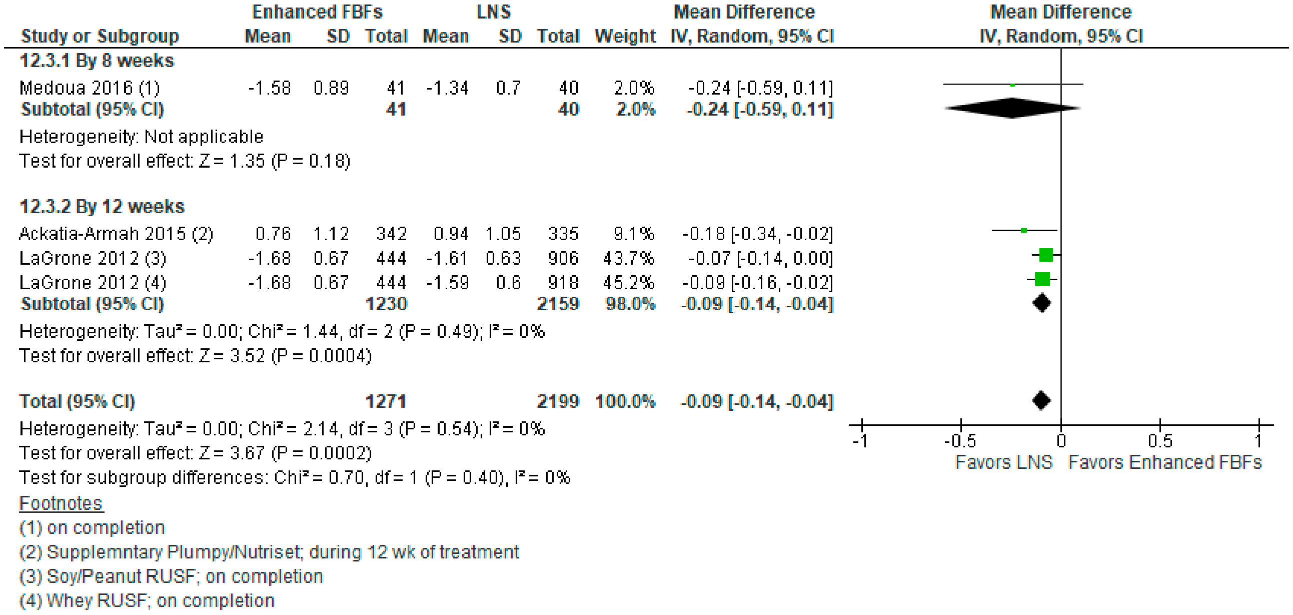

Footnotes
(1) on completion
(2) Supplemntary Plumpy/Nutriset; during 12 wk of treatment
(3) Soy/Peanut RUSF; on completion
(4) Whey RUSF; on completion

Supplementary Figure S22. Forest plot of enhanced FBF vs LNS - Outcome: MUAC gain (subgroup analysis by treatment length)

Study or Subgroup	Enhanced FBFs Mean	Enhanced FBFs SD	Enhanced FBFs Total	LNS Mean	LNS SD	LNS Total	Weight	Mean Difference IV, Random, 95% CI
**12.4.1 By 8 weeks**								
Medoua 2016 (1)	0.56	1.06	41	0.78	0.67	40	19.9%	-0.22 [-0.61, 0.17]
**Subtotal (95% CI)**			**41**			**40**	**19.9%**	**-0.22 [-0.61, 0.17]**
Heterogeneity: Not applicable								
Test for overall effect: Z = 1.12 (P = 0.26)								
**12.4.2 By 12 weeks**								
Ackatia-Armah 2015 (2)	0.9	0.94	342	1.1	0.91	335	41.0%	-0.20 [-0.34, -0.06]
LaGrone 2012 (3)	1.09	3.36	444	1.09	3.6	906	19.6%	0.00 [-0.39, 0.39]
LaGrone 2012 (4)	1.09	3.36	444	1.76	3.69	918	19.5%	-0.67 [-1.06, -0.28]
**Subtotal (95% CI)**			**1230**			**2159**	**80.1%**	**-0.27 [-0.58, 0.04]**
Heterogeneity: Tau² = 0.05; Chi² = 6.32, df = 2 (P = 0.04); I² = 68%								
Test for overall effect: Z = 1.73 (P = 0.08)								
**Total (95% CI)**			**1271**			**2199**	**100.0%**	**-0.26 [-0.48, -0.03]**
Heterogeneity: Tau² = 0.03; Chi² = 6.32, df = 3 (P = 0.10); I² = 53%								
Test for overall effect: Z = 2.24 (P = 0.02)								
Test for subgroup differences: Chi² = 0.05, df = 1 (P = 0.83), I² = 0%								

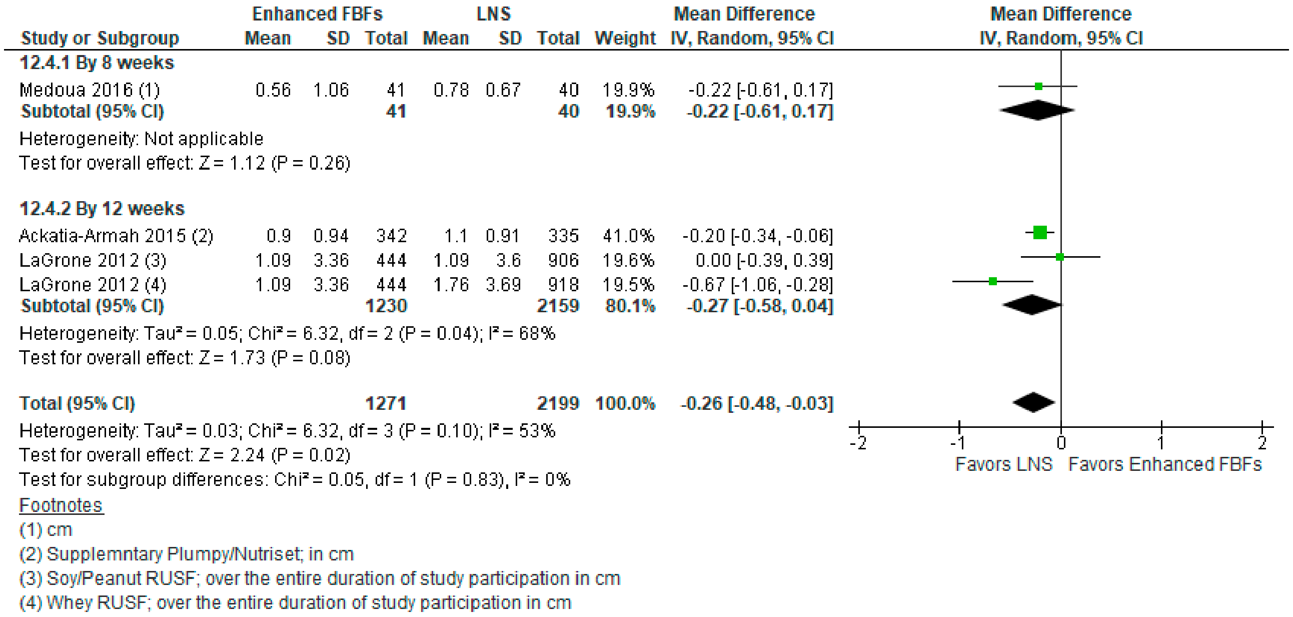

Footnotes
(1) cm
(2) Supplemntary Plumpy/Nutriset; in cm
(3) Soy/Peanut RUSF; over the entire duration of study participation in cm
(4) Whey RUSF; over the entire duration of study participation in cm

Figure S23. Forest plot of enhanced FBF vs LNS - Outcome: Weight gain (subgroup analysis by treatment length)

Study or Subgroup	Enhanced FBFs Mean	Enhanced FBFs SD	Enhanced FBFs Total	LNS Mean	LNS SD	LNS Total	Weight	Mean Difference IV, Random, 95% CI
**12.5.1 By 8 weeks**								
Medoua 2016 (1)	1.83	1.64	41	2.71	1.54	40	7.5%	-0.88 [-1.57, -0.19]
**Subtotal (95% CI)**			**41**			**40**	**7.5%**	**-0.88 [-1.57, -0.19]**
Heterogeneity: Not applicable								
Test for overall effect: Z = 2.49 (P = 0.01)								
**12.5.2 By 12 weeks**								
Ackatia-Armah 2015 (2)	1.77	1.12	342	1.97	1.24	335	38.9%	-0.20 [-0.38, -0.02]
LaGrone 2012 (3)	3.1	2.4	444	3.4	2.6	906	27.1%	-0.30 [-0.58, -0.02]
LaGrone 2012 (4)	3.1	2.4	444	3.6	2.8	918	26.4%	-0.50 [-0.79, -0.21]
**Subtotal (95% CI)**			**1230**			**2159**	**92.5%**	**-0.30 [-0.48, -0.13]**
Heterogeneity: Tau² = 0.01; Chi² = 3.03, df = 2 (P = 0.22); I² = 34%								
Test for overall effect: Z = 3.48 (P = 0.0005)								
**Total (95% CI)**			**1271**			**2199**	**100.0%**	**-0.36 [-0.56, -0.15]**
Heterogeneity: Tau² = 0.02; Chi² = 5.75, df = 3 (P = 0.12); I² = 48%								
Test for overall effect: Z = 3.42 (P = 0.0006)								
Test for subgroup differences: Chi² = 2.49, df = 1 (P = 0.11), I² = 59.9%								

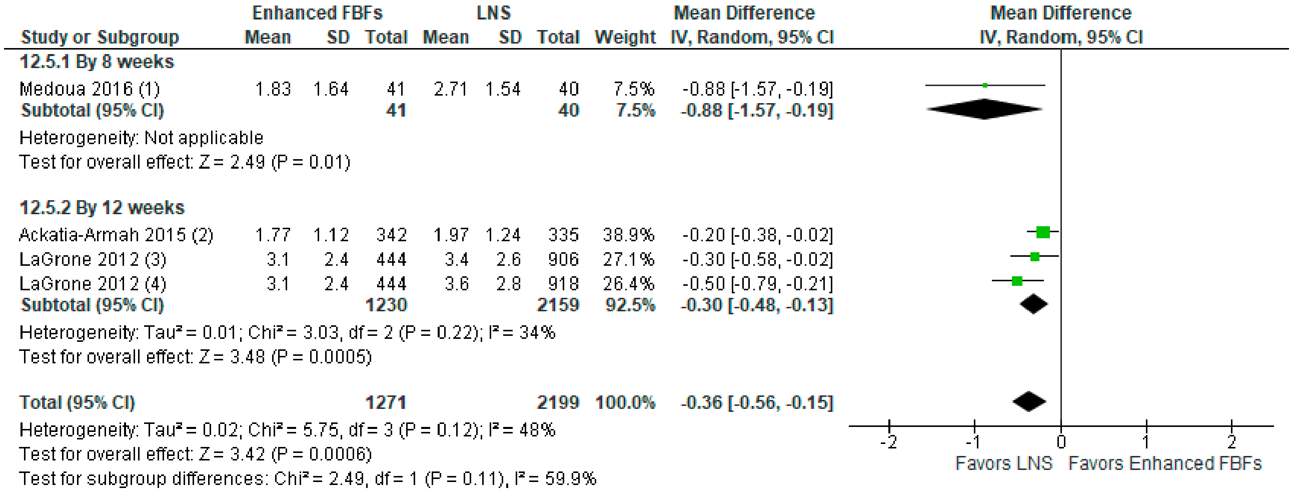

Footnotes
(1) g/kg/d
(2) Supplemntary Plumpy/Nutriset; in g . Kg-1. d-1
(3) Soy/Peanut RUSF; at first 4 weeks on enrollment in g . Kg-1. d-1
(4) Whey RUSF; at first 4 weeks on enrollment in g . Kg-1. d-1

Supplementary Figure S24. Forest plot of enhanced FBF vs LNS - Outcome: Non-response (subgroup analysis by treatment length)

Study or Subgroup	Enhanced FBFs Events	Enhanced FBFs Total	LNS Events	LNS Total	Weight	Risk Ratio M-H, Random, 95% CI
**12.6.1 By 8 weeks**						
Medoua 2016 (1)	8	41	6	40	7.8%	1.30 [0.50, 3.41]
**Subtotal (95% CI)**		**41**		**40**	**7.8%**	**1.30 [0.50, 3.41]**
Total events	8		6			
Heterogeneity: Not applicable						
Test for overall effect: Z = 0.53 (P = 0.59)						
**12.6.2 By 12 weeks**						
Ackatia-Armah 2015 (2)	115	342	78	335	27.1%	1.44 [1.13, 1.84]
Fabiansen 2017 (3)	192	800	243	809	29.9%	0.80 [0.68, 0.94]
LaGrone 2012 (4)	4	444	8	918	5.6%	1.03 [0.31, 3.41]
LaGrone 2012 (5)	4	444	5	906	4.8%	1.63 [0.44, 6.05]
Nikièma 2014 (6)	75	675	72	694	24.8%	1.07 [0.79, 1.45]
**Subtotal (95% CI)**		**2705**		**3662**	**92.2%**	**1.09 [0.78, 1.52]**
Total events	390		406			
Heterogeneity: Tau² = 0.08; Chi² = 16.65, df = 4 (P = 0.002); I² = 76%						
Test for overall effect: Z = 0.49 (P = 0.63)						
**Total (95% CI)**		**2746**		**3702**	**100.0%**	**1.10 [0.81, 1.50]**
Total events	398		412			
Heterogeneity: Tau² = 0.08; Chi² = 16.98, df = 5 (P = 0.005); I² = 71%						
Test for overall effect: Z = 0.60 (P = 0.55)						
Test for subgroup differences: Chi² = 0.12, df = 1 (P = 0.73), I² = 0%						

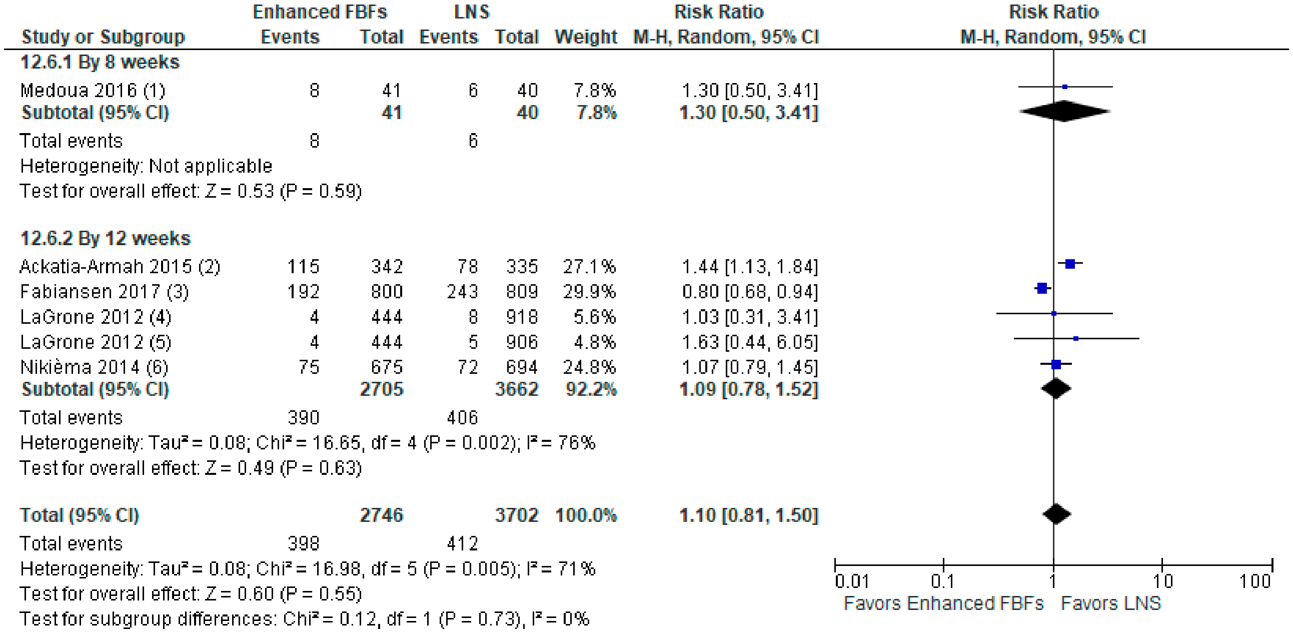

Footnotes
(1) Continued MAM despite 8 weeks of therapy
(2) Not recovered by week 12
(3) LNS; MAM (non-response) at 12 weeks
(4) Whey RUSF; remained MAM till week 12
(5) Soy/Peanut RUSF; remained MAM till week 12
(6) Locally produced RUSF; remained MAM at the end of 3 months intervention

Supplementary Figure S25. Forest plot of enhanced FBF vs LNS - Outcome: Time to recovery (subgroup analysis by treatment length)

Study or Subgroup	Enhanced FBFs Mean	Enhanced FBFs SD	Enhanced FBFs Total	LNS Mean	LNS SD	LNS Total	Weight	Mean Difference IV, Random, 95% CI
**12.7.1 By 8 weeks**								
Medoua 2016 (1)	51.16	1.45	41	44.17	1.87	40	23.0%	6.99 [6.26, 7.72]
**Subtotal (95% CI)**			**41**			**40**	**23.0%**	**6.99 [6.26, 7.72]**
Heterogeneity: Not applicable								
Test for overall effect: Z = 18.77 (P < 0.00001)								
**12.7.2 By 12 weeks**								
Ackatia-Armah 2015 (2)	49	17.22	209	41.3	11.2	245	20.0%	7.70 [4.98, 10.42]
LaGrone 2012 (3)	24.9	17.5	444	22.6	15	918	21.6%	2.30 [0.40, 4.20]
LaGrone 2012 (4)	24.9	17.5	444	22.5	14.2	906	21.6%	2.40 [0.53, 4.27]
Nikièma 2014 (5)	29.4	39.9	503	36.4	50.4	515	13.8%	-7.00 [-12.58, -1.42]
**Subtotal (95% CI)**			**1600**			**2584**	**77.0%**	**2.07 [-1.60, 5.75]**
Heterogeneity: Tau² = 11.60; Chi² = 24.69, df = 3 (P < 0.0001); I² = 88%								
Test for overall effect: Z = 1.10 (P = 0.27)								
**Total (95% CI)**			**1641**			**2624**	**100.0%**	**3.20 [-0.06, 6.45]**
Heterogeneity: Tau² = 11.84; Chi² = 58.06, df = 4 (P < 0.00001); I² = 93%								
Test for overall effect: Z = 1.92 (P = 0.05)								
Test for subgroup differences: Chi² = 6.62, df = 1 (P = 0.01), I² = 84.9%								

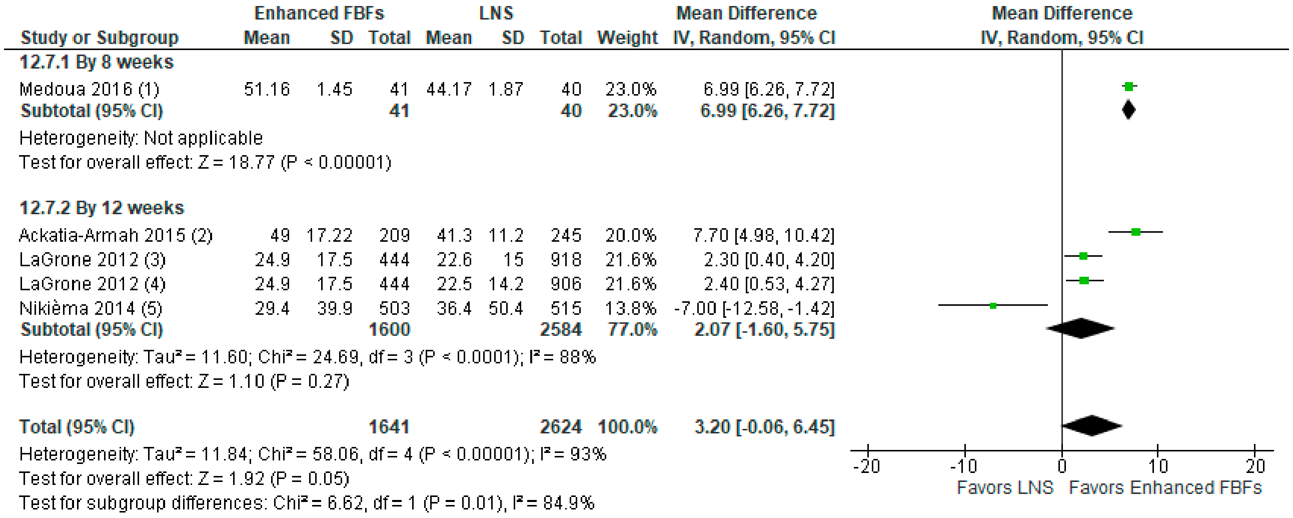

Footnotes
(1) days
(2) CSB++; in days
(3) Whey RUSF; days
(4) Soy/Peanut RUSF; days
(5) Locally produced RUSF; in days

Supplementary Figure S26. Forest plot of CSB compared to LNS - Outcome: Deterioration to severe wasting

Study or Subgroup	CSB Events	CSB Total	LNS Events	LNS Total	Weight	Risk Ratio M-H, Random, 95% CI
Karakochuk 2012 (1)	8	750	6	375	13.7%	0.67 [0.23, 1.91]
Matilsky 2009 (2)	18	224	39	465	28.5%	0.96 [0.56, 1.64]
Matilsky 2009 (3)	17	223	35	450	27.6%	0.98 [0.56, 1.71]
Nackers 2010 (4)	45	236	20	215	30.2%	2.05 [1.25, 3.36]
**Total (95% CI)**		**1433**		**1505**	**100.0%**	**1.15 [0.73, 1.84]**
Total events	88		100			

Heterogeneity: Tau² = 0.12; Chi² = 6.90, df = 3 (P = 0.08); I² = 57%
Test for overall effect: Z = 0.61 (P = 0.54)

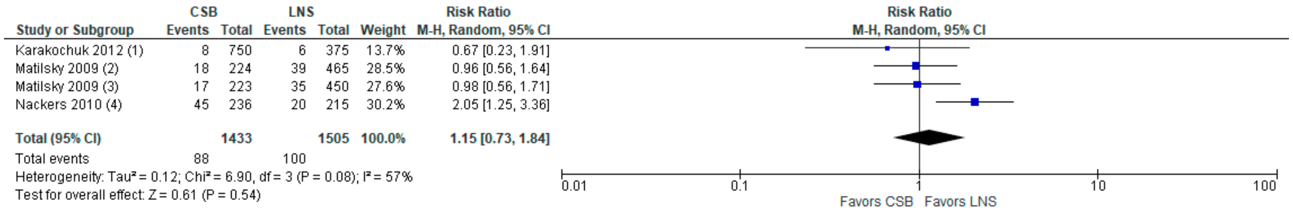

Footnotes
(1) CSB vs Supplementary Plumpy by Nutriset; Children who deteriorated to SAM defined as a WFH <70%, MUAC <110mm and required transfer to inpatient care.
(2) CSB vs Milk/Peanut Fortified Spread; Deterioration to SAM measured by edema only.
(3) CSB vs Soy/Peanut Fortified Spread; Deterioration to SAM measured by edema only.
(4) CSB vs Standard RUTF; Number refers to transfers to the inpatient Therapeutic Feeding Centres which in addition to SAM included medical criteria (severe disease, severe anemia or severe dehydration).

Supplementary Figure S27. Forest plot of CSB compared to LNS - Outcome: WHZ

Study or Subgroup	CSB Mean	CSB SD	CSB Total	LNS Mean	LNS SD	LNS Total	Weight	Mean Difference IV, Random, 95% CI
Matilsky 2009 (1)	-1.8	0.8	223	-1.7	0.7	450	49.8%	-0.10 [-0.22, 0.02]
Matilsky 2009 (2)	-1.8	0.8	224	-1.6	0.7	465	50.2%	-0.20 [-0.32, -0.08]
**Total (95% CI)**			**447**			**915**	**100.0%**	**-0.15 [-0.25, -0.05]**

Heterogeneity: Tau² = 0.00; Chi² = 1.27, df = 1 (P = 0.26); I² = 21%
Test for overall effect: Z = 3.00 (P = 0.003)

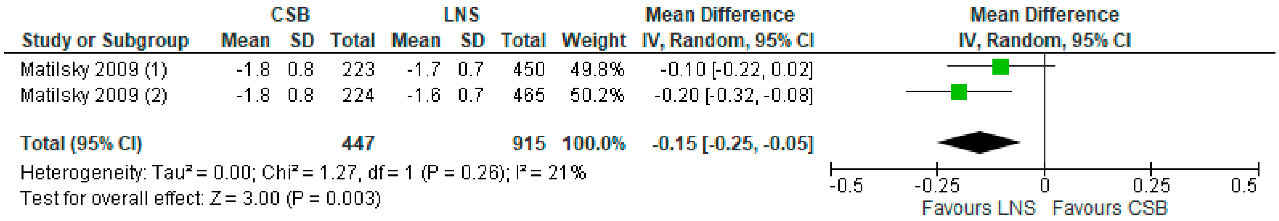

Footnotes
(1) Soy Peanut Fortified Spread; WHZ at discharge (intervention period was upto 8 weeks)
(2) Milk Peanut Fortified Spread; WHZ at discharge (intervention period was upto 8 weeks)

Supplementary Figure S28. Forest plot of CSB compared to LNS - Outcome: HAZ

Study or Subgroup	CSB Mean	CSB SD	CSB Total	LNS Mean	LNS SD	LNS Total	Weight	Mean Difference IV, Random, 95% CI
Matilsky 2009 (1)	-2.8	1.9	223	-2.6	1.5	450	48.2%	-0.20 [-0.49, 0.09]
Matilsky 2009 (2)	-2.8	1.9	224	-2.7	1.3	465	51.8%	-0.10 [-0.38, 0.18]
**Total (95% CI)**			**447**			**915**	**100.0%**	**-0.15 [-0.35, 0.05]**

Heterogeneity: Tau² = 0.00; Chi² = 0.24, df = 1 (P = 0.62); I² = 0%
Test for overall effect: Z = 1.47 (P = 0.14)

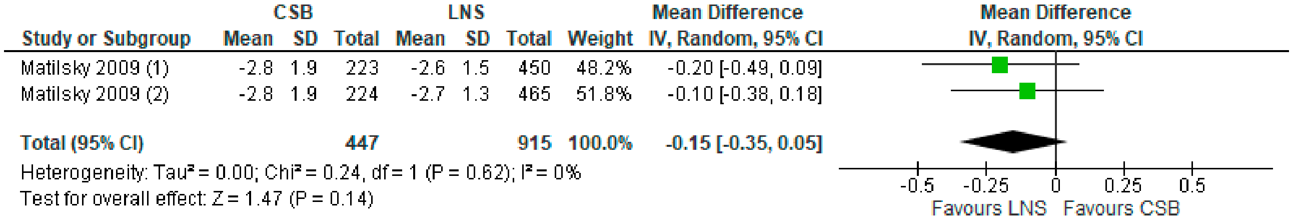

Footnotes
(1) CSB vs Soy/Peanut Fortified Spread; HAZ at discharge (intervention period was up to 8 weeks).
(2) CSB vs Milk/Peanut Fortified Spread; HAZ at discharge (intervention period up to 8 weeks).

Supplementary Figure S29. Forest plot of CSB compared to LNS - Outcome: MUAC gain (mm/day)

Study or Subgroup	CSB Mean	CSB SD	CSB Total	LNS Mean	LNS SD	LNS Total	Weight	Mean Difference IV, Random, 95% CI
Nackers 2010 (1)	0.32	0.24	150	0.37	0.29	162	100.0%	-0.05 [-0.11, 0.01]
**Total (95% CI)**			**150**			**162**	**100.0%**	**-0.05 [-0.11, 0.01]**

Heterogeneity: Not applicable
Test for overall effect: Z = 1.66 (P = 0.10)

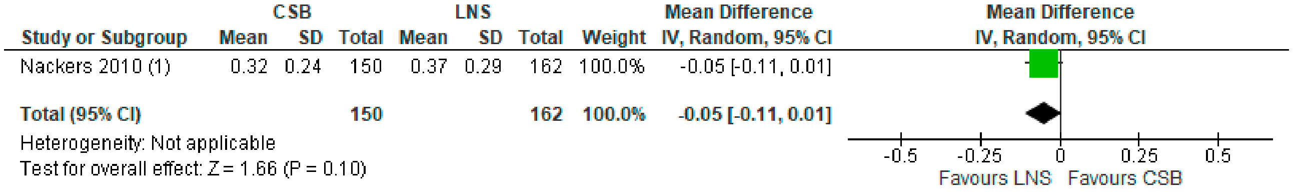

Footnotes
(1) CSB vs standard RUTF; MUAC gain in mm/day up to discharge.

Supplementary Figure S30. Forest plot of CSB compared to LNS - Outcome: Weight gain (g/kg/day)

Study or Subgroup	CSB Mean	CSB SD	CSB Total	LNS Mean	LNS SD	LNS Total	Weight	Mean Difference IV, Random, 95% CI
Nackers 2010 (1)	3.48	4.32	217	5.34	4.15	204	100.0%	-1.86 [-2.67, -1.05]
**Total (95% CI)**			**217**			**204**	**100.0%**	**-1.86 [-2.67, -1.05]**

Heterogeneity: Not applicable
Test for overall effect: Z = 4.51 (P < 0.00001)

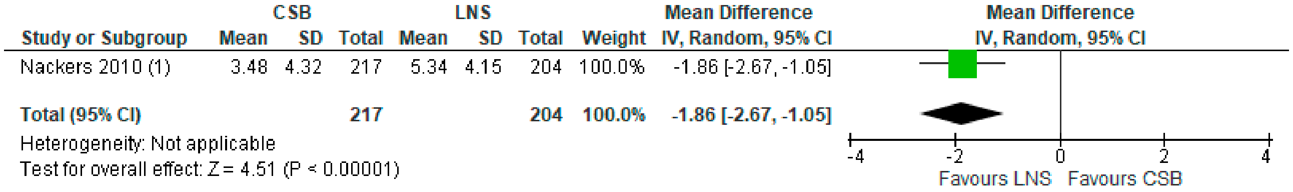

Footnotes
(1) CSB vs Standard RUTF; Weight gain in g/kg/day during the first 2 weeks of treatment.

Supplementary Figure S31. Forest plot of CSB compared to LNS - Outcome: Non-response

Study or Subgroup	CSB Events	CSB Total	LNS Events	LNS Total	Weight	Risk Ratio M-H, Random, 95% CI
Karakochuk 2012 (1)	86	375	216	750	28.1%	0.80 [0.64, 0.99]
Matilsky 2009 (2)	33	223	29	450	24.9%	2.30 [1.43, 3.68]
Matilsky 2009 (3)	34	224	35	465	25.3%	2.02 [1.29, 3.14]
Nackers 2010 (4)	13	215	21	236	21.8%	0.68 [0.35, 1.32]
**Total (95% CI)**		**1037**		**1901**	**100.0%**	**1.27 [0.68, 2.36]**
Total events	166		301			

Heterogeneity: Tau² = 0.35; Chi² = 26.73, df = 3 (P < 0.00001); I² = 89%
Test for overall effect: Z = 0.74 (P = 0.46)

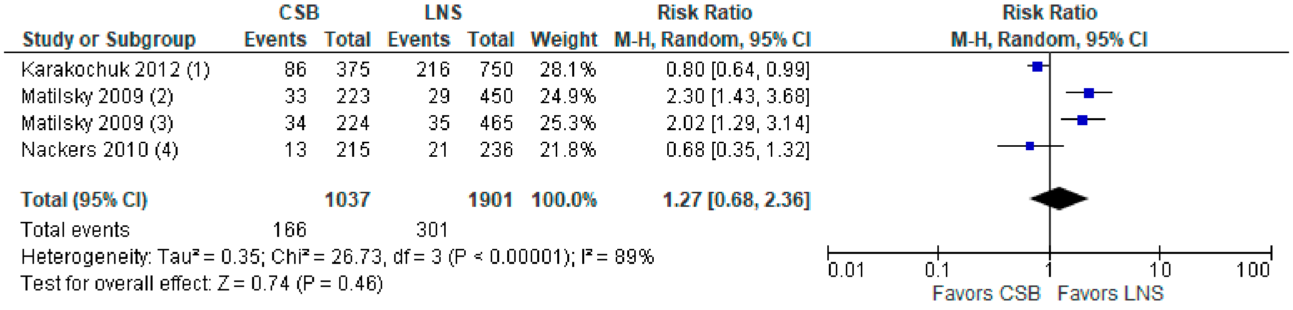

Footnotes
(1) CSB vs Supplementary Plumpy Nutriset; Non-response defined as not reaching a WFH>85% on 2 consecutive visits within 16 weeks.
(2) CSB vs Soy/Peanut Fortified Spread; Non-response defined as not reaching a WHZ> -2 within 8 weeks.
(3) CSB vs Milk/Peanut Fortified Spread; Non-response defined as not reaching a WHZ> -2 within 8 weeks.
(4) CSB vs standard RUTF; Non-response defined as not reaching a WHM%>85% for 2 consecutive weeks within 16 weeks.

Supplementary Figure S32. Forest plot of CSB compared to LNS - Outcome: Time to recovery

Study or Subgroup	CSB Mean	CSB SD	CSB Total	LNS Mean	LNS SD	LNS Total	Weight	Mean Difference IV, Random, 95% CI
Nackers 2010 (1)	8	10.5	152	7.3	10.5	170	100.0%	0.70 [-1.60, 3.00]
**Total (95% CI)**			**152**			**170**	**100.0%**	**0.70 [-1.60, 3.00]**

Heterogeneity: Not applicable
Test for overall effect: Z = 0.60 (P = 0.55)

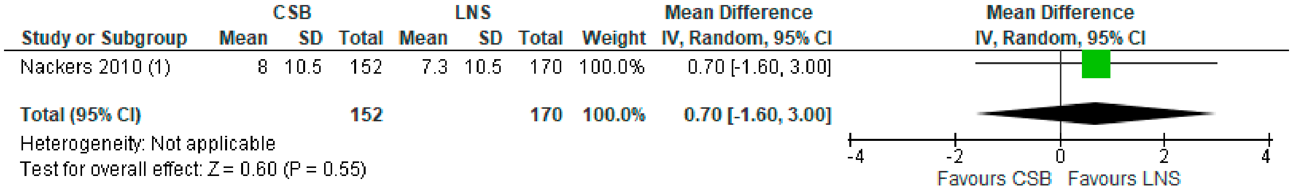

Footnotes
(1) Standard RUTF; length of stay up to discharge (weeks)

Supplementary Figure S33. Forest plot of CSB compared to LNS - Outcome: Relapse

Study or Subgroup	CSB Events	CSB Total	LNS Events	LNS Total	Weight	Risk Ratio M-H, Random, 95% CI
Nackers 2010 (1)	33	152	33	170	100.0%	1.12 [0.73, 1.72]
**Total (95% CI)**		**152**		**170**	**100.0%**	**1.12 [0.73, 1.72]**
Total events	33		33			
Heterogeneity: Not applicable						
Test for overall effect: Z = 0.51 (P = 0.61)						

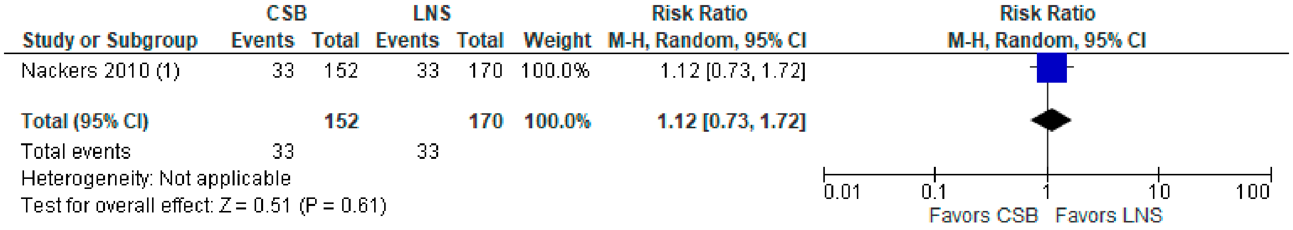

Footnotes
(1) CSB vs Standard RUTF; Relapse to acute malnutrition within six months after discharge

Supplementary Figure S34. Forest plot of CSB vs LNS - Outcome: Recovery rate (subgroup analysis by dose)

Study or Subgroup	CSB Events	CSB Total	LNS Events	LNS Total	Weight	Risk Ratio M-H, Random, 95% CI
**9.1.1 High fixed dose**						
Nackers 2010 (1)	152	236	170	215	16.5%	0.81 [0.72, 0.92]
**Subtotal (95% CI)**		**236**		**215**	**16.5%**	**0.81 [0.72, 0.92]**
Total events	152		170			
Heterogeneity: Not applicable						
Test for overall effect: Z = 3.43 (P = 0.0006)						
**9.1.2 Medium fixed dose**						
Karakochuk 2012 (2)	482	750	265	375	31.8%	0.91 [0.84, 0.99]
**Subtotal (95% CI)**		**750**		**375**	**31.8%**	**0.91 [0.84, 0.99]**
Total events	482		265			
Heterogeneity: Not applicable						
Test for overall effect: Z = 2.21 (P = 0.03)						
**9.1.3 High weight dependent dose**						
Matilsky 2009 (3)	162	224	369	465	25.9%	0.91 [0.83, 1.00]
Matilsky 2009 (4)	161	223	360	450	25.8%	0.90 [0.82, 0.99]
**Subtotal (95% CI)**		**447**		**915**	**51.7%**	**0.91 [0.85, 0.97]**
Total events	323		729			
Heterogeneity: Tau² = 0.00; Chi² = 0.02, df = 1 (P = 0.88); I² = 0%						
Test for overall effect: Z = 2.90 (P = 0.004)						
**Total (95% CI)**		**1433**		**1505**	**100.0%**	**0.89 [0.85, 0.94]**
Total events	957		1164			
Heterogeneity: Tau² = 0.00; Chi² = 2.77, df = 3 (P = 0.43); I² = 0%						
Test for overall effect: Z = 4.72 (P < 0.00001)						
Test for subgroup differences: Chi² = 2.75, df = 2 (P = 0.25), I² = 27.4%						

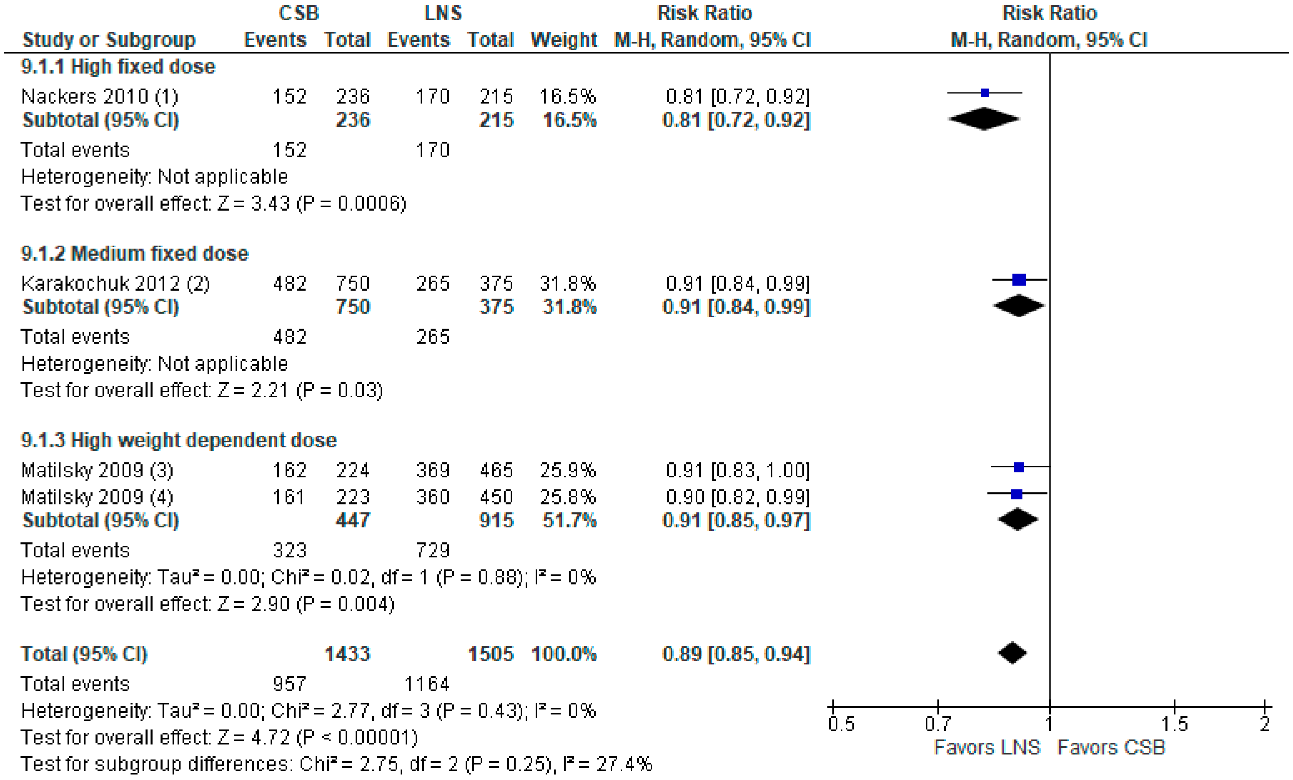

Footnotes
(1) Standard RUTF; WHM% >/= 85% for 2 consecutive weeks
(2) Supplementary plumpy Nutriset; recovery was defined as WFH ≥ 85% on 2 consecutive visits within 16 week duration
(3) Milk/Peanut Fortified Spread; defined as having a WHZ > -2
(4) Soy/Peanut Fortified Spread; defined as having a WHZ > -2

Supplementary Figure S35. Forest plot of CSB vs LNS - Outcome: Deterioration to severe wasting (subgroup analysis by dose)

Study or Subgroup	CSB Events	CSB Total	LNS Events	LNS Total	Weight	Risk Ratio M-H, Random, 95% CI
**9.2.1 High fixed dose**						
Nackers 2010 (1)	45	236	20	215	30.2%	2.05 [1.25, 3.36]
**Subtotal (95% CI)**		**236**		**215**	**30.2%**	**2.05 [1.25, 3.36]**
Total events	45		20			
Heterogeneity: Not applicable						
Test for overall effect: Z = 2.85 (P = 0.004)						
**9.2.2 Medium fixed dose**						
Karakochuk 2012 (2)	8	750	6	375	13.7%	0.67 [0.23, 1.91]
**Subtotal (95% CI)**		**750**		**375**	**13.7%**	**0.67 [0.23, 1.91]**
Total events	8		6			
Heterogeneity: Not applicable						
Test for overall effect: Z = 0.76 (P = 0.45)						
**9.2.3 High weight dependent dose**						
Matilsky 2009 (3)	17	223	35	450	27.6%	0.98 [0.56, 1.71]
Matilsky 2009 (4)	18	224	39	465	28.5%	0.96 [0.56, 1.64]
**Subtotal (95% CI)**		**447**		**915**	**56.1%**	**0.97 [0.66, 1.42]**
Total events	35		74			
Heterogeneity: Tau² = 0.00; Chi² = 0.00, df = 1 (P = 0.95); I² = 0%						
Test for overall effect: Z = 0.16 (P = 0.87)						
**Total (95% CI)**		**1433**		**1505**	**100.0%**	**1.15 [0.73, 1.84]**
Total events	88		100			
Heterogeneity: Tau² = 0.12; Chi² = 6.90, df = 3 (P = 0.08); I² = 57%						
Test for overall effect: Z = 0.61 (P = 0.54)						
Test for subgroup differences: Chi² = 6.89, df = 2 (P = 0.03), I² = 71.0%						

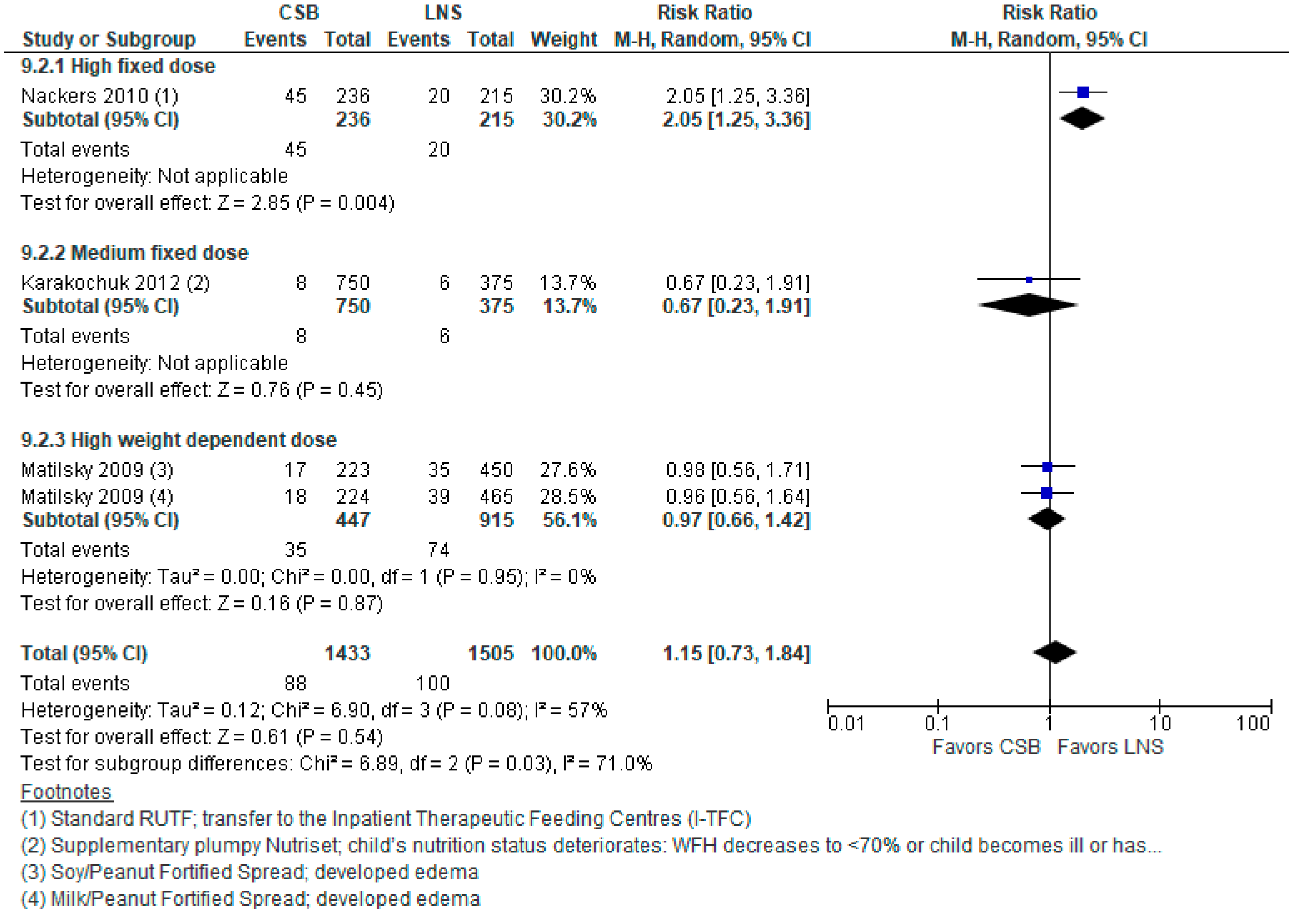

Footnotes
(1) Standard RUTF; transfer to the Inpatient Therapeutic Feeding Centres (I-TFC)
(2) Supplementary plumpy Nutriset; child's nutrition status deteriorates: WFH decreases to <70% or child becomes ill or has...
(3) Soy/Peanut Fortified Spread; developed edema
(4) Milk/Peanut Fortified Spread; developed edema

Supplementary Figure S36. Forest plot of CSB vs LNS - Outcome: Non-response (subgroup analysis by dose)

Study or Subgroup	CSB Events	CSB Total	LNS Events	LNS Total	Weight	Risk Ratio M-H, Random, 95% CI
**9.3.1 High fixed dose**						
Nackers 2010 (1)	13	215	21	236	21.8%	0.68 [0.35, 1.32]
**Subtotal (95% CI)**		**215**		**236**	**21.8%**	**0.68 [0.35, 1.32]**
Total events	13		21			
Heterogeneity: Not applicable						
Test for overall effect: Z = 1.14 (P = 0.26)						
**9.3.2 Medium fixed dose**						
Karakochuk 2012 (2)	86	375	216	750	28.1%	0.80 [0.64, 0.99]
**Subtotal (95% CI)**		**375**		**750**	**28.1%**	**0.80 [0.64, 0.99]**
Total events	86		216			
Heterogeneity: Not applicable						
Test for overall effect: Z = 2.06 (P = 0.04)						
**9.3.3 High weight dependent dose**						
Matilsky 2009 (3)	33	223	29	450	24.9%	2.30 [1.43, 3.68]
Matilsky 2009 (4)	34	224	35	465	25.3%	2.02 [1.29, 3.14]
**Subtotal (95% CI)**		**447**		**915**	**50.2%**	**2.14 [1.55, 2.96]**
Total events	67		64			
Heterogeneity: Tau² = 0.00; Chi² = 0.15, df = 1 (P = 0.69); I² = 0%						
Test for overall effect: Z = 4.62 (P < 0.00001)						
**Total (95% CI)**		**1037**		**1901**	**100.0%**	**1.27 [0.68, 2.36]**
Total events	166		301			
Heterogeneity: Tau² = 0.35; Chi² = 26.73, df = 3 (P < 0.00001); I² = 89%						
Test for overall effect: Z = 0.74 (P = 0.46)						
Test for subgroup differences: Chi² = 26.56, df = 2 (P < 0.00001), I² = 92.5%						

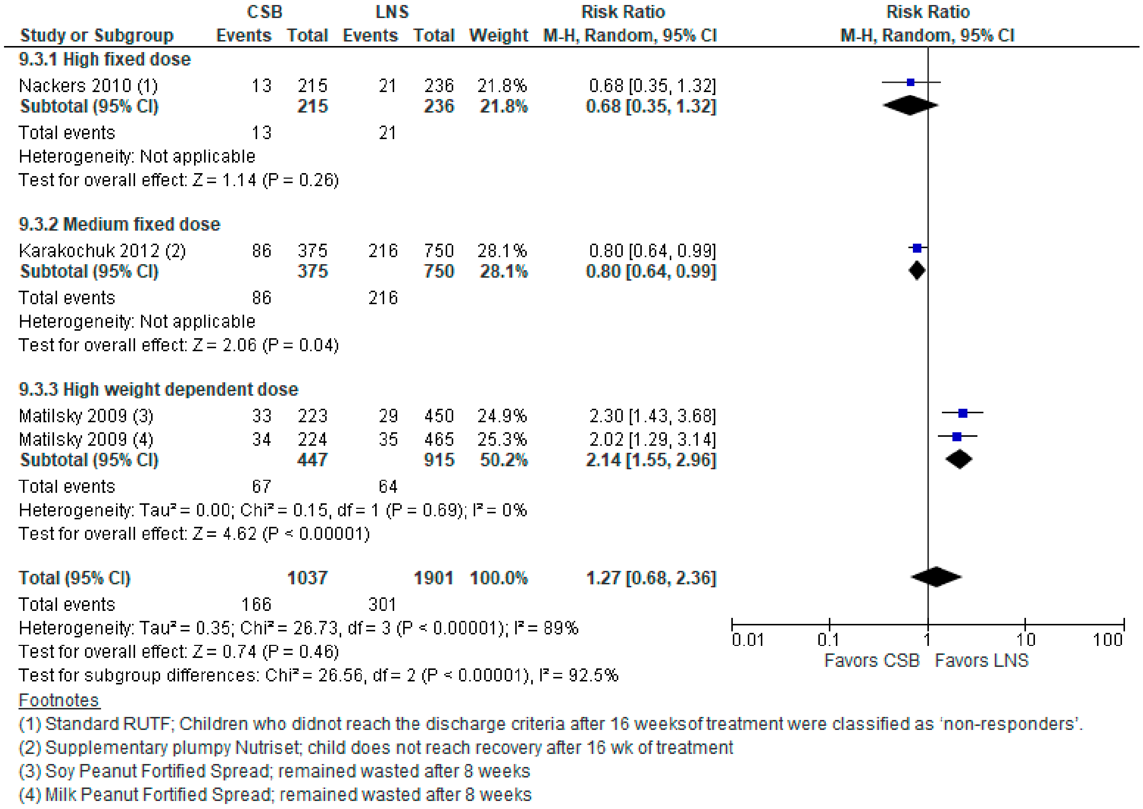

Footnotes
(1) Standard RUTF; Children who did not reach the discharge criteria after 16 weeks of treatment were classified as 'non-responders'.
(2) Supplementary plumpy Nutriset; child does not reach recovery after 16 wk of treatment
(3) Soy Peanut Fortified Spread; remained wasted after 8 weeks
(4) Milk Peanut Fortified Spread; remained wasted after 8 weeks

Supplementary Figure S37. Forest plot of CSB vs LNS - Outcome: Recovery rate (subgroup analysis by duration)

	CSB		LNS			Risk Ratio
Study or Subgroup	Events	Total	Events	Total	Weight	M-H, Random, 95% CI
**10.1.1 By 8 weeks**						
Matilsky 2009 (1)	162	224	369	465	25.9%	0.91 [0.83, 1.00]
Matilsky 2009 (2)	161	223	360	450	25.8%	0.90 [0.82, 0.99]
**Subtotal (95% CI)**		**447**		**915**	**51.7%**	**0.91 [0.85, 0.97]**
Total events	323		729			
Heterogeneity: Tau² = 0.00; Chi² = 0.02, df = 1 (P = 0.88); I² = 0%						
Test for overall effect: Z = 2.90 (P = 0.004)						
**10.1.2 By 16 weeks**						
Karakochuk 2012 (3)	482	750	265	375	31.8%	0.91 [0.84, 0.99]
Nackers 2010 (4)	152	236	170	215	16.5%	0.81 [0.72, 0.92]
**Subtotal (95% CI)**		**986**		**590**	**48.3%**	**0.87 [0.78, 0.97]**
Total events	634		435			
Heterogeneity: Tau² = 0.00; Chi² = 2.24, df = 1 (P = 0.13); I² = 55%						
Test for overall effect: Z = 2.61 (P = 0.009)						
**Total (95% CI)**		**1433**		**1505**	**100.0%**	**0.89 [0.85, 0.94]**
Total events	957		1164			
Heterogeneity: Tau² = 0.00; Chi² = 2.77, df = 3 (P = 0.43); I² = 0%						
Test for overall effect: Z = 4.72 (P < 0.00001)						
Test for subgroup differences: Chi² = 0.48, df = 1 (P = 0.49), I² = 0%						

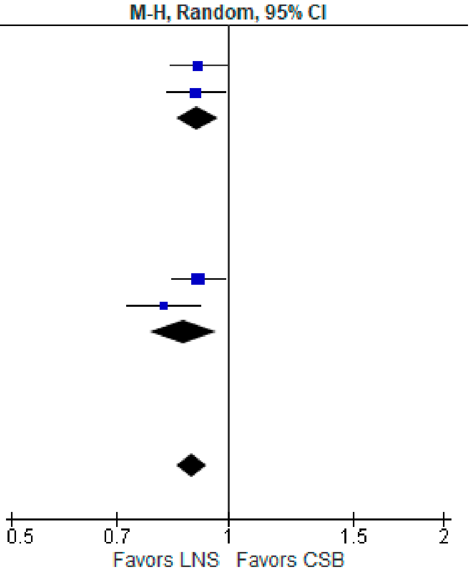

Footnotes
(1) Milk/Peanut Fortified Spread; defined as having a WHZ > -2
(2) Soy/Peanut Fortified Spread; defined as having a WHZ > -2
(3) Supplementary plumpy Nutriset; recovery was defined as WFH ≥ 85% on 2 consecutive visits within 16 week duration
(4) Standard RUTF; WHM% >/= 85% for 2 consecutive weeks

Supplementary Figure S38. Forest plot of CSB vs LNS - Outcome: Deterioration to severe wasting (subgroup analysis by duration)

	CSB		LNS			Risk Ratio
Study or Subgroup	Events	Total	Events	Total	Weight	M-H, Random, 95% CI
**10.2.1 By 8 weeks**						
Matilsky 2009 (1)	18	224	39	465	28.5%	0.96 [0.56, 1.64]
Matilsky 2009 (2)	17	223	35	450	27.6%	0.98 [0.56, 1.71]
**Subtotal (95% CI)**		**447**		**915**	**56.1%**	**0.97 [0.66, 1.42]**
Total events	35		74			
Heterogeneity: Tau² = 0.00; Chi² = 0.00, df = 1 (P = 0.95); I² = 0%						
Test for overall effect: Z = 0.16 (P = 0.87)						
**10.2.2 By 16 weeks**						
Karakochuk 2012 (3)	8	750	6	375	13.7%	0.67 [0.23, 1.91]
Nackers 2010 (4)	45	236	20	215	30.2%	2.05 [1.25, 3.36]
**Subtotal (95% CI)**		**986**		**590**	**43.9%**	**1.29 [0.44, 3.82]**
Total events	53		26			
Heterogeneity: Tau² = 0.46; Chi² = 3.59, df = 1 (P = 0.06); I² = 72%						
Test for overall effect: Z = 0.46 (P = 0.64)						
**Total (95% CI)**		**1433**		**1505**	**100.0%**	**1.15 [0.73, 1.84]**
Total events	88		100			
Heterogeneity: Tau² = 0.12; Chi² = 6.90, df = 3 (P = 0.08); I² = 57%						
Test for overall effect: Z = 0.61 (P = 0.54)						
Test for subgroup differences: Chi² = 0.24, df = 1 (P = 0.62), I² = 0%						

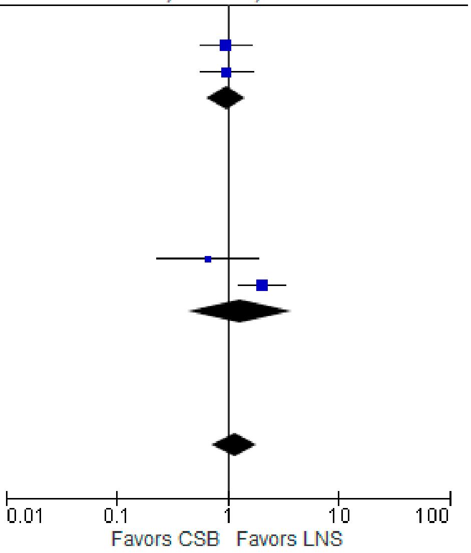

Footnotes
(1) Milk/Peanut Fortified Spread; developed edema
(2) Soy/Peanut Fortified Spread; developed edema
(3) Supplementary plumpy Nutriset; child's nutrition status deteriorates: WFH decreases to <70% or child becomes ill or has...
(4) Standard RUTF; transfer to the Inpatient Therapeutic Feeding Centres (I-TFC)

Supplementary Figure S39. Forest plot of CSB vs LNS - Outcome: Non-response (subgroup analysis by duration)

Study or Subgroup	CSB Events	CSB Total	LNS Events	LNS Total	Weight	Risk Ratio M-H, Random, 95% CI
**10.3.1 By 8 weeks**						
Matilsky 2009 (1)	33	223	29	450	24.9%	2.30 [1.43, 3.68]
Matilsky 2009 (2)	34	224	35	465	25.3%	2.02 [1.29, 3.14]
**Subtotal (95% CI)**		**447**		**915**	**50.2%**	**2.14 [1.55, 2.96]**
Total events	67		64			
Heterogeneity: Tau² = 0.00; Chi² = 0.15, df = 1 (P = 0.69); I² = 0%						
Test for overall effect: Z = 4.62 (P < 0.00001)						
**10.3.2 By 16 weeks**						
Karakochuk 2012 (3)	86	375	216	750	28.1%	0.80 [0.64, 0.99]
Nackers 2010 (4)	13	215	21	236	21.8%	0.68 [0.35, 1.32]
**Subtotal (95% CI)**		**590**		**986**	**49.8%**	**0.78 [0.64, 0.96]**
Total events	99		237			
Heterogeneity: Tau² = 0.00; Chi² = 0.20, df = 1 (P = 0.66); I² = 0%						
Test for overall effect: Z = 2.31 (P = 0.02)						
**Total (95% CI)**		**1037**		**1901**	**100.0%**	**1.27 [0.68, 2.36]**
Total events	166		301			
Heterogeneity: Tau² = 0.35; Chi² = 26.73, df = 3 (P < 0.00001); I² = 89%						
Test for overall effect: Z = 0.74 (P = 0.46)						
Test for subgroup differences: Chi² = 26.36, df = 1 (P < 0.00001), I² = 96.2%						

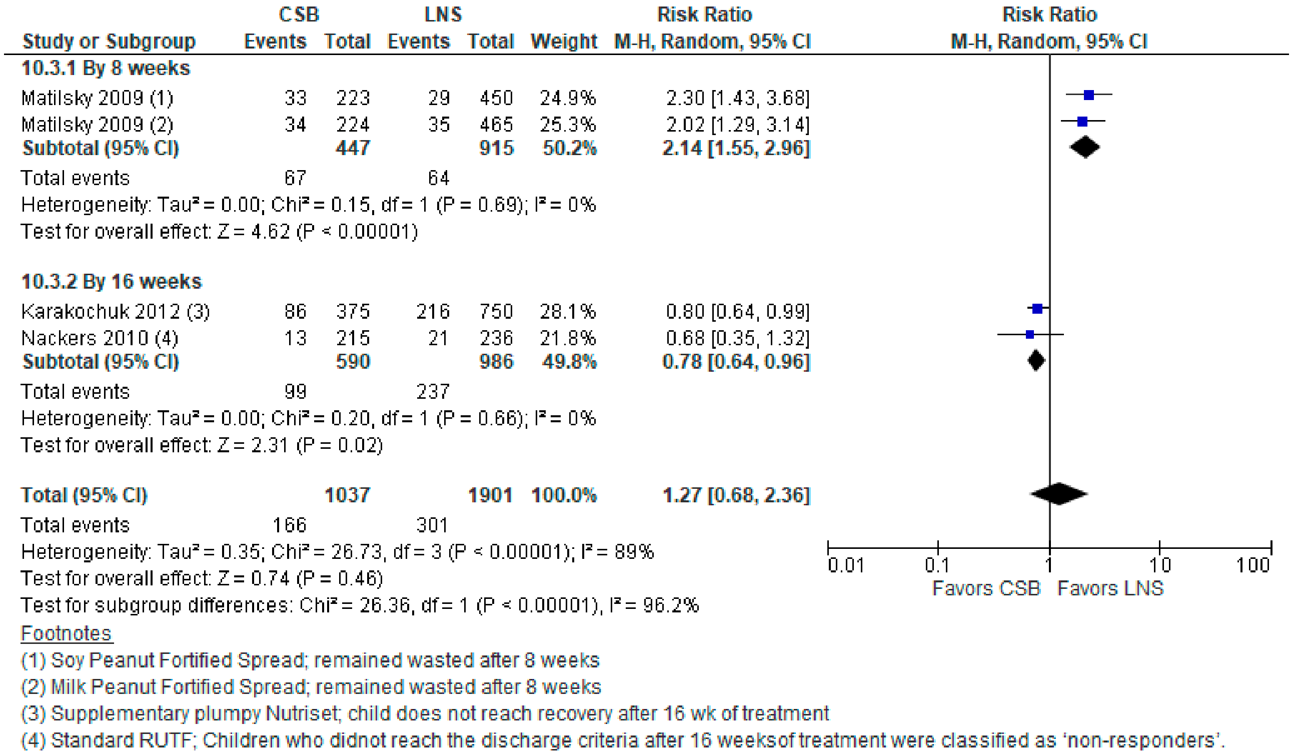

Footnotes
(1) Soy Peanut Fortified Spread; remained wasted after 8 weeks
(2) Milk Peanut Fortified Spread; remained wasted after 8 weeks
(3) Supplementary plumpy Nutriset; child does not reach recovery after 16 wk of treatment
(4) Standard RUTF; Children who didnot reach the discharge criteria after 16 weeksof treatment were classified as 'non-responders'.

Supplementary Figure S40. Forest plot of locally produced FBF compared to LNS - Outcome: Weight for height z-score gain during the 12-week intervention period

Study or Subgroup	Locally produced FBFs Mean	SD	Total	LNS Mean	SD	Total	Weight	Mean Difference IV, Random, 95% CI
Ackatia-Armah 2015 (1)	0.54	1.02	281	0.94	1.05	167	49.2%	-0.40 [-0.60, -0.20]
Ackatia-Armah 2015 (2)	0.65	1.02	306	0.94	1.05	168	50.8%	-0.29 [-0.49, -0.09]
**Total (95% CI)**			**587**			**335**	**100.0%**	**-0.34 [-0.48, -0.20]**
Heterogeneity: Tau² = 0.00; Chi² = 0.60, df = 1 (P = 0.44); I² = 0%								
Test for overall effect: Z = 4.83 (P < 0.00001)								

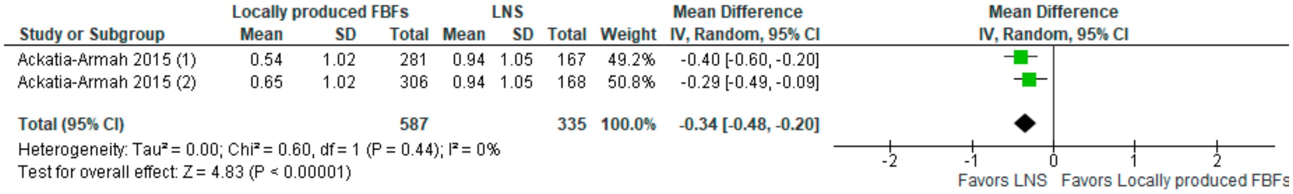

Footnotes
(1) Locally milled flours (LMF) vs RUSF; change in WHZ during 12 weeks of treatment
(2) Misola vs RUSF; change in WHZ during 12 weeks of treatment

Supplementary Figure S41. Forest plot of locally produced FBF compared to LNS - Outcome: Change in MUAC over the 12-week intervention period

Study or Subgroup	Locally produced FBFs Mean	SD	Total	LNS Mean	SD	Total	Weight	Mean Difference IV, Random, 95% CI
Ackatia-Armah 2015 (1)	0.7	0.85	281	1.1	0.91	167	50.0%	-0.40 [-0.57, -0.23]
Ackatia-Armah 2015 (2)	0.8	0.89	306	1.1	0.91	168	50.0%	-0.30 [-0.47, -0.13]
**Total (95% CI)**			**587**			**335**	**100.0%**	**-0.35 [-0.47, -0.23]**
Heterogeneity: Tau² = 0.00; Chi² = 0.66, df = 1 (P = 0.41); I² = 0%								
Test for overall effect: Z = 5.71 (P < 0.00001)								

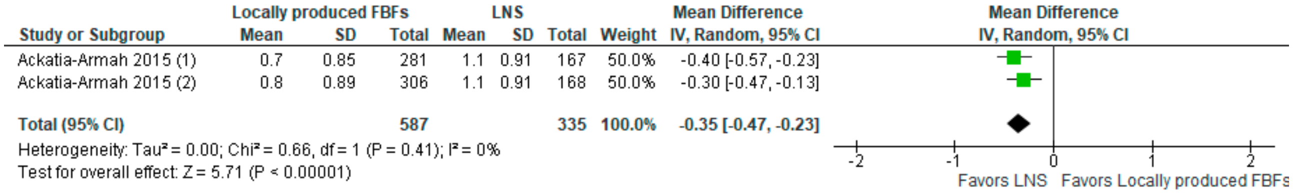

Footnotes
(1) Locally milled flours (LMF) vs RUSF; Change in MUAC in cm over the 12 week intervention period
(2) Misola vs RUSF; Change in MUAC in cm over the 12 week intervention period

Supplementary Figure S42. Forest plot of locally produced FBF compared to LNS - Outcome: Weight gain (kg) over the 12-week intervention period

Study or Subgroup	Locally produced FBFs Mean	SD	Total	LNS Mean	SD	Total	Weight	Mean Difference IV, Random, 95% CI
Ackatia-Armah 2015 (1)	0.91	0.75	306	1.16	0.73	168	51.4%	-0.25 [-0.39, -0.11]
Ackatia-Armah 2015 (2)	0.83	0.77	281	1.16	0.73	167	48.6%	-0.33 [-0.47, -0.19]
**Total (95% CI)**			**587**			**335**	**100.0%**	**-0.29 [-0.39, -0.19]**

Heterogeneity: Tau² = 0.00; Chi² = 0.62, df = 1 (P = 0.43); I² = 0%
Test for overall effect: Z = 5.69 (P < 0.00001)

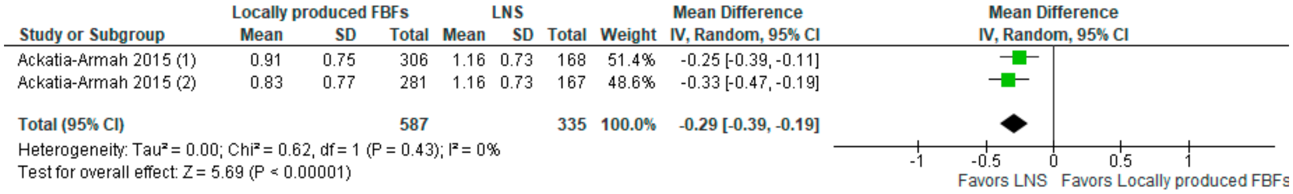

Footnotes
(1) Misola vs RUSF; Weight gain in kg over the 12 week intervention period
(2) LMF vs RUSF; Weight gain in kg over the 12 week intervention period

Supplementary Figure S43. Forest plot of locally produced FBF compared to LNS - Outcome: Height gain (cm) over the 12-week intervention period

Study or Subgroup	Locally produced FBFs Mean	SD	Total	LNS Mean	SD	Total	Weight	Mean Difference IV, Random, 95% CI
Ackatia-Armah 2015 (1)	2.6	1.28	281	2.9	1.37	167	56.0%	-0.30 [-0.56, -0.04]
Ackatia-Armah 2015 (2)	2.7	1.8	306	2.9	1.37	168	44.0%	-0.20 [-0.49, 0.09]
**Total (95% CI)**			**587**			**335**	**100.0%**	**-0.26 [-0.45, -0.06]**

Heterogeneity: Tau² = 0.00; Chi² = 0.26, df = 1 (P = 0.61); I² = 0%
Test for overall effect: Z = 2.62 (P = 0.009)

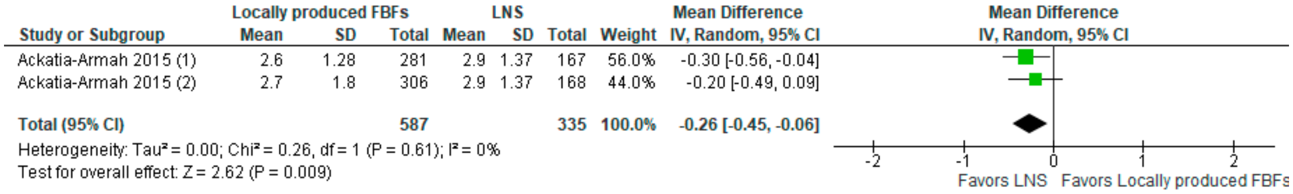

Footnotes
(1) Locally milled flours (LMF) vs RUSF; Height gain in cm over the 12 week intervention period
(2) Misola vs RUSF; Height gain in cm over the 12 week intervention period

Supplementary Figure S44. Forest plot of locally produced FBF compared to LNS - Outcome: Non-response

Study or Subgroup	Locally produced FBFs Events	Total	LNS Events	Total	Weight	Risk Ratio M-H, Random, 95% CI
Ackatia-Armah 2015 (1)	115	281	39	168	51.1%	1.76 [1.29, 2.40]
Ackatia-Armah 2015 (2)	105	306	39	167	48.9%	1.47 [1.07, 2.01]
**Total (95% CI)**		**587**		**335**	**100.0%**	**1.61 [1.29, 2.01]**
Total events	220		78			

Heterogeneity: Tau² = 0.00; Chi² = 0.65, df = 1 (P = 0.42); I² = 0%
Test for overall effect: Z = 4.24 (P < 0.0001)

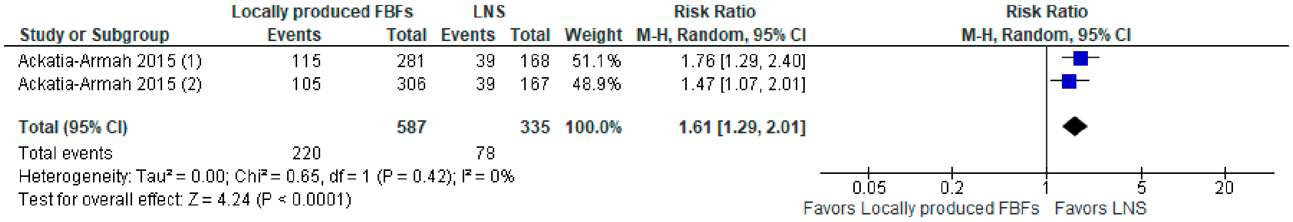

Footnotes
(1) Locally milled flours (LMF) vs RUSF; not recovered by week 12
(2) Misola vs RUSF; not recovered by week 12

Supplementary Figure S45. Forest plot of locally produced FBF compared to LNS - Outcome: Time to recovery (weeks)

Study or Subgroup	Locally produced FBFs Mean	SD	Total	LNS Mean	SD	Total	Weight	Mean Difference IV, Random, 95% CI
Ackatia-Armah 2015 (1)	9.9	0.69	163	5.9	0.37	122	50.0%	4.00 [3.88, 4.12]
Ackatia-Armah 2015 (2)	8.7	0.6	187	5.9	0.37	123	50.0%	2.80 [2.69, 2.91]
**Total (95% CI)**			**350**			**245**	**100.0%**	**3.40 [2.22, 4.58]**

Heterogeneity: Tau² = 0.72; Chi² = 203.36, df = 1 (P < 0.00001); I² = 100%
Test for overall effect: Z = 5.67 (P < 0.00001)

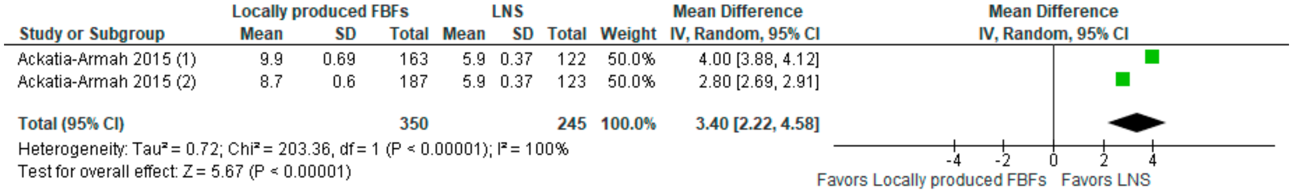

Footnotes
(1) Locally milled flours (LMF) vs RUSF; Time to recovery in weeks
(2) Misola vs RUSF; Time to recovery in weeks
