# Supplementary material for: Effectiveness of Dietary Management for Moderate Wasting among Children > 6 Months of Age—A Systematic Review and Meta-Analysis Exploring Different Types, Quantities, and Durations
Source: Nutrients. 2023 Feb 21;15(5):1076. doi: 10.3390/nu15051076 (PMC10005276; doi:10.3390/nu15051076)
Supplement: Supplementary file 1 [file nutrients-15-01076-s001.zip › Annex 3. Supplementary Figures S46_Subquestion 2.pdf]

Supplementary Figure S46. Forest plot of Enhanced FBF compared to locally produced FBFs - Outcome: Deterioration to severe wasting during the intervention period

Study or Subgroup	Enhanced FBFs Events	Enhanced FBFs Total	Locally produced FBFs Events	Locally produced FBFs Total	Weight	Risk Ratio M-H, Random, 95% CI
Nane 2021 (1)	4	162	2	162	100.0%	2.00 [0.37, 10.77]
**Total (95% CI)**		**162**		**162**	**100.0%**	**2.00 [0.37, 10.77]**
Total events	4		2			
Heterogeneity: Not applicable						
Test for overall effect: Z = 0.81 (P = 0.42)						

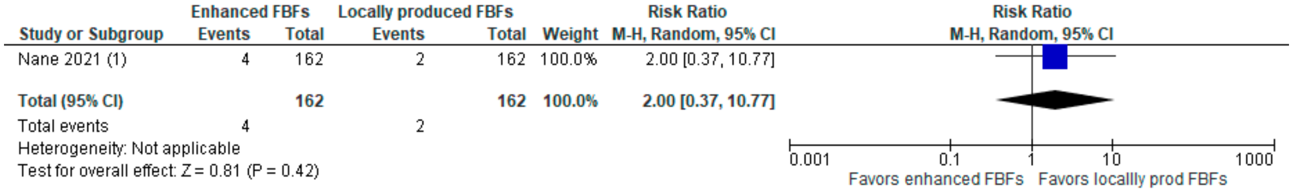

Footnotes
(1) CSB+ vs. local ingredients based supplement (LIBS); Deterioration to severe wasting at any point during the intervention.

Supplementary Figure S47. Forest plot of Enhanced FBF compared to locally produced FBFs - Outcome: WHZ

Study or Subgroup	Enhanced FBFs Mean	Enhanced FBFs SD	Enhanced FBFs Total	Locally produced FBFs Mean	Locally produced FBFs SD	Locally produced FBFs Total	Weight	Mean Difference IV, Random, 95% CI
Ackatia-Armah 2015 (1)	0.76	1.12	171	0.54	1.02	281	24.3%	0.22 [0.01, 0.43]
Ackatia-Armah 2015 (2)	0.76	1.12	171	0.65	1.02	306	24.4%	0.11 [-0.09, 0.31]
Amegovu 2014 (3)	-1.149	0.43	88	-0.88	0.339	90	27.5%	-0.27 [-0.38, -0.16]
Kajjura 2019 (4)	-1.276	0.846	100	-1.068	0.755	104	23.7%	-0.21 [-0.43, 0.01]
**Total (95% CI)**			**530**			**781**	**100.0%**	**-0.04 [-0.29, 0.20]**
Heterogeneity: Tau² = 0.05; Chi² = 22.58, df = 3 (P < 0.0001); I² = 87%								
Test for overall effect: Z = 0.34 (P = 0.73)								

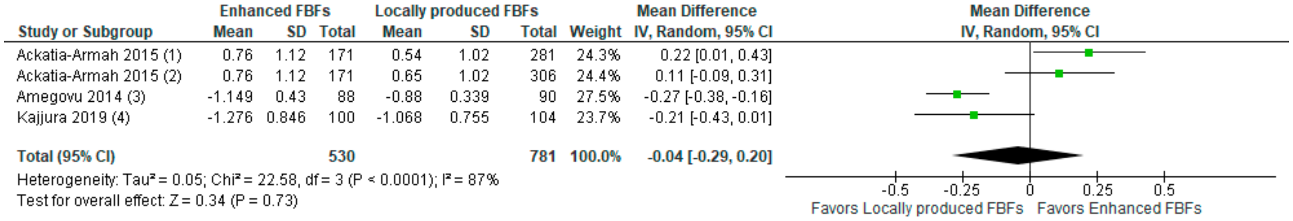

Footnotes
(1) CSB++ vs. locally milled flours (LMF); Change in WHZ during 12 weeks of treatment.
(2) CSB++ vs. Misola; Change in WHZ during 12 weeks of treatment.
(3) CSB+ vs. locally produced soy peanut blend (SPB); WHZ at discharge.
(4) CSB+ vs. Malted sorghum-based porridge (MSBP); WHZ at endline (after 3 months).

Supplementary Figure S48. Forest plot of Enhanced FBF compared to locally produced FBFs - Outcome: WAZ

Study or Subgroup	Enhanced FBFs Mean	Enhanced FBFs SD	Enhanced FBFs Total	Locally produced FBFs Mean	Locally produced FBFs SD	Locally produced FBFs Total	Weight	Mean Difference IV, Random, 95% CI
Kajjura 2019 (1)	-2.214	0.817	100	-1.939	0.69	104	100.0%	-0.27 [-0.48, -0.07]
**Total (95% CI)**			**100**			**104**	**100.0%**	**-0.27 [-0.48, -0.07]**
Heterogeneity: Not applicable								
Test for overall effect: Z = 2.59 (P = 0.010)								

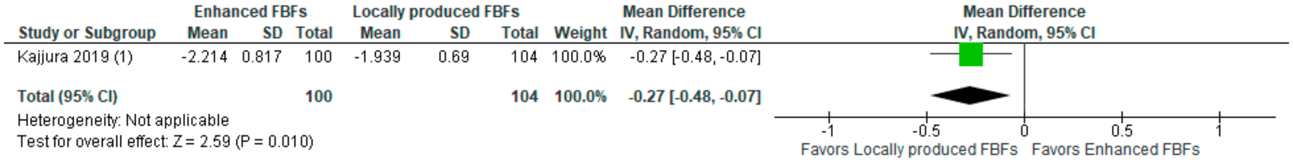

Footnotes
(1) CSB+ vs. Malted sorghum-based porridge (MSBP); WAZ measured at endline (after 3 months).

Supplementary Figure S49. Forest plot of Enhanced FBF compared to locally produced FBFs - Outcome: HAZ

Study or Subgroup	Enhanced FBFs Mean	Enhanced FBFs SD	Enhanced FBFs Total	Locally produced FBFs Mean	Locally produced FBFs SD	Locally produced FBFs Total	Weight	Mean Difference IV, Random, 95% CI
Amegovu 2014 (1)	-1.6	1.48	88	-1.6	1.45	90	30.9%	0.00 [-0.43, 0.43]
Kajjura 2019 (2)	-2.521	1.057	100	-2.282	1.038	104	69.1%	-0.24 [-0.53, 0.05]
**Total (95% CI)**			**188**			**194**	**100.0%**	**-0.17 [-0.40, 0.07]**
Heterogeneity: Tau² = 0.00; Chi² = 0.82, df = 1 (P = 0.37); I² = 0%								
Test for overall effect: Z = 1.35 (P = 0.18)								

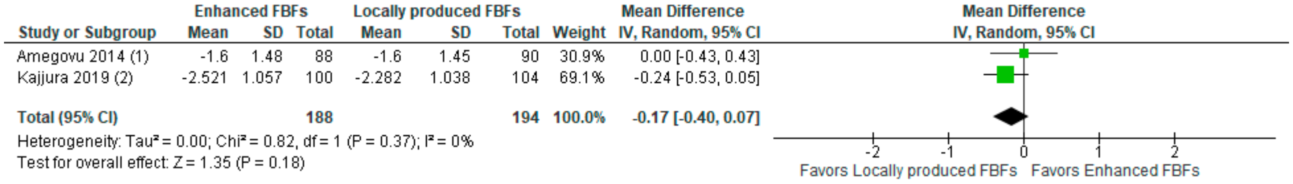

Footnotes
(1) CSB+ vs. locally produced soy peanut blend (SPB); HAZ measured at discharge.
(2) CSB+ vs. Malted sorghum-based porridge (MSBP); HAZ measured at endline (after 3 months).

Supplementary Figure S50. Forest plot of Enhanced FBF compared to locally produced FBFs - Outcome: MUAC gain (in cm)

	Enhanced FBFs			Locally produced FBFs				Mean Difference
**Study or Subgroup**	**Mean**	**SD**	**Total**	**Mean**	**SD**	**Total**	**Weight**	**IV, Random, 95% CI**
Ackatia-Armah 2015 (1)	0.9	0.94	171	0.8	0.89	306	32.2%	0.10 [-0.07, 0.27]
Ackatia-Armah 2015 (2)	0.9	0.94	171	0.7	0.85	281	32.2%	0.20 [0.03, 0.37]
Nane 2021 (3)	0.9	0.67	162	1.01	0.59	162	35.7%	-0.11 [-0.25, 0.03]
**Total (95% CI)**			**504**			**749**	**100.0%**	**0.06 [-0.13, 0.25]**

Heterogeneity: Tau² = 0.02; Chi² = 8.34, df = 2 (P = 0.02); I² = 76%
Test for overall effect: Z = 0.59 (P = 0.55)

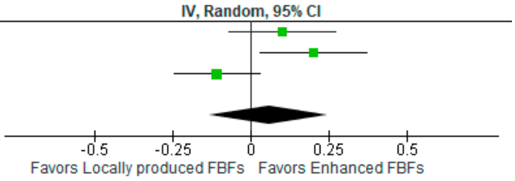

Footnotes
(1) CSB++ vs. misola; MUAC in cm.
(2) CSB++ vs. locally milled flours (LMF); MUAC in cm.
(3) CSB+ vs. local ingredients based supplement (LIBS); MUAC in cm.

Supplementary Figure S51. Forest plot of Enhanced FBF compared to locally produced FBFs - Outcome: Weight gain

	Enhanced FBFs			Locally produced FBFs				Mean Difference
**Study or Subgroup**	**Mean**	**SD**	**Total**	**Mean**	**SD**	**Total**	**Weight**	**IV, Random, 95% CI**
Ackatia-Armah 2015 (1)	1.04	0.66	171	0.83	0.77	281	28.6%	0.21 [0.08, 0.34]
Ackatia-Armah 2015 (2)	1.04	0.66	171	0.91	0.75	306	30.0%	0.13 [0.00, 0.26]
Kajjura 2019 (3)	1.086	0.454	100	0.988	0.417	104	33.8%	0.10 [-0.02, 0.22]
Nane 2021 (4)	2.06	1.36	162	2.14	1.27	162	7.6%	-0.08 [-0.37, 0.21]
**Total (95% CI)**			**604**			**853**	**100.0%**	**0.13 [0.05, 0.21]**

Heterogeneity: Tau² = 0.00; Chi² = 3.71, df = 3 (P = 0.29); I² = 19%
Test for overall effect: Z = 3.05 (P = 0.002)

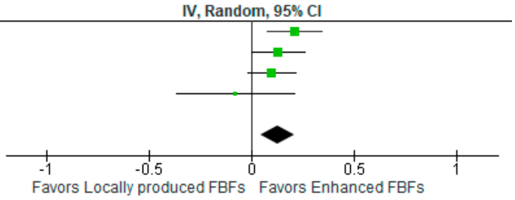

Footnotes
(1) CSB++ vs. locally milled flours (LMF); Weight gain in kg over the intervention period.
(2) CSB++ vs. misola; Weight gain in kg over the intervention period.
(3) CSB+ vs. malted sorghum-based porridge (MSBP); Weight gain in kg over the intervention period.
(4) CSB+ vs. local ingredients based supplement (LIBS); Weight gain in kg over the intervention period.

Supplementary Figure S52. Forest plot of Enhanced FBF compared to locally produced FBFs - Outcome: Height gain

	Enhanced FBFs			Locally produced FBFs				Mean Difference
**Study or Subgroup**	**Mean**	**SD**	**Total**	**Mean**	**SD**	**Total**	**Weight**	**IV, Random, 95% CI**
Ackatia-Armah 2015 (1)	2.9	1.41	171	2.7	1.8	306	23.5%	0.20 [-0.09, 0.49]
Ackatia-Armah 2015 (2)	2.9	1.41	171	2.6	1.28	281	25.4%	0.30 [0.04, 0.56]
Kajjura 2019 (3)	2.9	0.1	100	2.99	0.11	104	35.9%	-0.09 [-0.12, -0.06]
Nane 2021 (4)	3.02	2.1	162	3.28	2.18	162	15.3%	-0.26 [-0.73, 0.21]
**Total (95% CI)**			**604**			**853**	**100.0%**	**0.05 [-0.19, 0.29]**

Heterogeneity: Tau² = 0.04; Chi² = 12.79, df = 3 (P = 0.005); I² = 77%
Test for overall effect: Z = 0.42 (P = 0.68)

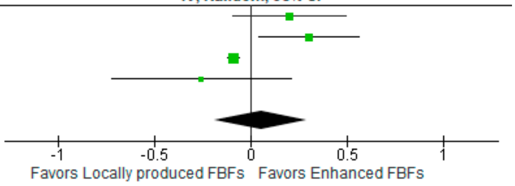

Footnotes
(1) CSB++ vs. misola; Height gain in cm over the intervention period.
(2) CSB++ vs. locally milled flours (LMF); Height gain in cm over the intervention period.
(3) CSB+ vs. Malted sorghum-based porridge (MSBP); mean increase in length in cm
(4) CSB+ vs. local ingredients based supplement (LIBS); increase in height in cm over the intervention period.

Supplementary Figure S53. Forest plot of Enhanced FBF compared to locally produced FBFs - Outcome: Non-response

Study or Subgroup	Enhanced FBFs Events	Enhanced FBFs Total	Locally produced FBFs Events	Locally produced FBFs Total	Weight	Risk Ratio M-H, Random, 95% CI
Ackatia-Armah 2015 (1)	58	171	105	306	46.9%	0.99 [0.76, 1.28]
Ackatia-Armah 2015 (2)	57	171	115	281	48.9%	0.81 [0.63, 1.05]
Amegovu 2014 (3)	10	88	7	90	4.2%	1.46 [0.58, 3.67]
**Total (95% CI)**		**430**		**677**	**100.0%**	**0.91 [0.76, 1.10]**
Total events	125		227			
Heterogeneity: Tau² = 0.00; Chi² = 2.14, df = 2 (P = 0.34); I² = 7%						
Test for overall effect: Z = 0.93 (P = 0.35)						

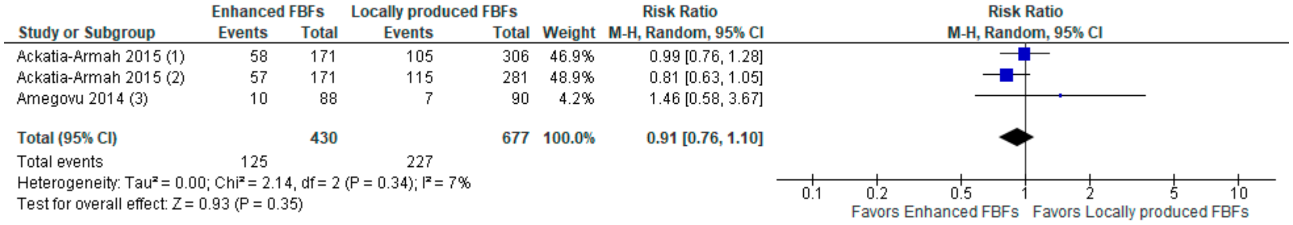

Footnotes
(1) CSB++ vs. Misola; Still MAM after 12 weeks of treatment.
(2) CSB++ vs. locally milled flours (LMF); Still MAM after 12 weeks of treatment.
(3) CSB+ vs. locally produced soy peanut blend (SPB). Still MAM after 12 weeks of treatment.

Supplementary Figure S54. Forest plot of Enhanced FBF compared to locally produced FBFs - Outcome: Time to recovery

Study or Subgroup	Enhanced FBFs Mean	Enhanced FBFs SD	Enhanced FBFs Total	Locally produced FBFs Mean	Locally produced FBFs SD	Locally produced FBFs Total	Weight	Mean Difference IV, Random, 95% CI
Ackatia-Armah 2015 (1)	49	17.2	104	60.9	17.5	187	33.8%	-11.90 [-16.05, -7.75]
Ackatia-Armah 2015 (2)	49	17.2	103	69.3	19.6	163	33.6%	-20.30 [-24.78, -15.82]
Nane 2021 (3)	56.9	25.99	162	54.27	26.74	162	32.6%	2.63 [-3.11, 8.37]
**Total (95% CI)**			**369**			**512**	**100.0%**	**-9.98 [-21.93, 1.96]**
Heterogeneity: Tau² = 105.30; Chi² = 38.08, df = 2 (P < 0.00001); I² = 95%								
Test for overall effect: Z = 1.64 (P = 0.10)								

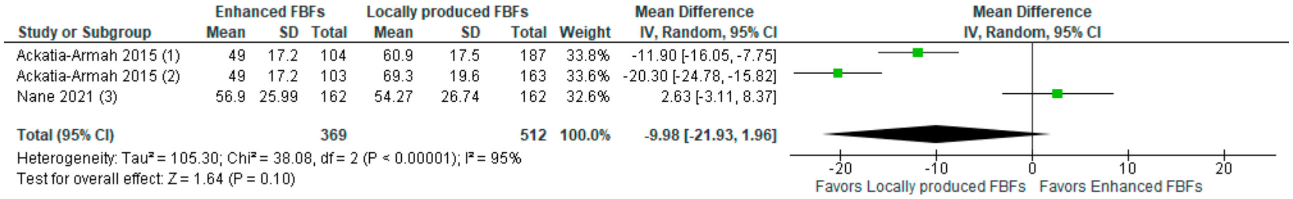

Footnotes
(1) CSB++ vs. Misola; Time to recovery in days.
(2) CSB++ vs. LMF; Time to recovery in days.
(3) CSB+ vs. local ingredients based supplement (LIBS); Time to recovery in days.

Supplementary Figure S55. Forest plot of Enhanced FBFs without milk compared to enhanced FBFs with milk - Outcome: Recovery

Study or Subgroup	Experimental Events	Experimental Total	Control Events	Control Total	Weight	Risk Ratio M-H, Random, 95% CI
**1.1.1 Recovery**						
Griswold 2021 (1)	212	332	360	579	48.5%	1.03 [0.93, 1.14]
Griswold 2021 (2)	212	331	415	663	51.5%	1.02 [0.93, 1.13]
**Subtotal (95% CI)**		**663**		**1242**	**100.0%**	**1.03 [0.95, 1.10]**
Total events	424		775			
Heterogeneity: Tau² = 0.00; Chi² = 0.00, df = 1 (P = 0.96); I² = 0%						
Test for overall effect: Z = 0.68 (P = 0.50)						
**1.1.2 Detrioration to severe wasting**						
Griswold 2021 (3)	60	332	99	579	48.5%	1.06 [0.79, 1.41]
Griswold 2021 (4)	60	331	116	663	51.5%	1.04 [0.78, 1.37]
**Subtotal (95% CI)**		**663**		**1242**	**100.0%**	**1.05 [0.85, 1.28]**
Total events	120		215			
Heterogeneity: Tau² = 0.00; Chi² = 0.01, df = 1 (P = 0.92); I² = 0%						
Test for overall effect: Z = 0.44 (P = 0.66)						

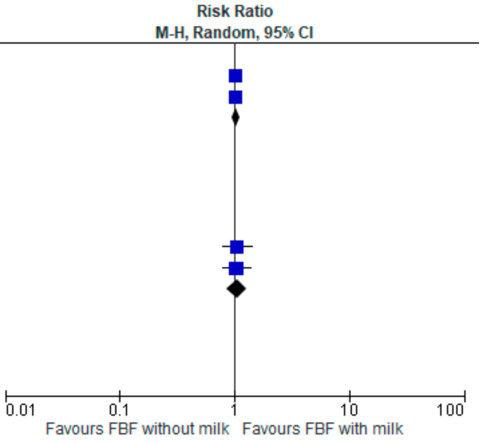

Test for subgroup differences: Chi² = 0.03, df = 1 (P = 0.85), I² = 0%
Footnotes
(1) CSB+ with oil vs CSWB
(2) CSB+ with oil vs Supercereal+ with Amylase
(3) CSB+ with oil vs CSWB
(4) CSB+ with oil vs Supercereal+ with Amylase

Supplementary Figure S56. Comparison of RUTF vs RUSF - Outcome: Recovery

Study or Subgroup	RUTF Events	RUTF Total	RUSF Events	RUSF Total	Weight	Risk Ratio M-H, Random, 95% CI
Bailey 2021 (1)	860	995	773	908	100.0%	1.02 [0.98, 1.05]
**Total (95% CI)**		**995**		**908**	**100.0%**	**1.02 [0.98, 1.05]**
Total events	860		773			
Heterogeneity: Not applicable						
Test for overall effect: Z = 0.81 (P = 0.42)						

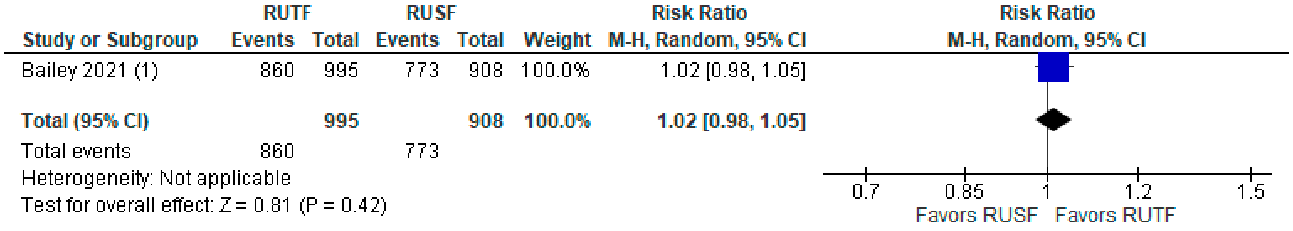

Footnotes
(1) Standard RUTF and RUSF; MUAC ≥125mm and no oedema

Supplementary Figure S57. Comparison of RUTF vs RUSF - Outcome: MUAC gain

Study or Subgroup	RUTF Mean	RUTF SD	RUTF Total	RUSF Mean	RUSF SD	RUSF Total	Weight	Mean Difference IV, Random, 95% CI
Bailey 2021 (1)	12.9	0.4	268	13	0.5	375	100.0%	-0.10 [-0.17, -0.03]
**Total (95% CI)**			**268**			**375**	**100.0%**	**-0.10 [-0.17, -0.03]**
Heterogeneity: Not applicable								
Test for overall effect: Z = 2.81 (P = 0.005)								

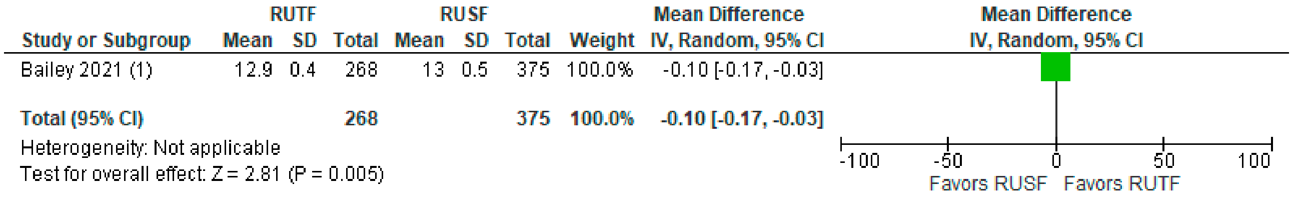

Footnotes
(1) Standard RUTF and RUSF; MUAC in cm at discharge. Data from Kenya subsample only.

Supplementary Figure S58. Comparison of RUTF vs RUSF - Outcome: Weight gain

Study or Subgroup	RUTF Mean	RUTF SD	RUTF Total	RUSF Mean	RUSF SD	RUSF Total	Weight	Mean Difference IV, Random, 95% CI
Bailey 2021 (1)	2	1.2	268	1.73	1.3	375	100.0%	0.27 [0.08, 0.46]
**Total (95% CI)**			**268**			**375**	**100.0%**	**0.27 [0.08, 0.46]**
Heterogeneity: Not applicable								
Test for overall effect: Z = 2.72 (P = 0.007)								

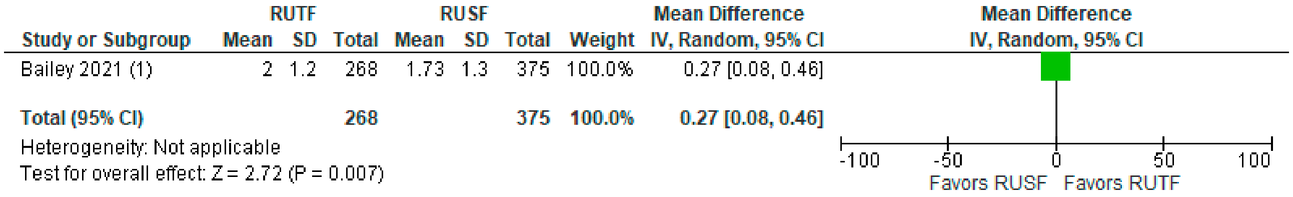

Footnotes
(1) Standard RUTF and RUSF; Weight gain in g/kg/day. Data from Kenya subsample only.

Supplementary Figure S59. Comparison of RUTF vs RUSF - Outcome: Relapse

Study or Subgroup	RUTF Events	RUTF Total	RUSF Events	RUSF Total	Weight	Risk Ratio M-H, Random, 95% CI
Bailey 2021 (1)	21	218	39	318	100.0%	0.79 [0.48, 1.30]
**Total (95% CI)**		**218**		**318**	**100.0%**	**0.79 [0.48, 1.30]**
Total events	21		39			
Heterogeneity: Not applicable						
Test for overall effect: Z = 0.94 (P = 0.35)						

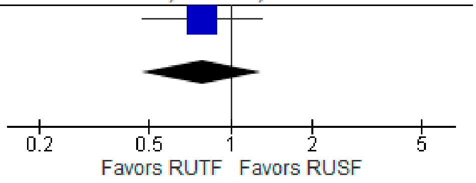

Footnotes
(1) Standard RUTF and RUSF; relapse was defined as MAM cases that relapsed back to GAM. Data from Kenya subsample.

Supplementary Figure S60. Forest plot of RUSF without animal protein vs RUSF with animal protein - Outcome: Recovery rate

Study or Subgroup	RUSF without milk Events	RUSF without milk Total	RUSF with milk Events	RUSF with milk Total	Weight	Risk Ratio M-H, Random, 95% CI
**9.1.3 Soy Peanut RUSF vs. Soy/Whey RUSF**						
LaGrone 2012 (1)	795	906	807	918	44.4%	1.00 [0.96, 1.03]
**Subtotal (95% CI)**		**906**		**918**	**44.4%**	**1.00 [0.96, 1.03]**
Total events	795		807			
Heterogeneity: Not applicable						
Test for overall effect: Z = 0.10 (P = 0.92)						
**9.1.4 Soy RUSF vs. Whey RUSF**						
Stobaugh 2016 (2)	874	1086	960	1144	38.1%	0.96 [0.92, 1.00]
**Subtotal (95% CI)**		**1086**		**1144**	**38.1%**	**0.96 [0.92, 1.00]**
Total events	874		960			
Heterogeneity: Not applicable						
Test for overall effect: Z = 2.12 (P = 0.03)						
**9.1.6 Soy/Peanut vs. Milk/Peanut**						
Matilsky 2009 (3)	360	450	369	465	17.5%	1.01 [0.94, 1.08]
**Subtotal (95% CI)**		**450**		**465**	**17.5%**	**1.01 [0.94, 1.08]**
Total events	360		369			
Heterogeneity: Not applicable						
Test for overall effect: Z = 0.24 (P = 0.81)						
**Total (95% CI)**		**2442**		**2527**	**100.0%**	**0.98 [0.96, 1.01]**
Total events	2029		2136			
Heterogeneity: Tau² = 0.00; Chi² = 2.91, df = 2 (P = 0.23); I² = 31%						
Test for overall effect: Z = 1.00 (P = 0.32)						
Test for subgroup differences: Chi² = 2.89, df = 2 (P = 0.24), I² = 30.7%						

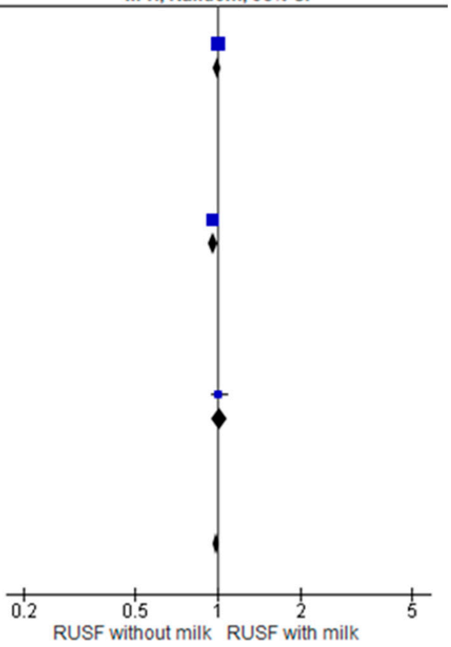

Footnotes
(1) Recovered when they reached a WHZ >= -2
(2) Recovery from MAM, defined as achieving an MUAC of 12.5 cm without bi pedal edema within 12 wk of therapy
(3) Defined as having a WHZ > -2

Supplementary Figure S61. Forest plot of RUSF without animal protein vs RUSF with animal protein - Outcome: Deterioration to severe wasting

Study or Subgroup	Lipid based products Events	Lipid based products Total	Lipid based products Events	Lipid based products Total	Weight	Risk Ratio M-H, Random, 95% CI
**9.5.1 Soy Peanut RUSF vs. Soy/Whey RUSF**						
LaGrone 2012 (1)	75	647	52	667	31.2%	1.49 [1.06, 2.08]
**Subtotal (95% CI)**		**647**		**667**	**31.2%**	**1.49 [1.06, 2.08]**
Total events	75		52			
Heterogeneity: Not applicable						
Test for overall effect: Z = 2.31 (P = 0.02)						
**9.5.2 Soy RUSF vs. Whey RUSF**						
Stobaugh 2016 (2)	128	1086	117	1144	47.5%	1.15 [0.91, 1.46]
**Subtotal (95% CI)**		**1086**		**1144**	**47.5%**	**1.15 [0.91, 1.46]**
Total events	128		117			
Heterogeneity: Not applicable						
Test for overall effect: Z = 1.18 (P = 0.24)						
**9.5.4 Soy/Peanut vs. Milk/Peanut**						
Matilsky 2009 (3)	35	450	39	465	21.3%	0.93 [0.60, 1.44]
**Subtotal (95% CI)**		**450**		**465**	**21.3%**	**0.93 [0.60, 1.44]**
Total events	35		39			
Heterogeneity: Not applicable						
Test for overall effect: Z = 0.34 (P = 0.74)						
**Total (95% CI)**		**2183**		**2276**	**100.0%**	**1.19 [0.95, 1.50]**
Total events	238		208			

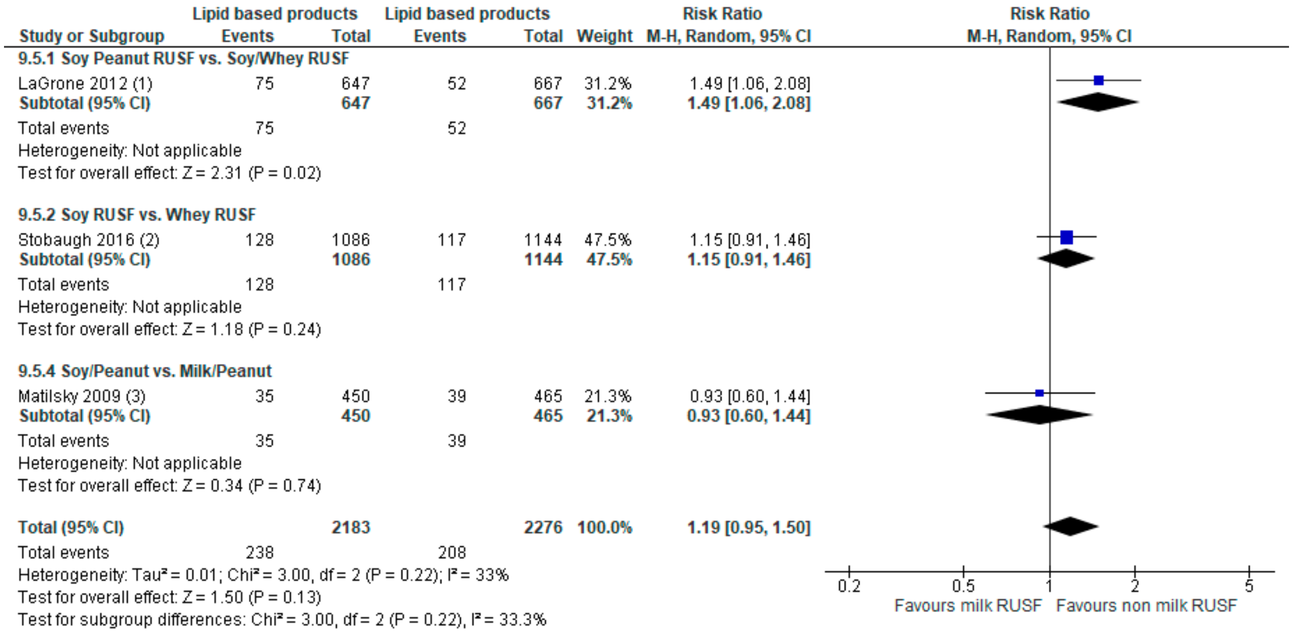

Heterogeneity: Tau² = 0.01; Chi² = 3.00, df = 2 (P = 0.22); I² = 33%
Test for overall effect: Z = 1.50 (P = 0.13)
Test for subgroup differences: Chi² = 3.00, df = 2 (P = 0.22), I² = 33.3%

Footnotes
(1) Developed SAM during the year
(2) Developed SAM
(3) Developed edema

Supplementary Figure S62. Forest plot of RUSF without animal protein vs RUSF with animal protein - Outcome: WHZ score

Study or Subgroup	RUSF without milk Mean	RUSF without milk SD	RUSF without milk Total	RUSF with milk Mean	RUSF with milk SD	RUSF with milk Total	Weight	Mean Difference IV, Random, 95% CI
**9.10.1 MDCF-2 vs RUSF**								
Chen 2021	0.021	0.029	59	0.01	0.027	59	33.5%	0.01 [0.00, 0.02]
**Subtotal (95% CI)**			**59**			**59**	**33.5%**	**0.01 [0.00, 0.02]**
Heterogeneity: Not applicable								
Test for overall effect: Z = 2.13 (P = 0.03)								
**9.10.2 Soy Peanut RUSF vs. Soy/Whey RUSF**								
LaGrone 2012 (1)	-1.61	0.63	906	-1.59	0.6	918	25.7%	-0.02 [-0.08, 0.04]
**Subtotal (95% CI)**			**906**			**918**	**25.7%**	**-0.02 [-0.08, 0.04]**
Heterogeneity: Not applicable								
Test for overall effect: Z = 0.69 (P = 0.49)								
**9.10.3 Soy RUSF vs. Whey RUSF**								
Stobaugh 2016 (2)	-1.18	0.9	1086	-1.08	0.86	1144	22.1%	-0.10 [-0.17, -0.03]
**Subtotal (95% CI)**			**1086**			**1144**	**22.1%**	**-0.10 [-0.17, -0.03]**
Heterogeneity: Not applicable								
Test for overall effect: Z = 2.68 (P = 0.007)								
**9.10.4 Soy/Peanut vs. Milk/Peanut**								
Matilsky 2009 (3)	-1.7	0.7	450	-1.6	0.7	465	18.6%	-0.10 [-0.19, -0.01]
**Subtotal (95% CI)**			**450**			**465**	**18.6%**	**-0.10 [-0.19, -0.01]**
Heterogeneity: Not applicable								
Test for overall effect: Z = 2.16 (P = 0.03)								
**Total (95% CI)**			**2501**			**2586**	**100.0%**	**-0.04 [-0.10, 0.02]**

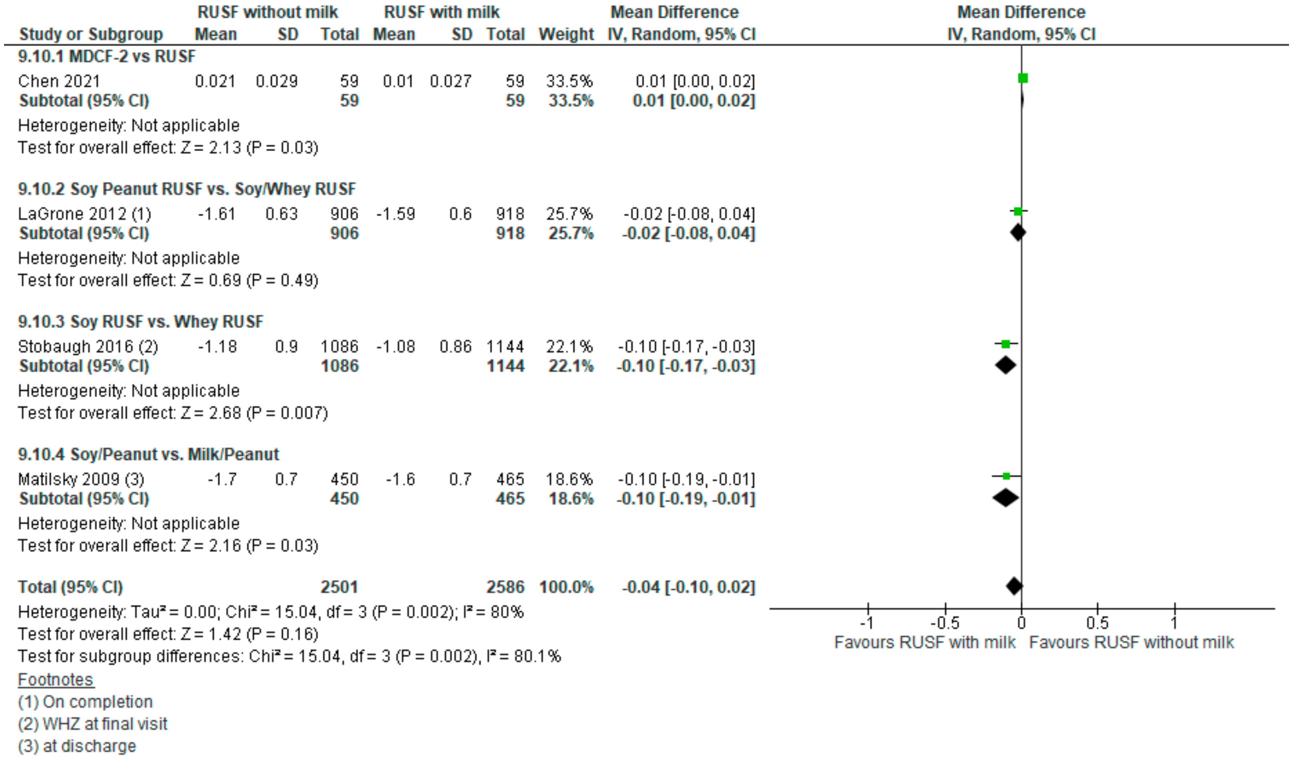

Heterogeneity: Tau² = 0.00; Chi² = 15.04, df = 3 (P = 0.002); I² = 80%
Test for overall effect: Z = 1.42 (P = 0.16)
Test for subgroup differences: Chi² = 15.04, df = 3 (P = 0.002), I² = 80.1%

Footnotes
(1) On completion
(2) WHZ at final visit
(3) at discharge

Supplementary Figure S63. Forest plot of RUSF without animal protein vs RUSF with animal protein - Outcome: WAZ

Study or Subgroup	RUSF without milk Mean	SD	Total	RUSF with milk Mean	SD	Total	Weight	Mean Difference IV, Random, 95% CI
**9.11.2 MDCF-2 vs RUSF**								
Chen 2021	0.017	0.019	59	0.01	0.022	59	100.0%	0.01 [-0.00, 0.01]
**Subtotal (95% CI)**			**59**			**59**	**100.0%**	**0.01 [-0.00, 0.01]**
Heterogeneity: Not applicable								
Test for overall effect: Z = 1.85 (P = 0.06)								

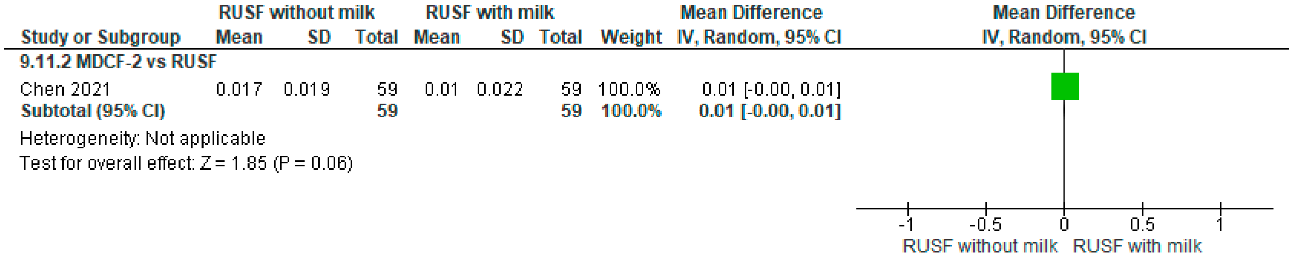

Supplementary Figure S64. Forest plot of RUSF without animal protein vs RUSF with animal protein - Outcome: HAZ

Study or Subgroup	Lipid based products Mean	SD	Total	Lipid based products Mean	SD	Total	Weight	Mean Difference IV, Random, 95% CI
**9.12.1 MDCF-2 vs RUSF**								
Chen 2021 (1)	0.004	0.009	59	0.005	0.009	59	92.3%	-0.00 [-0.00, 0.00]
**Subtotal (95% CI)**			**59**			**59**	**92.3%**	**-0.00 [-0.00, 0.00]**
Heterogeneity: Not applicable								
Test for overall effect: Z = 0.60 (P = 0.55)								
**9.12.2 Soy/Peanut vs. Milk/Peanut**								
Matilsky 2009 (2)	-2.6	1.5	450	-2.7	1.3	465	7.7%	0.10 [-0.08, 0.28]
**Subtotal (95% CI)**			**450**			**465**	**7.7%**	**0.10 [-0.08, 0.28]**
Heterogeneity: Not applicable								
Test for overall effect: Z = 1.08 (P = 0.28)								
**Total (95% CI)**			**509**			**524**	**100.0%**	**0.01 [-0.05, 0.06]**
Heterogeneity: Tau² = 0.00; Chi² = 1.18, df = 1 (P = 0.28); I² = 15%								
Test for overall effect: Z = 0.25 (P = 0.80)								
Test for subgroup differences: Chi² = 1.18, df = 1 (P = 0.28), I² = 15.3%								

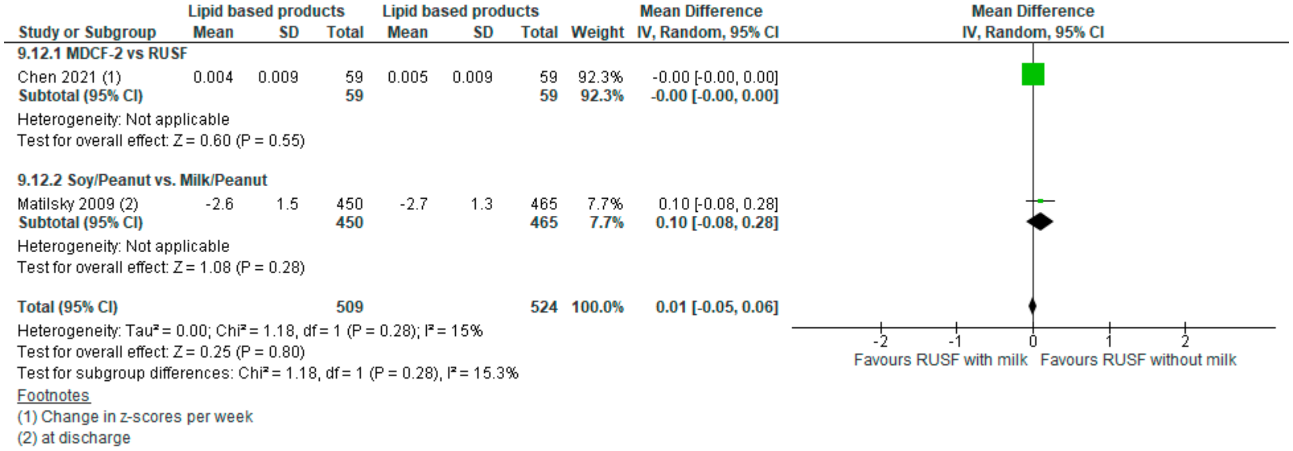

Footnotes
(1) Change in z-scores per week
(2) at discharge

Supplementary Figure S65. Forest plot of RUSF without animal protein vs RUSF with animal protein - Outcome: MUAC gain

Study or Subgroup	RUSF without milk Mean	SD	Total	RUSF with milk Mean	SD	Total	Weight	Mean Difference IV, Random, 95% CI
**9.9.1 MDCF-2 vs RUSF (cm/week)**								
Chen 2021	0.031	0.01	59	0.029	0.013	59	37.2%	0.00 [-0.00, 0.01]
**Subtotal (95% CI)**			**59**			**59**	**37.2%**	**0.00 [-0.00, 0.01]**
Heterogeneity: Not applicable								
Test for overall effect: Z = 0.94 (P = 0.35)								
**9.9.3 Soy Peanut RUSF vs. Soy/Whey RUSF (mm/day)**								
LaGrone 2012 (1)	0.13	0.43	906	0.21	0.44	918	28.7%	-0.08 [-0.12, -0.04]
**Subtotal (95% CI)**			**906**			**918**	**28.7%**	**-0.08 [-0.12, -0.04]**
Heterogeneity: Not applicable								
Test for overall effect: Z = 3.93 (P < 0.0001)								
**9.9.4 Soy RUSF vs. Whey RUSF (mm/day)**								
Stobaugh 2016 (2)	0.22	0.28	1086	0.26	0.27	1144	34.0%	-0.04 [-0.06, -0.02]
**Subtotal (95% CI)**			**1086**			**1144**	**34.0%**	**-0.04 [-0.06, -0.02]**
Heterogeneity: Not applicable								
Test for overall effect: Z = 3.43 (P = 0.0006)								
**Total (95% CI)**			**2051**			**2121**	**100.0%**	**-0.04 [-0.08, 0.01]**
Heterogeneity: Tau² = 0.00; Chi² = 28.06, df = 2 (P < 0.00001); I² = 93%								
Test for overall effect: Z = 1.58 (P = 0.11)								
Test for subgroup differences: Chi² = 28.06, df = 2 (P < 0.00001), I² = 92.9%								

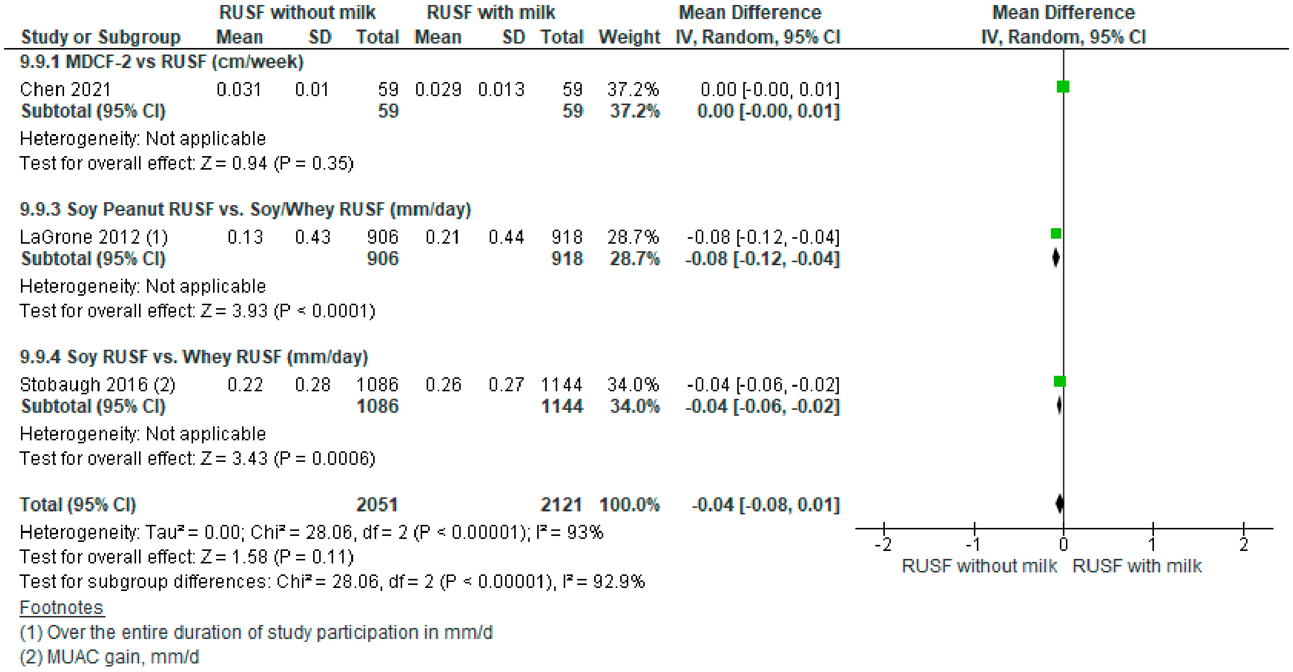

Footnotes
(1) Over the entire duration of study participation in mm/d
(2) MUAC gain, mm/d

Supplementary Figure S66. Forest plot of RUSF without animal protein vs RUSF with animal protein - Outcome: Weight gain

Study or Subgroup	RUSF without milk Mean	SD	Total	RUSF with milk Mean	SD	Total	Weight	Mean Difference IV, Random, 95% CI
**9.7.3 Soy Peanut RUSF vs. Soy/Whey RUSF**								
LaGrone 2012 (1)	3.4	2.6	906	3.6	2.8	918	33.1%	-0.20 [-0.45, 0.05]
**Subtotal (95% CI)**			**906**			**918**	**33.1%**	**-0.20 [-0.45, 0.05]**
Heterogeneity: Not applicable								
Test for overall effect: Z = 1.58 (P = 0.11)								
**9.7.4 Soy RUSF vs. Whey RUSF**								
Stobaugh 2016 (2)	2.79	2.16	1086	2.95	2.04	1144	66.9%	-0.16 [-0.33, 0.01]
**Subtotal (95% CI)**			**1086**			**1144**	**66.9%**	**-0.16 [-0.33, 0.01]**
Heterogeneity: Not applicable								
Test for overall effect: Z = 1.80 (P = 0.07)								
**Total (95% CI)**			**1992**			**2062**	**100.0%**	**-0.17 [-0.32, -0.03]**
Heterogeneity: Tau² = 0.00; Chi² = 0.07, df = 1 (P = 0.80); I² = 0%								
Test for overall effect: Z = 2.38 (P = 0.02)								
Test for subgroup differences: Chi² = 0.07, df = 1 (P = 0.80), I² = 0%								

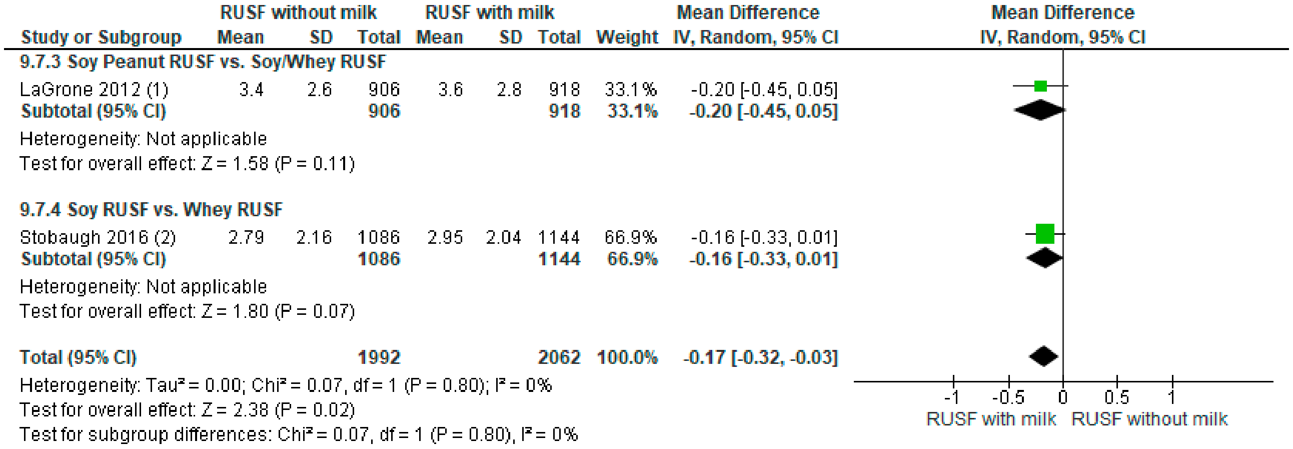

Footnotes
(1) At first 4 weeks on enrollment in g/kg/day
(2) Weight gain from enrollment to final visit in g/kg/day

Supplementary Figure S67. Forest plot of RUSF without animal protein vs RUSF with animal protein - Outcome: Height gain

Study or Subgroup	RUSF without milk Mean	SD	Total	RUSF with milk Mean	SD	Total	Weight	Mean Difference IV, Random, 95% CI
**9.8.2 Soy Peanut RUSF vs. Soy/Whey RUSF**								
LaGrone 2012 (1)	0.13	0.44	906	0.15	0.47	918	24.3%	-0.02 [-0.06, 0.02]
**Subtotal (95% CI)**			**906**			**918**	**24.3%**	**-0.02 [-0.06, 0.02]**
Heterogeneity: Not applicable								
Test for overall effect: Z = 0.94 (P = 0.35)								
**9.8.3 Soy RUSF vs. Whey RUSF**								
Stobaugh 2016 (2)	0.29	0.29	1086	0.3	0.28	1144	75.7%	-0.01 [-0.03, 0.01]
**Subtotal (95% CI)**			**1086**			**1144**	**75.7%**	**-0.01 [-0.03, 0.01]**
Heterogeneity: Not applicable								
Test for overall effect: Z = 0.83 (P = 0.41)								
**Total (95% CI)**			**1992**			**2062**	**100.0%**	**-0.01 [-0.03, 0.01]**
Heterogeneity: Tau² = 0.00; Chi² = 0.17, df = 1 (P = 0.68); I² = 0%								
Test for overall effect: Z = 1.18 (P = 0.24)								
Test for subgroup differences: Chi² = 0.17, df = 1 (P = 0.68), I² = 0%								

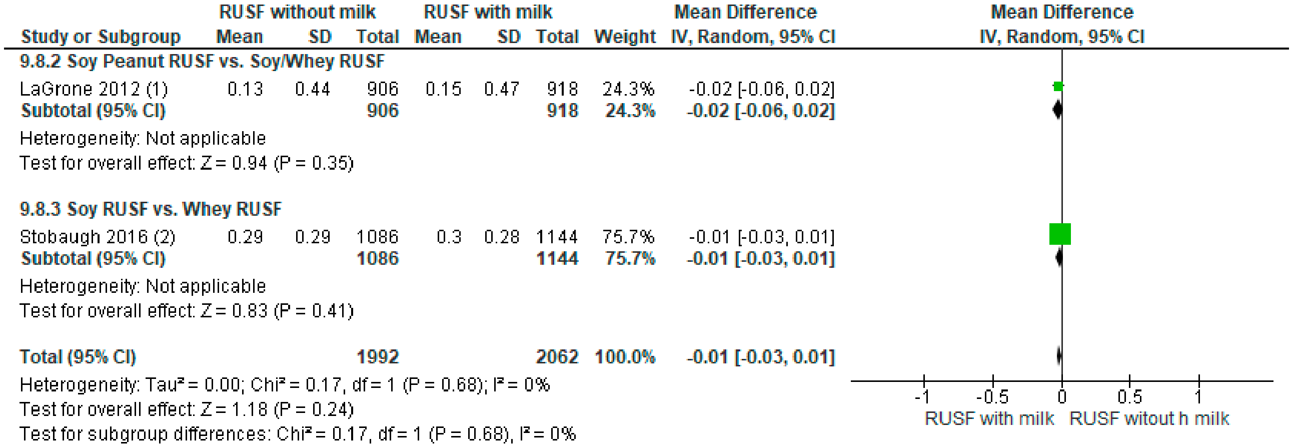

Footnotes
(1) over the entire duration of study participation in mm/d
(2) Length gain from enrollment to final visit, mm/d

Supplementary Figure S68. Forest plot of RUSF without animal protein vs RUSF with animal protein - Outcome: Sustained recovery

Study or Subgroup	RUSF without milk Events	Total	RUSF with milk Events	Total	Weight	Risk Ratio M-H, Random, 95% CI
**9.3.1 Soy Peanut RUSF vs. Soy/Whey RUSF**						
LaGrone 2012 (1)	382	647	447	667	100.0%	0.88 [0.81, 0.96]
**Subtotal (95% CI)**		**647**		**667**	**100.0%**	**0.88 [0.81, 0.96]**
Total events	382		447			
Heterogeneity: Not applicable						
Test for overall effect: Z = 2.98 (P = 0.003)						
Test for subgroup differences: Not applicable						

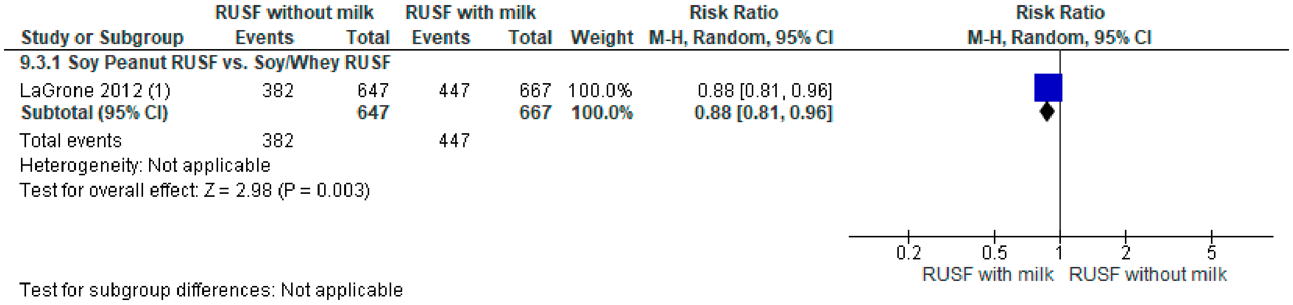

Footnotes
(1) Sustained recovery at 12 months

Supplementary Figure S69. Forest plot of RUSF without animal protein vs RUSF with animal protein - Outcome: Non-response

	Lipid based products		Lipid based products			Risk Ratio
**Study or Subgroup**	**Events**	**Total**	**Events**	**Total**	**Weight**	**M-H, Random, 95% CI**
**9.13.2 Soy Peanut RUSF vs. Soy/Whey RUSF**						
LaGrone 2012	5	906	8	918	6.7%	0.63 [0.21, 1.93]
**Subtotal (95% CI)**		**906**		**918**	**6.7%**	**0.63 [0.21, 1.93]**
Total events	5		8			
Heterogeneity: Not applicable						
Test for overall effect: Z = 0.80 (P = 0.42)						
**9.13.3 Soy RUSF vs. Whey RUSF**						
Stobaugh 2016 (1)	52	1086	49	1144	56.7%	1.12 [0.76, 1.64]
**Subtotal (95% CI)**		**1086**		**1144**	**56.7%**	**1.12 [0.76, 1.64]**
Total events	52		49			
Heterogeneity: Not applicable						
Test for overall effect: Z = 0.57 (P = 0.57)						
**9.13.5 Soy/Peanut vs. Milk/Peanut**						
Matilsky 2009 (2)	29	450	35	465	36.6%	0.86 [0.53, 1.38]
**Subtotal (95% CI)**		**450**		**465**	**36.6%**	**0.86 [0.53, 1.38]**
Total events	29		35			
Heterogeneity: Not applicable						
Test for overall effect: Z = 0.64 (P = 0.52)						
**Total (95% CI)**		**2442**		**2527**	**100.0%**	**0.98 [0.73, 1.30]**
Total events	86		92			

Heterogeneity: Tau² = 0.00; Chi² = 1.36, df = 2 (P = 0.51); I² = 0%
Test for overall effect: Z = 0.16 (P = 0.87)
Test for subgroup differences: Chi² = 1.36, df = 2 (P = 0.51), I² = 0%

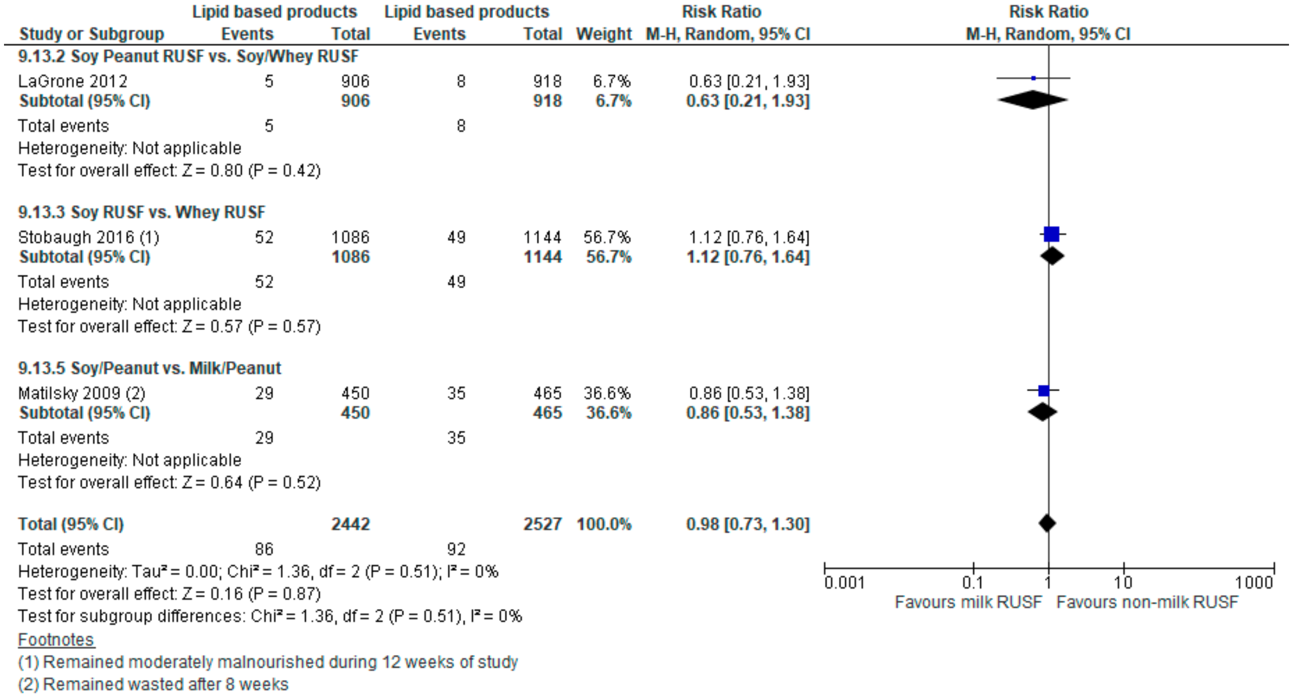

Footnotes
(1) Remained moderately malnourished during 12 weeks of study
(2) Remained wasted after 8 weeks

Supplementary Figure S70. Forest plot of RUSF without animal protein vs RUSF with animal protein - Outcome: Time to recovery

	RUSF without milk			RUSF with milk				Mean Difference
**Study or Subgroup**	**Mean**	**SD**	**Total**	**Mean**	**SD**	**Total**	**Weight**	**IV, Random, 95% CI**
**9.6.1 Soy Peanut RUSF vs. Soy/Whey RUSF**								
LaGrone 2012 (1)	22.5	14.2	906	22.6	15	918	55.4%	-0.10 [-1.44, 1.24]
**Subtotal (95% CI)**			**906**			**918**	**55.4%**	**-0.10 [-1.44, 1.24]**
Heterogeneity: Not applicable								
Test for overall effect: Z = 0.15 (P = 0.88)								
**9.6.2 Soy RUSF vs. Whey RUSF**								
Stobaugh 2016 (2)	30.4	20.1	874	29.3	19	960	30.9%	1.10 [-0.69, 2.89]
**Subtotal (95% CI)**			**874**			**960**	**30.9%**	**1.10 [-0.69, 2.89]**
Heterogeneity: Not applicable								
Test for overall effect: Z = 1.20 (P = 0.23)								
**9.6.4 Soy/Peanut vs. Milk/Peanut**								
Matilsky 2009 (3)	23.3	20.82	450	23.3	20.82	465	13.7%	0.00 [-2.70, 2.70]
**Subtotal (95% CI)**			**450**			**465**	**13.7%**	**0.00 [-2.70, 2.70]**
Heterogeneity: Not applicable								
Test for overall effect: Z = 0.00 (P = 1.00)								
**Total (95% CI)**			**2230**			**2343**	**100.0%**	**0.28 [-0.71, 1.28]**

Heterogeneity: Tau² = 0.00; Chi² = 1.15, df = 2 (P = 0.56); I² = 0%
Test for overall effect: Z = 0.56 (P = 0.58)
Test for subgroup differences: Chi² = 1.15, df = 2 (P = 0.56), I² = 0%

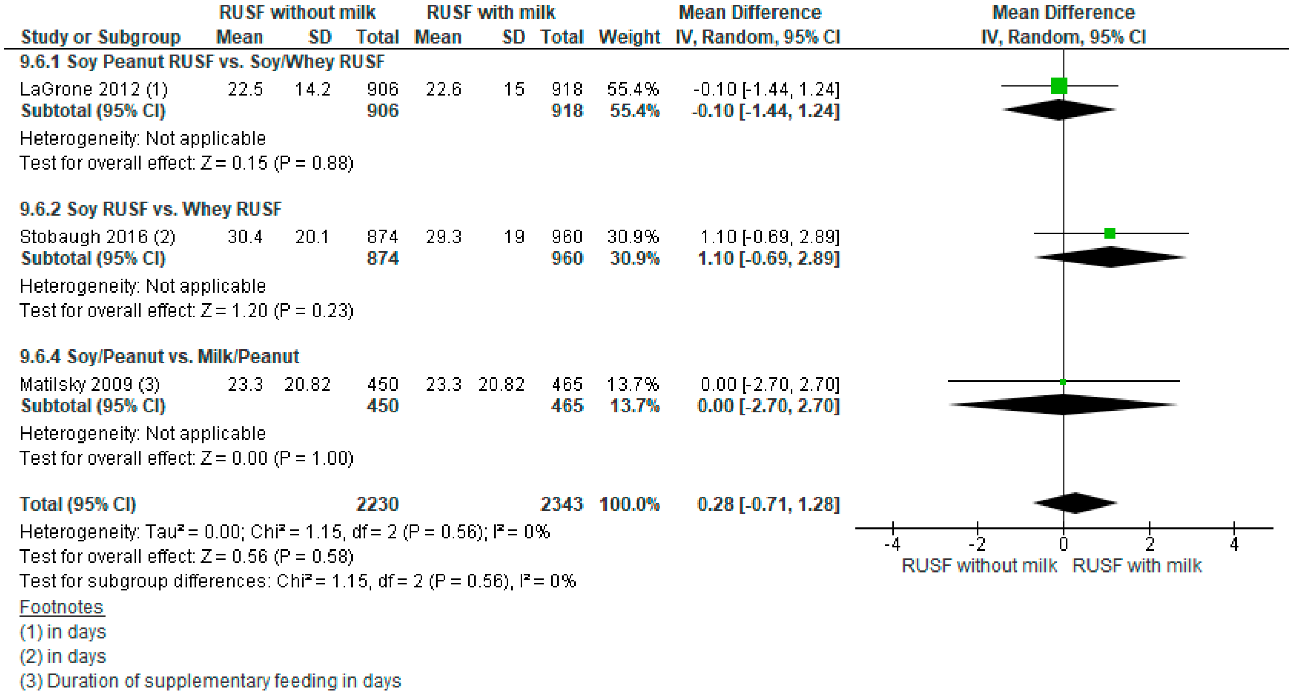

Footnotes
(1) in days
(2) in days
(3) Duration of supplementary feeding in days

Supplementary Figure S71. Forest plot of RUSF without animal protein vs RUSF with animal protein - Outcome: Relapse

Study or Subgroup	Lipid based products Events	Total	Lipid based products Events	Total	Weight	Risk Ratio M-H, Random, 95% CI
**9.4.2 Soy Peanut RUSF vs. Soy/Whey RUSF**						
LaGrone 2012 (1)	192	647	159	667	100.0%	1.24 [1.04, 1.49]
**Subtotal (95% CI)**		**647**		**667**	**100.0%**	**1.24 [1.04, 1.49]**
Total events	192		159			
Heterogeneity: Not applicable						
Test for overall effect: Z = 2.38 (P = 0.02)						

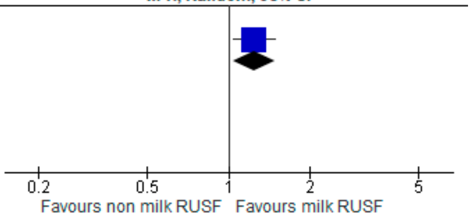

Footnotes
(1) Defined as WHZ <-2, but >= -3 and MUAC <12.5 cm; children who recovered from MAM 12 mo after enrollment plus who deteriorated to SAM

Supplementary Figure S72: Forest plot of protein-optimised RUSF vs RUSF - Dichotomous outcome: Anthropometric recovery rate, deterioration to SAM and non-response

Study or Subgroup	Protein-optimised RUSF Events	Total	Control RUSF Events	Total	Weight	Risk Ratio M-H, Random, 95% CI
**4.13.1 Recovery rate (number of children who recovered)**						
Roediger 2020 (1)	759	860	766	877	100.0%	1.01 [0.98, 1.05]
**Subtotal (95% CI)**		**860**		**877**	**100.0%**	**1.01 [0.98, 1.05]**
Total events	759		766			
Heterogeneity: Not applicable						
Test for overall effect: Z = 0.58 (P = 0.56)						
**4.13.2 Deterioration to severe wasting (number of children who develeoped SAM)**						
Roediger 2020 (2)	39	860	45	877	100.0%	0.88 [0.58, 1.34]
**Subtotal (95% CI)**		**860**		**877**	**100.0%**	**0.88 [0.58, 1.34]**
Total events	39		45			
Heterogeneity: Not applicable						
Test for overall effect: Z = 0.58 (P = 0.56)						
**4.13.5 Non-response (number of children who did not recover)**						
Roediger 2020 (3)	38	860	40	877	100.0%	0.97 [0.63, 1.50]
**Subtotal (95% CI)**		**860**		**877**	**100.0%**	**0.97 [0.63, 1.50]**
Total events	38		40			
Heterogeneity: Not applicable						
Test for overall effect: Z = 0.14 (P = 0.89)						

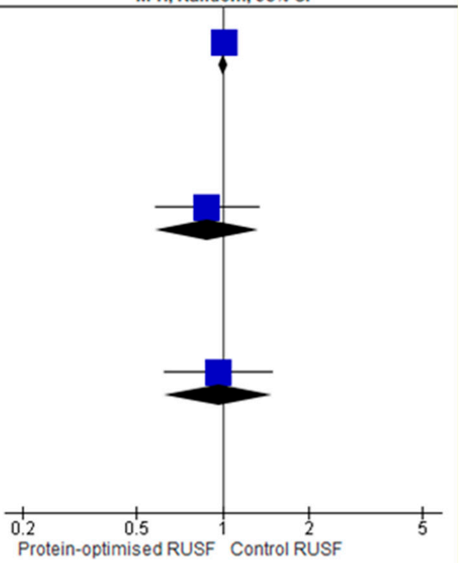

Footnotes
(1) Achieving a MUAC greater than or equal to 12.5 cm or a WHZ greater than or equal to −2 within 12 weeks of treatment
(2) Developed SAM
(3) Remained MAM in 12 weeks

Supplementary Figure S73: Forest plot of protein-optimised RUSF vs RUSF - Dichotomous outcome: MUAC gain, Weight gain, Time to recovery

Study or Subgroup	Protein-optimised RUSF Mean	SD	Total	Control RUSF Mean	SD	Total	Weight	Mean Difference IV, Random, 95% CI
**4.6.1 MUAC gain (mm/day)**								
Roediger 2020 (1)	2.53	3	860	2.47	3	877	100.0%	0.06 [-0.22, 0.34]
**Subtotal (95% CI)**			**860**			**877**	**100.0%**	**0.06 [-0.22, 0.34]**
Heterogeneity: Not applicable								
Test for overall effect: Z = 0.42 (P = 0.68)								
**4.6.2 Weight gain (g/kg/day)**								
Roediger 2020 (2)	2.44	2.3	860	2.41	2.5	877	100.0%	0.03 [-0.20, 0.26]
**Subtotal (95% CI)**			**860**			**877**	**100.0%**	**0.03 [-0.20, 0.26]**
Heterogeneity: Not applicable								
Test for overall effect: Z = 0.26 (P = 0.79)								
**4.6.3 Time to recovery (days)**								
Roediger 2020 (3)	28.8	19.4	860	28.6	18.7	877	100.0%	0.20 [-1.59, 1.99]
**Subtotal (95% CI)**			**860**			**877**	**100.0%**	**0.20 [-1.59, 1.99]**
Heterogeneity: Not applicable								
Test for overall effect: Z = 0.22 (P = 0.83)								

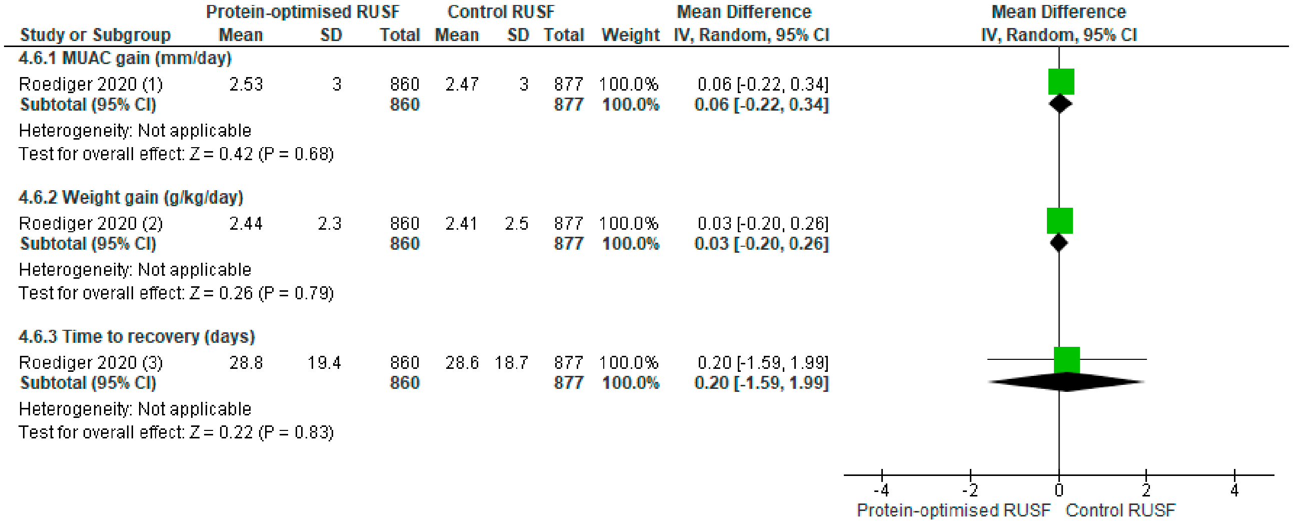

Footnotes
(1) average MUAC gain mm
(2) Rate of weight gain at outcome assessment in g/kg/day
(3) in days

Supplementary Figure S74: Forest plot of RUTF vs alternative RUTF - Outcome: Anthropometric recovery rate

Study or Subgroup	Alternative RUTF Events	Total	Standard RUTF Events	Total	Weight	Risk Ratio M-H, Random, 95% CI
**4.16.2 Standard RUTF vs alternative RUTF**						
Kohlmann 2019 (1)	398	426	386	443	100.0%	1.07 [1.03, 1.12]
**Subtotal (95% CI)**		**426**		**443**	**100.0%**	**1.07 [1.03, 1.12]**
Total events	398		386			
Heterogeneity: Not applicable						
Test for overall effect: Z = 3.12 (P = 0.002)						

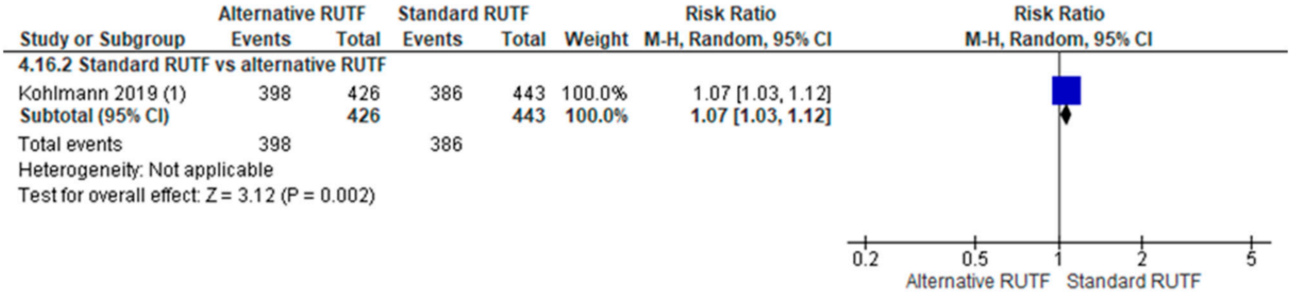

Footnotes
(1) Proportion of children having achieved either WLZ >-2 or MUAC >12.4 cm at any point during the treatment

Supplementary Figure S75: Forest plot of RUTF vs alternative RUTF - Anthropometric outcome: Weight and MUAC gain

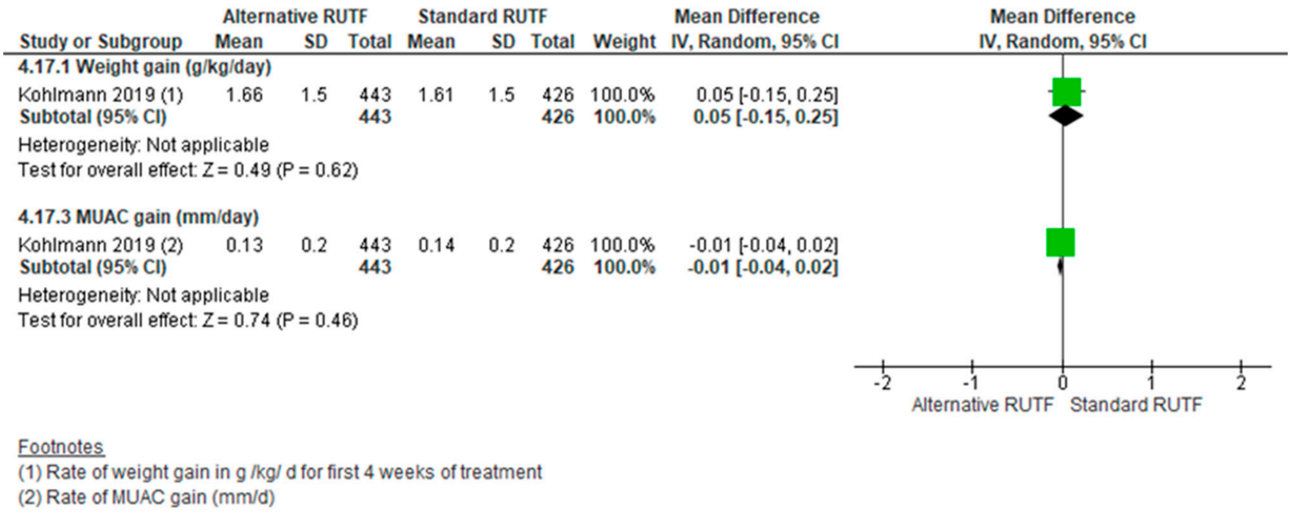

Supplementary Figure S76. Meta-regression of the relationship between RUTF dose and anthropometric recovery[1]

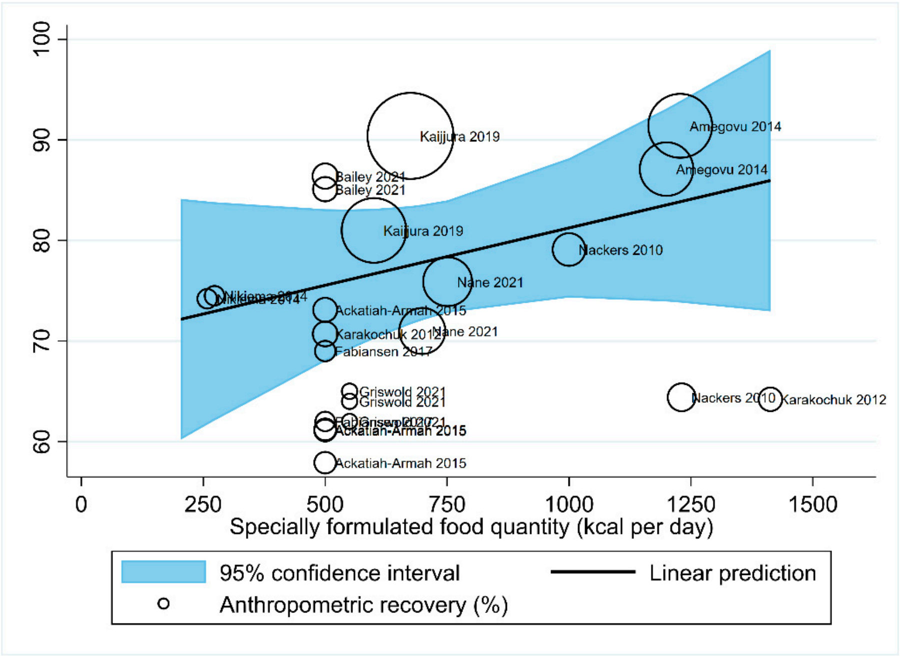

[1]The bubble sizes are proportional to the inverse of the study-level variance for anthropometric recovery.

Supplementary Figure S77. Meta-regression of the relationship between RUTF dose and MUAC gain.

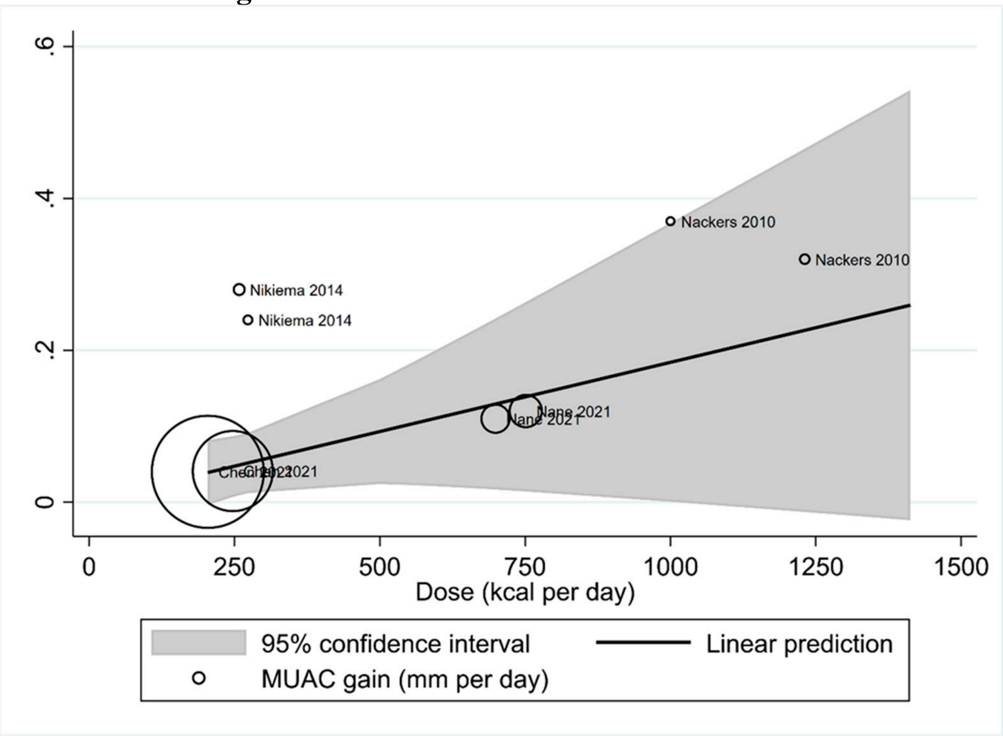

The bubble sizes are proportional to the inverse of the study-level variance for MUAC gain.

Supplementary Figure S78. Meta-regression of the relationship between fixed RUTF duration and anthropometric recovery

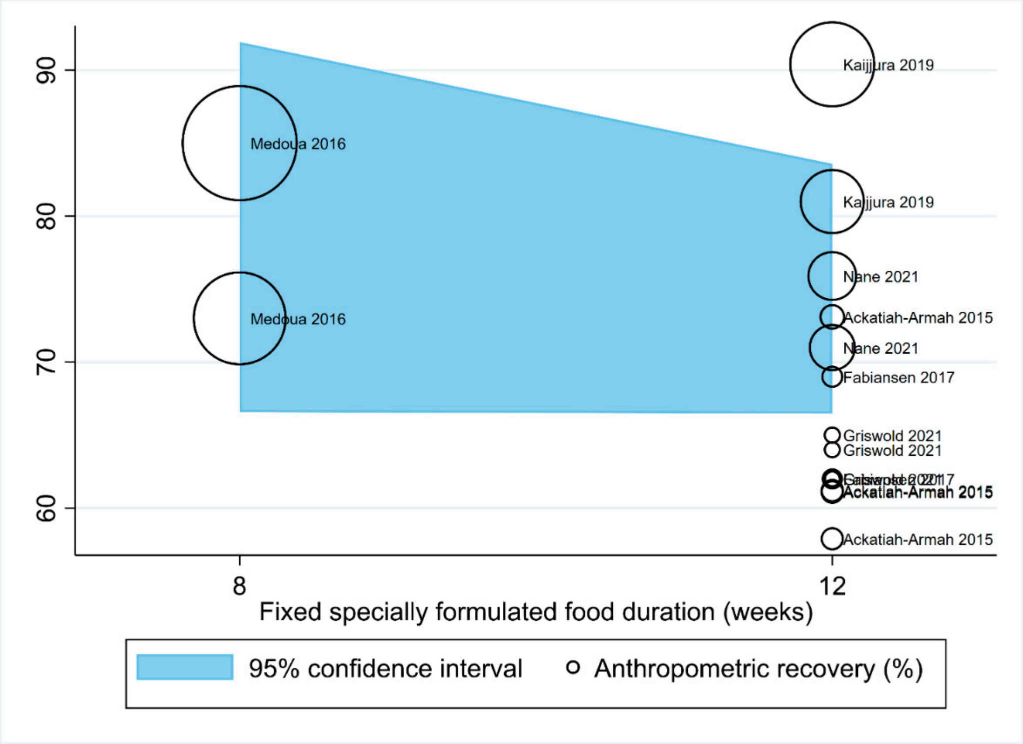

The bubble sizes are proportional to the inverse of the study-level variance for anthropometric recovery.
