# Supplementary material for: Effectiveness of Dietary Management for Moderate Wasting among Children > 6 Months of Age—A Systematic Review and Meta-Analysis Exploring Different Types, Quantities, and Durations
Source: Nutrients. 2023 Feb 21;15(5):1076. doi: 10.3390/nu15051076 (PMC10005276; doi:10.3390/nu15051076)
Supplement: Supplementary file 1 [file nutrients-15-01076-s001.zip › Annex 4. Supplementary Tables.pdf]

Annex 3. Supplementary tables

Supplementary Table S1. Search strategy

Children		Wasting		Intervention
“Infant” [MeSH] OR “Child” [MeSH] OR<br><br>Infant* OR toddler* OR baby OR babies OR preschool OR kindergarten OR under-5* OR “under 5*” OR under-five OR “under five” OR kid OR kids OR paediatr* OR pediatr*	AND	“Malnutrition” [MeSH] OR “infant nutrition disorders” [MeSH] OR “protein-energy malnutrition” [MeSH] OR “wasting syndrome” [MeSH] OR malnourish* OR undernutrition OR wasting OR “Acute Malnutrition”[MeSH] OR MAM OR undernutrition OR under-nutrition OR underweight OR wast* OR “weight for height” OR “weight-for-height” OR “weight for length” OR “weight-for-length” OR “weight for age” OR “weight-for-age” OR “mid-upper arm circumference” OR “mid upper arm circumference” OR MUAC	AND	“Food” [MeSH] OR “infant food” [MeSH] OR “food, fortified” [MeSH] OR “food, fomulated” [MeSH] OR “dietary supplements” [MeSH] OR “dietary fat*” [MeSH] OR “Milk Proteins” [Mesh] OR “fortified food*” OR diet* OR supplement* OR “ready to use therapeutic food” OR RUTF OR “ready to use supplementary food” OR RUSF OR “ready to use food” OR RUF OR f1000 OR F75 OR CTC OR “micronutrient* supplement*” OR fat* OR “dietary fat*” OR “dietary protein*” OR FBF OR “corn soy” OR “Wheat soy* blend*” OR “Rice milk blend*” OR “Milk rice blend*” OR “Pea wheat blend*” OR “Cereal pulse blend*” OR “Lipid-based nutrient supplement*” OR Nutributter OR CSB OR Supercereal* OR (diet* adj3 supplement*) OR (supplement* adj3 food*) OR "ready to use" OR ready-to-use OR RUTF OR RUSF OR RUF OR F100 OR F75 OR CTC OR FBF OR CMAM OR “community based management” OR “community-based management” OR “integrated community case management” OR ICCM OR “integrated management of acute malnutrition” OR IMAM OR “inpatient management” OR “in-patient management” OR IMCI OR IMNCI OR “facility based management” OR “facility-based management” OR “supplementary feeding program*” OR SFP

Supplementary Table S2. Evidence Profile for Comparison 1c. Enhanced commercially produced and/or imported FBFs vs LNS[1]

Population: Children with moderate wasting

Intervention: Enhanced FBFs

Comparison: LNS[2]

Settings: Rural Mali, rural Burkina Faso, Sierra Leone, Malawi, Cameroon

Certainty assessment							*№ of patients*		*Effect*		*Certainty*	*Importance*
№ of studies	*Study design*	*Risk of bias*	*Inconsistency*	*Indirectness*	*Imprecision*	*Other considerations*	*Enhanced commercially produced and/or imported FBFs*	*LNS*	*Relative (95% CI)*	*Absolute (95% CI)*		
Anthropometric recovery [3]												
6	randomised trials	serious[a]	not serious	not serious	serious[b]	none	3205/4660 (68.8%)	3425/4461 (76.8%)	RR 0.96 (0.93 to 1.00)	31 fewer per 1,000 (from 54 fewer to 0 fewer)	⨁⨁◯◯ Low	CRITICAL
Difference in WHZ at the end of the intervention period[4,5]												
3	randomised trials	serious[a]	not serious	not serious	not serious	none	1271 Mean (SD): -1.46 (0.72)	2199 Mean (SD):-1.36 (0.72)	-	MD 0.09 lower (0.14 lower to 0.04 lower)	⨁⨁⨁◯ Moderate	CRITICAL
Difference in WAZ at the end of the intervention period – not reported												
-	-	-	-	-	-	-	-	-	-	-	-	CRITICAL

Difference in HAZ at the end of the intervention period – not reported												
-	-	-	-	-	-	-	-	-	-	-	-	CRITICAL
MUAC gain in cm over the intervention period[5]												
3	randomised trials	serious[a]	not serious	not serious	serious[c]	none	1271<br><br>Mean (SD): 0.9 (3.2)	2199<br><br>Mean (SD): 1.16 (3.2)	-	MD 0.26 lower<br><br>(0.48 lower to 0.03 lower)	⨁⨁◯◯<br><br>Low	CRITICAL
Weight gain in g/kg/day												
3	randomised trials	serious[a]	not serious	not serious	serious[d]	none	1271<br><br>Mean (SD): 2.49 (2.93)	2199<br><br>Mean (SD):2.84 (2.9)	-	MD 0.36 lower<br><br>(0.56 lower to 0.15 lower)	⨁⨁◯◯<br><br>Low	CRITICAL
Height gain in cm over the intervention period[5]												
2	randomised trials	serious[a]	not serious	not serious	not serious	none	1230<br><br>Mean (SD): 2.33 (2.5)	2159<br><br>Mean (SD): 2.35 (2.5)	-	MD 0.02 lower<br><br>(0.2 lower to 0.15 higher)	⨁⨁⨁◯<br><br>Moderate	CRITICAL
Sustained recovery[6]												
1	randomised trials	serious[a]	not serious	not serious	serious[e]	none	405/653 (62.0%)	828/1314 (63.0%)	RR 0.99<br><br>(0.87 to 1.11)	6 fewer per 1,000<br><br>(from 82 fewer to 69 more)	⨁⨁◯◯<br><br>Low	IMPORTANT
Deterioration to severe wasting[7]												
5	randomised trials	serious[a]	not serious	not serious	not serious	none	537/4074 (13.2%)	409/3625 (11.3%)	RR 1.03<br><br>(0.86 to 1.23)	3 more per 1,000<br><br>(from 12 fewer to 20 more)	⨁⨁⨁◯<br><br>Moderate	IMPORTANT

Time to recovery in days												
4	randomised trials	serious[a]	serious[f]	not serious	Serious[g]	none	1641<br><br>Mean (SD): 36.38 (52.79)	2624<br><br>Mean (SD): 33.18 (52.79)	-	MD 3.2 higher<br><br>(0.06 lower to 6.45 higher)	⊕◯◯◯<br><br>Very low	IMPORTANT
Non-response[8]												
5	randomised trials	serious[a]	serious[h]	not serious	Serious[i]	none	398/2746 (14.5%)	412/3702 (11.1%)	RR 1.10<br><br>(0.81 to 1.50)	11 more per 1,000<br><br>(from 21 fewer to 56 more)	⊕⊕◯◯<br><br>Low	IMPORTANT
Relapse[9]												
1	randomised trials	Serious[j]	not serious	not serious	serious[k]	none	173/653 (26.5%)	351/1314 (26.7%)	RR 0.99<br><br>(0.81 to 1.22)	3 fewer per 1,000<br><br>(from 51 fewer to 59 more)	⊕⊕◯◯<br><br>Low	IMPORTANT

[1]CI: confidence interval; MD: mean difference; RR: risk ratio; WHZ: Weight-for-Height Z-score; HAZ: Height-for-age Z-score; WAZ: Weight-for-age z-score; MUAX: Mid-upper arm circumference; [2]LNS was chosen as comparator because it is a common comparator across the 3 meta-analysis under comparison 1. [3] Out of the 6 studies included, 3 defined recovery from MAM as reaching a WHZ ≥−2 (Nikiema et al., 2014, Medoua et al., 2016; LaGrone et al., 2012); one study defined recovery as reaching a MUAC of ≥12.5cm (Griswold et al., 2021), one as reaching a WHZ≥−2 and MUAC ≥12.5cm (Fabiansen et al., 2017); one study defined recovery as reaching a WHZ≥−2 and MUAC ≥12.5cm at two consecutive follow-up visits (Ackatiah-Armah et al., 2014); [4]2 studies reported WHZ and HAZ at the end of the intervention while 1 reported change throughout the intervention period; [5] In two studies the intervention period was 12 weeks (Ackatiah-Armah et al., 2014, LaGrone et al., 2012) and in one study the intervention period was 8 weeks (Medoua et al., 2016);[6]sustained recovery at 12 months; [7]Devleoping SAM during the intervention period based either on WHZ<-3, MUAC<11.5cm and oedema criteria (Fabiansen et al., 2017, Ackatiah-Armah et al., 2014), MUAC<11.5 and oedema criteria (Griswold et al, 2021) or WHZ<-3 and oedema criteria (Nikiema et al., 2014, Medoua et al., 2016; LaGrone et al., 2012); [8]remained MAM at the end of the intervention period of 12 weeks (Fabiansen et al., 2017, Nikiema et al., 2014, Ackatiah-Armah et al., 2015, LaGrone et al., 2012) or 8 weeks (Medoua et al., 2016); [9]Relapsed to MAM during a 12 months follow-up period.

Explanations for Evidence Grade

a. Serious risk of bias: majority or all information is from studies at high or unclear overall risk of bias.
b. Serious imprecision: Absolute effect crosses the null threshold, effect ranges from small harm to no difference.
c. Serious imprecision: 95% CI around the absolute effect does not cross the null, however effect ranges from moderate to trivial harm.

d. Serious imprecision: 95% CI around the absolute effect does not cross the null threshold, however the 95% CI ranges from a trivial to a small harm
e. Serious imprecision: 95% CI around the absolute effect crosses the null threshold and effect ranges from small benefit to small harms.
f. Considerable inconsistency: I2=93%
g. Serious imprecision: the 95% CI around the absolute effect crosses the null, ranges include trivial benefit to appreciable harm
h. Not necessary to double downgrade for inconsistency and imprecision.
i. Serious imprecision: 95% CI around the absolute effect crosses the null threshold, effect ranges from trivial benefit to small harms.

Supplementary Table S3. Evidence Profile for comparison 1.b. CSB vs LNS[1]

Population: 6-60 months old children with moderate wasting

Intervention: Corn soy blend

Comparison: LNS

Settings: Rural Niger, Malawi and Ethiopia

Certainty assessment							№ of patients		Effect		Certainty	Importance
№ of studies	Study design	Risk of bias	Inconsistency	Indirectness	Imprecision	Other considerations	Corn Soy Blend (CSB)	Lipid based nutrient supplements (LNS)	Relative (95% CI)	Absolute (95% CI)		
**Anthropometric recovery at 8-16 weeks[3]**												
3	randomised trials	serious[a]	not serious	not serious	serious[b]	none	957/1433 (66.8%)	1164/1505 (77.3%)	**RR 0.89** (0.85 to 0.94)	**85 fewer per 1,000** (from 116 fewer to 46 fewer)	⊕⊕◯◯ Low	CRITICAL
**WHZ at discharge (after up to 8 weeks of treatment)**												
1	randomised trials	not serious	not serious	not serious	serious[b]	none	915<br>Mean z-score:- -1.8	447<br>Mean z-score: -1.65	-	MD 0.15 lower (0.25 lower to 0.5 lower)	⊕⊕⊕◯ Moderate	CRITICAL

Certainty assessment							№ of patients		Effect		Certainty	Importance
№ of studies	Study design	Risk of bias	Inconsistency	Indirectness	Imprecision	Other considerations	Corn Soy Blend (CSB)	Lipid based nutrient supplements (LNS)	Relative (95% CI)	Absolute (95% CI)		
**HAZ at discharge (after up to 8 weeks of treatment)**												
1	randomised trials	not serious	not serious	not serious	serious[b]	none	447<br><br>Mean zscore: -2.8	915<br><br>Mean zscore: -2.65	-	MD 0.15 lower (0.35 lower to 0.05 higher)	⨁⨁⨁◯ Moderate	CRITICAL
**MUAC gain (mm/day)**												
1	randomised trials	serious[c]	not serious	not serious	not serious	none	150<br><br>Mean (SD): 0.32 (0.24)	162<br><br>Mean (SD): 0.37 (0.29)	-	MD 0.05 lower (0.11 lower to 0.01 higher)	⨁⨁⨁◯ Moderate	CRITICAL
**Weight gain (g/kg/day) in the first two weeks of treatment**												
1	randomised trials	serious[c]	not serious	not serious	not serious	none	217<br><br>Mean (SD): 3.48 (4.32)	204<br><br>Mean (SD): 5.34 (4.15)	-	MD 1.86 lower (2.67 lower to 1.05 lower)	⨁⨁⨁◯ Moderate	CRITICAL
**Height gain – not measured**												
-	-	-	-	-	-	-	-	-	-	-	-	CRITICAL
**Sustained recovery – not measured**												
-	-	-	-	-	-	-	-	-	-	-	-	IMPORTANT
**Deterioration to severe wasting during the intervention period[4]**												

Certainty assessment							№ of patients		Effect		Certainty	Importance
№ of studies	Study design	Risk of bias	Inconsistency	Indirectness	Imprecision	Other considerations	Corn Soy Blend (CSB)	Lipid based nutrient supplements (LNS)	Relative (95% CI)	Absolute (95% CI)		
3	randomised trials	serious[a]	not serious	not serious	serious[d]	none	88/1433 (6.1%)	100/1505 (6.6%)	**RR 1.15** (0.73 to 1.84)	**10 more per 1,000** (from 18 fewer to 56 more)	⊕⊕◯◯ Low	IMPORTANT
**Non-response[5]**												
3	randomised trials	serious[a]	serious[f]	not serious	serious[e]	none	166/1037 (16.0%)	301/1901 (15.8%)	**RR 1.27** (0.68 to 2.36)	**43 more per 1,000** (from 51 fewer to 215 more)	⊕◯◯◯ Very low	IMPORTANT
**Time to recovery (weeks)**												
1	randomised trials	serious[c]	not serious	not serious	serious[e]	none	152<br><br>Mean (SD): 8 (10.5)	170<br><br>Mean (SD): 7.3 (10.5)	-	MD 0.7 higher (1.6 lower to 3 higher)	⊕⊕◯◯ Low	IMPORTANT
**Relapse**												
1	randomised trials	serious[c]	not serious	not serious	serious[g]	none	33/152 (21.7%)	33/170 (19.4%)	**RR 1.12** (0.73 to 1.72)	**23 more per 1,000** (from 52 fewer to 140 more)	⊕⊕◯◯ Low	IMPORTANT

[1] **Abbreviations: CI:** confidence interval; **MD:** mean difference; **RR:** risk ratio; WHZ: Weight-for-Height Z-score; HAZ: Height-for-age Z-score; WAZ: Weight-for-age z-score; MUAC: Mid-upper arm circumference; [2] LNS was chosen as comparator across these three meta-analyses because it is a common comparator across the 3 meta-analysis not necessarily the "standard of care".[3] Recovery criteria varied across the 3 studies: Karakochuck et al. (2012) and Nackers et al (2010) defined recovery as WFH≥85% across 2 consecutive visits; Matilsky et al., (2009) defined recovery as reaching a WHZ≥-2.[4] The intervention period varied between 8 weeks (Matilsky et al.,2009) and 16 weeks (Nackers et al., 2010; Karakochuck et al., 2012);[5] Children who remained MAM during the intervention period;

Explanations for evidence GRADE

a. Serious risk of bias: Two out of three studies have a high risk of bias
b. Serious imprecision: Absolute effects cross the null threshold, effects range from trivial benefit to small harm
c. Serious imprecision: Absolute effects cross the null threshold, effects range from very small benefit to moderate harms.
d. Serious risk of bias: High risk of bias in the Nackers et al., 2010 study
e. Serious imprecision: Absolute effects cross the null threshold, effects range from small to trivial difference
f. Serious inconsistency: Downgraded by one level to variation in point estimates (I2=89%)

g. Serious imprecision: Absolute effect crosses the null, effect ranges include appreciable benefit and harm.
h. Serious imprecision: Absolute effect crosses the null, effect ranges from small benefit to moderate harms.

j. Serious risk of bias: all information is from a study with some concerns for overall risk of bias
k. Serious imprecision: 95% CI around the absolute effect crosses the null threshold and effect range from small harms to small benefits.

Supplementary Table S4. Evidence Profile for Comparison 1c (locally produced FBF compared to LNS)[1,2]

Population: 6-35 months old children with moderate wasting

Intervention: Locally produced FBFs (Misola and LMF)

Comparison: RUSF

Settings: Rural Mali

Certainty assessment							№ of patients		Effect		Certainty	Importance
№ of studies	Study design	Risk of bias	Inconsistency	Indirectness	Imprecision	Other considerations	Locally produced FBFs	LNS	Relative (95% CI)	Absolute (95% CI)		
**Anthropometric recovery[3]**												
1	randomised trials	serious[a]	not serious	not serious	not serious[b]	None	337/587 (57.4%)	234/335 (69.9%)	RR 0.82 (0.74 to 0.91)	126 fewer per 1,000 (from 182 fewer to 63 fewer)	⨁⨁◯◯ Low	CRITICAL
**Change in WHZ over 12 weeks of treatment**												
1	randomised trials	serious[a]	not serious	not serious	Serious[c]	None	587<br>Mean (SD): 0.6 (1.04)	335<br>Mean (SD): 0.94 (1.04)	-	MD 0.34 lower (0.48 lower to 0.2 lower)	⨁⨁◯◯ Low	CRITICAL
**Change in WAZ over 12 weeks of treatment – not reported**												
-	-	-	-	-	-	-	-	-	-	-	-	CRITICAL
**Change in HAZ over 12 weeks of treatment – not reported**												

Certainty assessment							№ of patients		Effect		Certainty	Importance
№ of studies	Study design	Risk of bias	Inconsistency	Indirectness	Imprecision	Other considerations	Locally produced FBFs	LNS	Relative (95% CI)	Absolute (95% CI)		
-	-	-	-	-	-	-	-	-	-	-	-	CRITICAL
**Change in MUAC in cm over 12 weeks of treatment**												
1	randomised trials	serious[a]	not serious	not serious	serious[d]	None	587<br>Mean (SD): 0.75 (0.89)	335<br>Mean (SD): 1.1 (0.89)	-	MD 0.35 lower (0.47 lower to 0.23 lower)	⊕⊕◯◯<br>Low	CRITICAL
**Weight gain in kg over 12 weeks of treatment**												
1	randomised trials	serious[a]	not serious	not serious	not serious	None	587<br>Mean (SD): 0.87 (0.75)	335<br>Mean (SD): 1.16 (0.75)	-	MD 0.29 lower (0.39 lower to 0.19 lower)	⊕⊕⊕◯<br>Moderate	CRITICAL
**Height gain in cm over 12 weeks of treatment**												
1	randomised trials	serious[a]	not serious	not serious	not serious	None	587<br>Mean (SD): 2.6 (1.4)	335<br>Mean (SD): 2.9 (1.4)	-	MD 0.26 lower (0.45 lower to 0.06 lower)	⊕⊕⊕◯<br>Moderate	CRITICAL
**Sustained recovery – Not measured**												
-	-	-	-	-	-	-	-	-	-	-	-	IMPORTANT
**Deterioration to severe wasting – Data not reported by study arm**												
-		-	-	-	-	-	-	-	-	-	-	IMPORTANT
**Non-response**												

Certainty assessment							№ of patients		Effect		Certainty	Importance
№ of studies	Study design	Risk of bias	Inconsistency	Indirectness	Imprecision	Other considerations	Locally produced FBFs	LNS	Relative (95% CI)	Absolute (95% CI)		
1	randomised trials	serious[a]	not serious	not serious	serious[e]	None	220/587 (37.5%)	78/335 (23.3%)	**RR 1.61** (1.29 to 2.01)	**142 more per 1,000** (from 68 more to 235 more)	⨁⨁◯◯ Low	IMPORTANT
**Time to recovery in weeks**												
1	randomised trials	serious[a]	not serious	not serious	Serious[f]	None	350<br><br>Mean (SD): 9.3 (8.8)	245<br><br>Mean (SD): 5.9 (8.8)		**MD:** 3.4 higher (2.22 higher to 4.58 higher)	⨁⨁◯◯ Low	
**Relapse – not measured**												

Footnotes: [1]CI: confidence interval; MD: mean difference; RR: risk ratio; WHZ: Weight-for-Height Z-score; HAZ: Height-for-age Z-score; WAZ: Weight-for-age z-score; MUAC: Mid-upper arm circumference; [2]LNS was chosen as comparator across these three meta-analyses because it is a common comparator across the 3 meta-analysis not necessarily the "standard of care"; [3] Recovery from MAM defined as reaching WHZ >–2.0 and MUAC >12.5 cm during 2 consecutive follow-up visits.

Explanations for certainty of evidence

a. Serious Risk of bias: All information is from one trial with unclear overall risk of bias.
b. Serious imprecision: 95% CI around the absolute effect does not cross the null threshold, however the 95% CI includes both small to moderate harm
c. c. Serious imprecision: 95% CI around the absolute effect does not cross the null threshold, however 95% CI includes both trivial to small harm
d. d. Serious imprecision: 95% CI around the absolute effects does not cross the null threshold, however 95% CI includes both moderate to trivial harm.
e. Serious imprecision: 95% CI around the absolute effect does not cross the null threshold, however 95% CI includes both small to moderate harm
f. Serious imprecision: 95% CI around the absolute effect does not cross the null threshold, however 95% CI is large

Supplementary Table S5. Evidence Profile for comparison 2.a.1. (Enhanced commercially produced and/or imported FBFs vs locally produced FBFs)[1]

Population: Children with moderate wasting

Intervention: Enhanced FBFs

Comparison: Locally produced FBFs

Settings: Rural Mali, Uganda, Ethiopia

Certainty assessment							№ of patients		Effect		Certainty	Importance
№ of studies	Study design	Risk of bias	Inconsistency	Indirectness	Imprecision	Other considerations	Enhanced commercially produced and/or imported FBFs	Locally produced FBFs	Relative (95% CI)	Absolute (95% CI)		
**Recovery[2]**												
4	randomised trials	serious[a]	not serious	not serious	serious[b]	none	482/692 (69.7%)	649/943 (68.8%)	RR 0.96 (0.90 to 1.01)	28 fewer per 1,000 (from 69 fewer to 7 more)	⨁⨁◯◯ Low	CRITICAL
**WHZ at the end of the intervention period[3]**												
3	randomised trials	serious[a]	serious[c]	not serious	serious[d]	none	530<br>Mean (SD): -0.25 (2.2)	781<br>Mean (SD): -0.21 (2.2)	-	MD **0.04 lower** (0.29 lower to 0.2 higher)	⨁◯◯◯ Very low	CRITICAL
**WAZ at the end of the intervention period**												
1	randomised trials	serious[a]	not serious	not serious	serious[e]	none	100<br>Mean (SD): -2.2 (0.82)	104<br>Mean (SD): -1.94 (0.69)	-	MD **0.27 lower** (0.48 lower to 0.07 lower)	⨁⨁◯◯ Low	CRITICAL

Certainty assessment							№ of patients		Effect		Certainty	Importance
№ of studies	Study design	Risk of bias	Inconsistency	Indirectness	Imprecision	Other considerations	Enhanced commercially produced and/or imported FBFs	Locally produced FBFs	Relative (95% CI)	Absolute (95% CI)		
**HAZ at the end of the intervention period**												
2	randomised trials	serious [a]	not serious	not serious	very serious [f]	none	188<br>Mean (SD): -2.23	194<br>Mean (SD): -2.07	-	MD **0.17 lower** (0.4 lower to 0.07 higher)	⊕◯◯◯<br>Very low	CRITICAL
**MUAC gain in cm over the intervention period**												
2	randomised trials	serious [a]	serious [g]	not serious	not serious [h]	none	504<br>Mean (SD): 0.9	749<br>Mean (SD): 0.84	-	MD **0.06 higher** (0.13 lower to 0.25 higher)	⊕⊕◯◯<br>Low	CRITICAL
**Weight gain over the 12-week intervention period in kg**												
3	randomised trials	serious [a]	not serious	not serious	not serious [h]	none	604<br>Mean (SD): 1.13	853<br>Mean (SD): 1.01	-	MD **0.13 higher** (0.05 higher to 0.21 higher)	⊕⊕⊕◯<br>Moderate	CRITICAL
**Height gain over the 12-week intervention period in cm**												
3	randomised trials	serious [a]	serious [j]	not serious	not serious [h]	none	604<br>Mean (SD): 2.91	853<br>Mean (SD):2.87	-	MD **0.05 higher** (0.19 lower to 0.29 higher)	⊕⊕◯◯<br>Low	CRITICAL
**Sustained recovery – Not reported**												
-	-	-	-	-	-	-	-	-	-	-	-	-
**Deterioration to severe wasting[4]**												

Certainty assessment							№ of patients		Effect		Certainty	Importance
№ of studies	Study design	Risk of bias	Inconsistency	Indirectness	Imprecision	Other considerations	Enhanced commercially produced and/or imported FBFs	Locally produced FBFs	Relative (95% CI)	Absolute (95% CI)		
1	randomised trials	not serious	not serious	not serious	very serious[i]	none	4/162 (2.5%)	2/162 (1.2%)	RR 2.00 (0.37 to 10.77)	12 more per 1,000 (from 8 fewer to 121 more)	⊕⊕◯◯ Low	IMPORTANT
**Time to recovery (days)**												
2	randomised trials	serious [a]	very serious[k]	not serious	very serious[l]	none	369<br>Mean (SD): 51.6	512<br>Mean (SD): 61.6	-	MD **9.98 lower** (21.93 lower to 1.96 higher)	⊕◯◯◯ Very low	IMPORTANT
**Non-response**												
2	randomised trials	serious [a]	not serious	not serious	Serious[m]	none	125/430 (29.1%)	227/677 (33.5%)	**RR 0.91** (0.76 to 1.10)	**30 fewer per 1,000** (from 80 fewer to 34 more)	⊕⊕◯◯ Low	IMPORTANT
**Relapse – not measured**												
-	-	-	-	-	-	-	-	-	-	-	-	IMPORTANT

[1] Abbreviations used in the table: **CI:** confidence interval; **MD:** mean difference; **RR:** risk ratio; [2] Criteria for recovery across studies varied. Out of the 4 studies included, one defined recovery as having reached a WHZ> –2 and adding 10% of their admission weight for two consecutive visits (Amegovu et al., 2014), one as reaching a MUAC ≥12.5cm and/or WHZ ≥-2 without bipedal edema at the end of 12 weeks (Nane et al., 2021); one study defined recovery as reaching a WHZ >−2.0 (Kajjura et al., 2019) and one as reaching a WHZ≥−2 and MUAC ≥12.5cm at two consecutive follow-up visits (Ackatiah-Armah et al., 2014); [3] One study measured change in WHZ throughout the intervention period (Ackatiah-Armah et al., 2014) and two studies measured WHZ at endline (Amegovu et al., 2014; Kajjura et al., 2019); [6]Children who remained MAM in the 12 week intervention period; [3]Developing SAM during the 12 week intervention period based either on WHZ<-3, MUAC<11.5cm and oedema;

Explanations for Evidence GRADE

a. Serious risk of bias: majority or all information is from studies at high or unclear overall risk of bias.
b. Serious imprecision: the 95% CI around the absolute effect crosses the null threshold, including both a small harm and trivial benefit.
c. Serious inconsistency: considerable heterogeneity I^2=87%
d. Serious imprecision: the 95% CI around the absolute effect considerably crosses the null threshold, including both appreciable benefit and harm.
e. Serious imprecision: the 95% CI around the absolute effect does not cross the null threshold but OIS is modestly breached.
f. Very serious imprecision: the 95% CI around the absolute effect crosses the null threshold, and includes both a large harm and a trivial benefit.
g. Serious inconsistency: considerable heterogeneity I^2=76%.
h. No imprecision: trivial difference and 95% CI (effect is not clinically meaningful)
i. Serious inconsistency: Considerable heterogeneity I2=77%.
j. Very serious imprecision: the 95% CI around the absolute effect crosses the null threshold, and includes both trivial benefit and moderate harm.

k. Very serious inconsistency: substantial heterogeneity I2=95%.

l. Very serious imprecision: the 95% CI around the absolute effect crosses the null threshold, including both a very large reduction in time to recovery and a small increase.
m. Serious imprecision: the 95% CI around the absolute effect considerably crosses the null threshold, including a small harm and trivial benefit.

Supplementary Table S6. Evidence Profile for comparison 2b.1 (RUTF vs RUSF)[1,2]

Population: 6-59 months old children with moderate wasting

Intervention: RUTF

Comparison: RUSF

Settings: Kenya and South Sudan

Certainty assessment							№ of patients		Effect		Certainty	Importance
№ of studies	Study design	Risk of bias	Inconsistency	Indirectness	Imprecision	Other considerations	RUTF	RUSF	Relative (95% CI)	Absolute (95% CI)		
**Anthropometric recovery- Number of children who achieved a MUAC ≥125mm during the intervention period**												
1	randomised trials	Serious[a]	not serious	not serious	not serious[b]	none	860/995 (86.4%)	773/908 (85.1%)	**RR 1.02** (0.98 to 1.05)	**17 more per 1,000** (from 17 fewer to 43 more)	⊕⊕⊕◯ Moderate	CRITICAL
**Deterioration to severe wasting – not reported**												
-	-	-	-	-	-	-	-	-	-	-	-	CRITICAL
**WHZ at endline (after 4 months) – not reported**												
-	-	-	-	-	-	-	-	-	-	-	-	CRITICAL
**WAZ at endline (after 4 months) – not reported**												

Certainty assessment							№ of patients		Effect		Certainty	Importance
№ of studies	Study design	Risk of bias	Inconsistency	Indirectness	Imprecision	Other considerations	RUTF	RUSF	Relative (95% CI)	Absolute (95% CI)		
**HAZ at endline (after 4 months) – not reported**												
-	-	-	-	-	-	-	-	-	-	-	-	CRITICAL
**MUAC in cm at endline (after 4 months)**												
1	randomised trials	serious [a]	not serious	not serious	serious[b]	none	268<br>Mean (SD):13.3 (0.7)	375<br>Mean (SD):13.4 (0.7)	-	MD **0.1 lower**<br>(0.21 lower to 0.01 higher)	⨁⨁⨁◯<br>Moderate	CRITICAL
**Weight gain in g/kg/day over the 4 months intervention period**												
1	randomised trials	serious[a]	not serious	not serious	serious[c]	none	268<br>Mean (SD):1.7 (0.8)	375<br>Mean (SD): 1.5 (0.7)	-	MD **0.2 higher**<br>(0.08 higher to 0.32 higher)	⨁⨁◯◯<br>Low	CRITICAL
**Height in cm at endline (after 4 months)**												
1	randomised trials	serious [a]	not serious	not serious	serious[d]	none	268<br>Mean (SD):76.6 (6.3)	375<br>Mean (SD): 77.2 (5.5)	-	MD **0.6 lower**<br>(1.54 lower to 0.34 higher)	⨁⨁◯◯<br>Low	CRITICAL
**Sustained recovery – data not shown**												
-	-	-	-	-	-	-	-	-	-	-	-	-
**Non-response – data not shown**												
-	-	-	-	-	-	-	-	-	-	-	-	-

Certainty assessment							№ of patients		Effect		Certainty	Importance
№ of studies	Study design	Risk of bias	Inconsistency	Indirectness	Imprecision	Other considerations	RUTF	RUSF	Relative (95% CI)	Absolute (95% CI)		
**Time to recovery (days) – data not shown**												
-		-	-	-	-	-	-	-	-	-	-	IMPORTANT
**Relapse** (number of recovered children relapsing to MAM or SAM)												
1	randomised trials	serious [a]	not serious	not serious	serious[c]	none	21/218 (9.6%)	39/318 (12.3%)	**RR 0.79** (0.48 to 1.30)	**26 fewer per 1,000** (from 64 fewer to 37 more)	⨁⨁◯◯ Low	IMPORTANT

[1] Abbreviations: CI: confidence interval; MD: mean difference; RR: risk ratio;

Explanations for Evidence GRADE

a. Serious risk of bias: all information is from a sub-sample in a trial with some concerns for overall risk of bias.
b. No imprecision: trivial difference and 95% CI (effect is not clinically meaningful)
c. Serious imprecision: 95% CI around the absolute effect does not cross null threshold, however it ranges from a trivial to small difference.
d. Serious imprecision: the 95% CI around the absolute effect crosses the null threshold and includes small harm and trivial benefit.
e. Serious imprecision: the 95% CI around the absolute effect crosses the null threshold and includes a small benefit and trivial harm.
